# Supplementary material for: An oral, liver-restricted LXR inverse agonist for dyslipidemia: preclinical development and phase 1 trial
Source: Nat Med. 2026 Jan 16;32(3):883–93. doi: 10.1038/s41591-025-04169-6 (PMC13004691; doi:10.1038/s41591-025-04169-6)
Supplement: Supplementary file 1 — Supplementary Fig. 1. Blank copy of the informed consent, approved study protocol for phase 1 clinical trial and CONSORT checklist. [file 41591_2025_4169_MOESM1_ESM.pdf]

nature medicine

Supplementary information https://doi.org/10.1038/s41591-025-04169-6

An oral, liver-restricted LXR inverse agonist for dyslipidemia: preclinical development and phase 1 trial

In the format provided by the authors and unedited

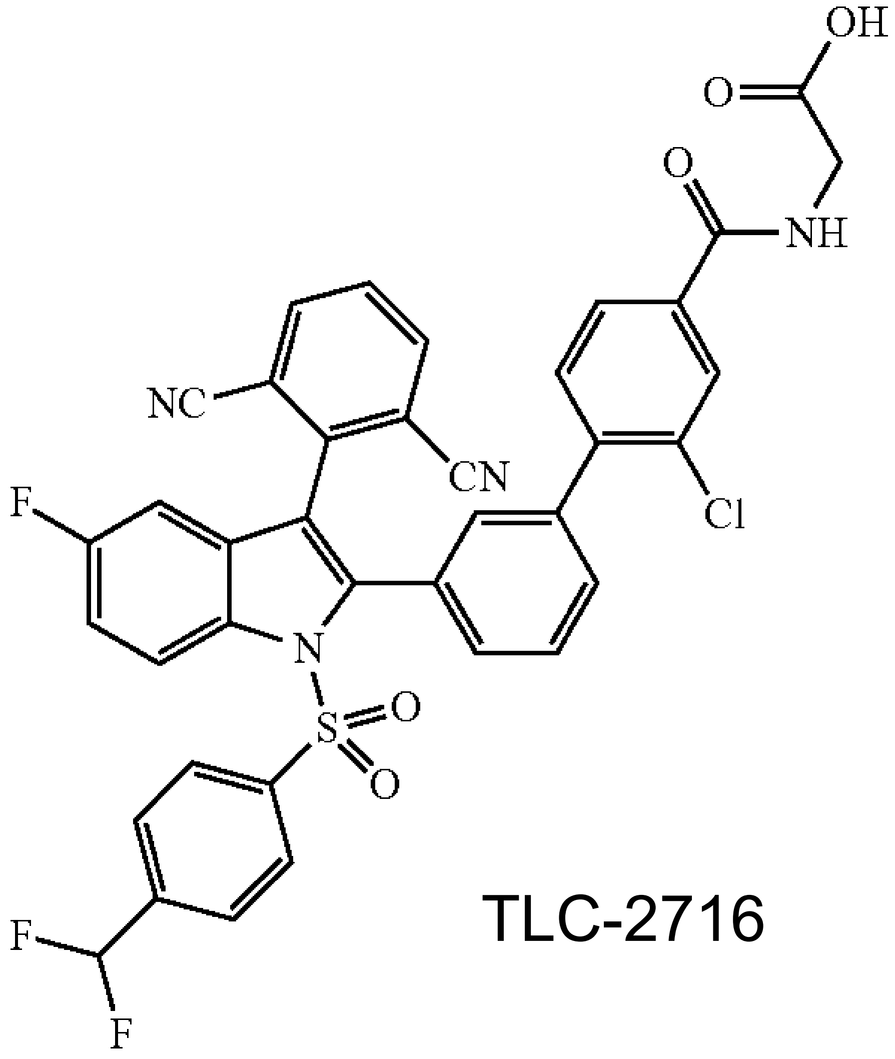

TLC-2716

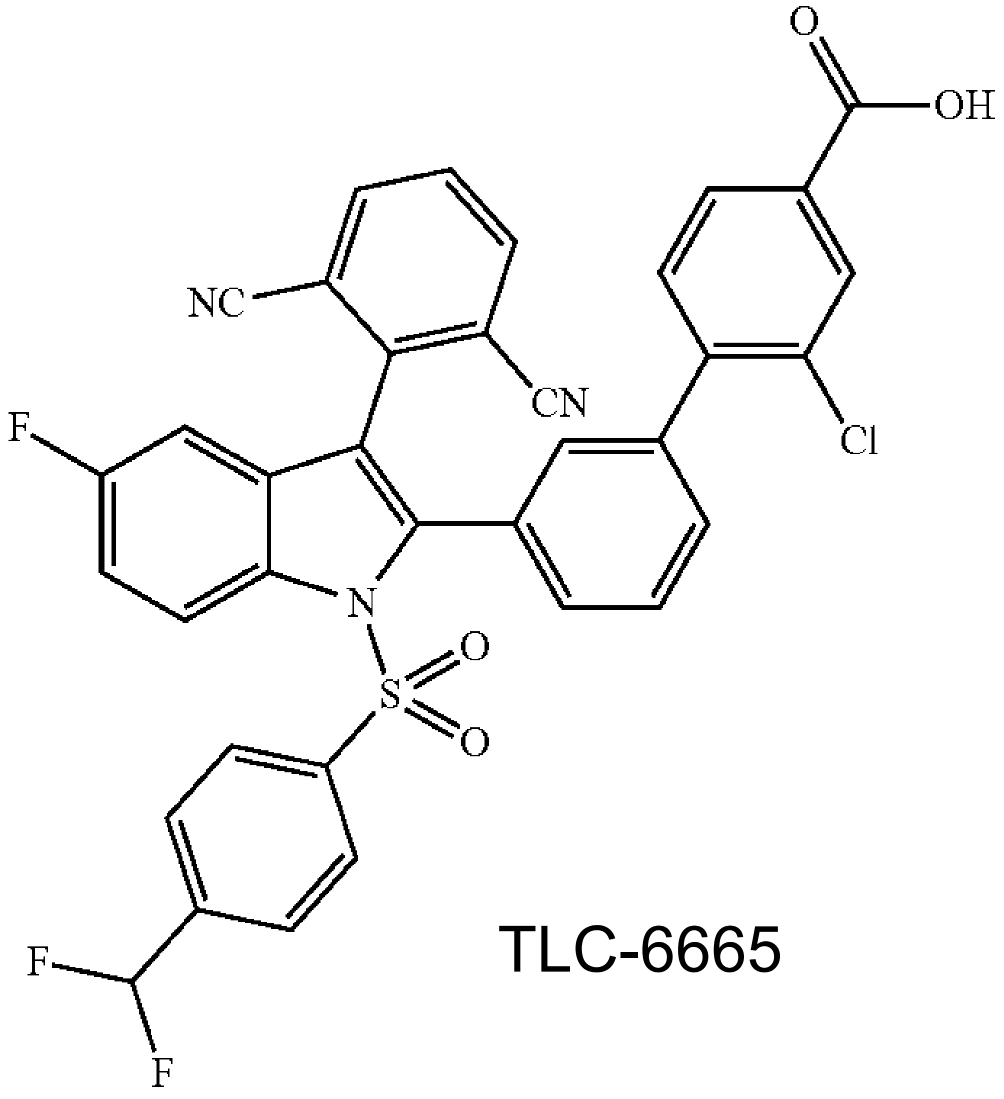

TLC-6665

Supplementary Fig. 1: Compound structure of LXR inverse agonists.

PARTICIPANT INFORMATION SHEET AND CONSENT FORM
(Part A: Healthy Volunteer – Single Ascending Dose)

**Short Title:**	A Study to Evaluate Single and Multiple Doses of TLC-2716 in Healthy Participants
**Protocol Number:**	2716-CL-101
**Sponsor:**	The Liver Company, Inc.<br>2671 Marshall Drive, Palo Alto, California, United States of America
**Principal Investigator:**	Prof. Ed Gane
**Institution Address:**	Main Building: 3 Ferncroft Street, Grafton, Auckland, New Zealand<br>Outpatient Unit: 125 Grafton Road, Grafton, Auckland, New Zealand
**Phone Number:**	0800 STUDIES (0800 788 3437)
**Ethics Number:**	**2022 FULL 12858**

This is the first time that TLC-2716 will be studied in humans.
You will not get any health benefits from the drug used in this study; but there are risks of you having a drug reaction, injury, or illness.

You are invited to take part in a clinical research study. This study will test an investigational drug, named TLC-2716, that may potentially be used for the treatment of severe dyslipidaemias and non-alcoholic steatohepatitis (NASH). TLC-2716 is an investigational drug because it has not been approved by the New Zealand MedSafe or other regulatory authorities.

There are multiple parts to this study, and you are being asked to take part in Part A. This Participant Information Sheet will help you decide if you'd like to take part. It sets out why we are doing the study, what your participation would involve, what the benefits and risks to you might be, and what would happen after the study ends. We will go through this information with you and answer any questions you may have. We expect this will take about 30-60 minutes. You do not have to decide today whether or not you will participate in this study. Before you decide you may want to talk about the study with other people, such as family, whānau, friends, or healthcare providers. Feel free to do this.

Whether or not you take part is your choice. If you don't want to take part, you don't have to give a reason, and it won't affect the care you receive. If you do want to take part now, but change your mind later, you can pull out of the study at any time.

If you agree to take part in this study, you will be asked to sign and date the Consent Form on the last page of this document. You will be given a copy of both the Participant Information Sheet and the Consent Form to keep.

This document is **20** pages long, including the Consent Form. Please make sure you have read and understood all the pages.

1 WHY ARE WE DOING THE STUDY?

1.1 Purpose

TLC-2716 is being developed for the treatment of severe dyslipidaemias and NASH. Dyslipidaemia refers to unhealthy levels of one or more kinds of lipid (fat) in the blood. NASH is an advanced form of non-alcoholic fatty liver disease (NAFLD), which is caused by a build-up of fat in the liver. When NAFLD causes inflammation and damage in the liver, it progresses to NASH, which can then lead to liver cirrhosis (scarring of the liver), liver failure, and sometimes liver cancer. NASH is strongly linked to other conditions such as obesity, dyslipidaemia, high blood pressure, and type 2 diabetes. The prevalence of NASH is increasing worldwide, and the currently available treatment options are limited.

TLC-2716 works by inhibition (disruption the normal reaction) of a receptor in liver cells called liver X receptor (LXR) and is a key factor in regulation and metabolism of lipids, allowing the receptors to maintain normal or lower cholesterol and triglyceride levels throughout the body. It is hoped that, by maintaining or lowering cholesterol and triglyceride levels, TLC-2716 may be an effective treatment for severe dyslipidaemias or NASH.

This study will investigate the effects of single doses (Parts A and C) or multiple doses (Parts B and C) of TLC-2716 in healthy participants. The purpose of Part A of this study is to:

- Evaluate how safe and well tolerated a single dose of TLC-2716 is, in healthy participants.
- Measure levels of TLC-2716 in the blood over time, following a single dose of TLC-2716 (pharmacokinetics/PK).
- Measure the body's response to a single dose of TLC-2716 (pharmacodynamics/PD).

1.2 Study Design

Up to 150 healthy adults will take part in this study; 50 participants will be enrolled in Part A, 50 in Part B, and 50 in Part C. You are being asked to participate in Part A.

The study requires a 4-night stay at the New Zealand Clinical Research (NZCR) research unit and 1 scheduled clinic visit.

This is a randomised, blinded placebo-controlled study:

Randomised means that the study treatment you take (drug or placebo) will be assigned randomly (by chance).

Blinded means that neither you nor your study doctor will know whether you will be receiving TLC-2716 or placebo. In an emergency, the study doctor can find out what you are receiving.

Every participant in Part A of the study will receive a single dose of TLC-2716 or placebo (a capsule that looks like TLC-2716 but contains no medicine). Each dose will be given by mouth, with a glass of water after a meal.

Five dose groups (cohorts) are planned for the study, with 10 participants in each cohort. If you are eligible for this study, you will be assigned to a cohort. The cohort you are assigned to will depend on when you join the study. You do not have a choice as to which cohort you are assigned. You are only allowed to participate in one cohort. Details for the cohorts are as follows:

- **Cohort 1:** 0.5 mg TLC-2716 or placebo
- **Cohort 2:** 2 mg TLC-2716 or placebo
- **Cohort 3:** 6 mg TLC-2716 or placebo
- **Cohort 4:** 12 mg TLC-2716 or placebo
- **Cohort 5:** 20 mg TLC-2716 or placebo

In each cohort, two participants will be dosed first (one will receive TLC-2716 and the other will receive placebo). These participants are called ‘sentinels. The sentinel participants will be monitored for approximately 2 days, and if there are no safety concerns, the next 8 participants will be dosed (7 participants will receive TLC-2716 and 1 will receive placebo). You will have a 4 out of 5 (80%) chance of getting TLC-2716 and 1 out of 5 (20%) chance of getting placebo.

Cohorts will be enrolled in order, where the next cohort testing a higher dose of TLC-2716 will be started if there are no safety concerns in the previous lower dose cohort. The Sponsor can hold dosing, or select a lower dose for a cohort, based on the effects of TLC-2716. You will be told if any changes are made to the planned dose for your cohort.

Blood samples and other tests to measure study drug levels and effects on the body will be collected at specific time points during the study, your safety will be monitored, and any changes in your health will be recorded.

1.3 Nature and Sources of Funding of the Study

This research study is being sponsored globally and funded by The Liver Company, Inc. and conducted locally in New Zealand at NZCR.

By taking part in this research study, you agree that data generated from your assessments throughout the study, will be provided to the Sponsor. The knowledge gained from this data may lead to new discoveries, which would assist them in obtaining approval for a new drug and benefit the sponsor financially. There would be no financial benefit to you from these discoveries.

NZCR will receive a payment from The Liver Company, Inc. for undertaking this research study.

No member of the research team will receive a personal financial benefit from your involvement in this research study (other than their ordinary wages).

1.4 Approval by Ethics Committee

This study has been reviewed by an independent group of people called a Health and Disability Ethics Committee (HDEC). The ethical aspects of this research study have been approved by the **Northern B** Ethics Committee.

A description of this clinical study will be available on http://www.ClinicalTrials.gov. This web site will not include information that can identify you. At most, the web site will include a summary of the results. You can search this web site at any time.

2 WHAT WOULD YOUR PARTICIPATION INVOLVE?

Participation in this study will last up to approximately 6 weeks, which includes the screening, treatment, and follow-up periods. If you wish to participate in this study, you will be asked to sign this consent form before any study assessments can be performed.

The details of the study and tests performed are shown in the table below. During the screening period, assessments will be done to check that you meet the requirements to be a participant in the study. The day you have your dose of TLC-2716 is called Day 1 and all other study days are counted backward or forward from this day.

The results of the screening assessments will determine whether or not you can take part in the study. Entry into screening does not guarantee enrolment into the study. We may screen more participants than we need and so you may be asked to be a standby or a backup. This means you will be asked to come to the clinic and undergo the study tests and procedures until we have enrolled enough eligible participants for a cohort. You will then be discharged and where possible we will try to include you in a later cohort.

<table>
<tr><th colspan="2">SCREENING</th></tr>
<tr><td>Screening Visit

Day -28 to Day -2
Approx. 2 hours</td><td>Before any study procedures are performed, you will be asked to sign this informed consent form. Then the following assessments will be done:
Review your health history
Complete physical examination
Height and weight
Set of vital signs (blood pressure, heart rate, temperature)
An ECG (to measure the electrical activity of your heart)
Set of blood and urine samples for routine health tests
Urine drug screen and alcohol breath test
Viral infection blood tests (human immunodeficiency virus (HIV), hepatitis B virus (HBV), and hepatitis C virus (HCV)
COVID-19 testing (and during the study if necessary)
Pregnancy blood test (for women who are able to have children) or FSH test (to prove post-menopausal status)</td></tr>
<tr><td colspan="2">A decision about whether you can take part will be made when all your screening results are available</td></tr>
<tr><th colspan="2">TREATMENT PERIOD</th></tr>
<tr><td>Inpatient Stay

Day -1 to Day 4</td><td>Day -1 (Admission):
Complete physical examination
Weight
Set of vital signs
An ECG
Set of blood and urine samples for routine health tests
Urine drug screen and alcohol breath test
Urine pregnancy test (for women who are able to have children)
Day 1:
Single dose of TLC-2716 or placebo
Physical exam (based on your symptoms)
Set of vital signs
Sets of blood and urine samples for routine health tests
Sets of ECG’s
Blood samples to measure level of study drug in your blood (PK)
Blood samples to measure markers of biological activity and the effects of the drug on the body (biomarkers and Pharmacodynamics/PD)
Days 2 & 3:
Physical exam (based on your symptoms)
Set of vital signs
Set of safety blood and urine samples
An ECG (Day 2 only)
Blood samples to measure PK
Day 4 (Discharge):
Complete physical examination
Weight
Set of vital signs
Physical examination
Set of blood and urine samples
Pregnancy blood test (for women who are able to have children)
An ECG
Blood samples to measure PK
You will be discharged from the unit, following a study doctor’s review.</td></tr>
</table>

FOLLOW UP PERIOD	
**Follow-up Visit**<br>Day 15<br>Approx. 1 hour	• Complete physical examination<br>• Weight<br>• Set of vital signs<br>• An ECG<br>• Set of blood and urine samples for routine health tests<br>• Pregnancy blood test (for women who are able to have children)
**You will be discharged from the study once your results have been checked by a study doctor.**<br>**END OF STUDY**	

2.1 Who Can Take Part in this Study?

To take part in this study you must:

- Be able to give consent and follow the study procedures.
- Be between the ages of 18 – 55 years, inclusive.
- Be in good health
- Have a body mass index (BMI) between 19.0 kg/m^2 – 35.0 kg/m^2.
- Be a non-smoker.

You cannot take part in this study if you:

- Are pregnant or breastfeeding.
- Have taken any prescription, over-the-counter medications, or herbal products (excluding contraceptives, ibuprofen, paracetamol or vitamins) within 28 days prior to dosing.
- Have used systemic steroids (e.g. prednisone or oral corticosteroids), immunosuppressive therapy, or chemotherapy within 3 months of screening, or are expecting to need these during the study.
- Have current alcohol or substance abuse.
- Have a history of a significant medical problem, mental health problem or severe allergy.
- Have a history of serious skin disease such as rashes/itching, food allergies, eczema, or psoriasis
- Have any history of significant heart problems, have a family history of long QT syndrome (a heart rhythm condition that can potentially cause fast, chaotic heartbeats).
- Have a history of fainting, heart palpitations, or unexplained dizziness.
- Have an implantable defibrillator or pacemaker.
- Have a have a history of liver disease.
- Have a history of severe peptic ulcer disease (open sores that develop on the inside lining of your stomach and the upper portion of your small intestine), or other gastric acid conditions such as gastric reflux.
- Have a history of medical or surgical treatment on the stomach or bowel (gallbladder removal is not exclusionary).
- Have received an investigational study drug in another clinical trial within 30 days, or potentially longer depending on the investigational drug, of your dose of TLC-2716 or placebo.
- Have donated blood within 56 days prior to admission

2.2 Study Instructions

It is important that you follow the instructions you are given and attend all scheduled clinic visits. It is also possible that the study doctor may schedule extra visits or tests for you, if considered necessary.

You will be given a Participant Identification Card if you participate in this study, stating the name of the study and the study doctor's contact information. This card should be carried with you at all times: you can contact the study doctor at any time or use it to inform any other doctor, dentist, or pharmacist that you are participating in this study.

At each visit, the study staff will ask you questions about your health and the medications you are currently taking. You should always report any changes in your health, unusual feelings, or symptoms to the study staff. It is also important that you do not start, stop, or make changes to any of the medications you are currently taking without first discussing this with your study doctor.

All of your meals will be provided during your inpatient stay. Meals play an important role in clinical trials due to their relationship with study drug metabolism (the way that the drug is processed and broken down by the body). By signing the informed consent form, you are agreeing to be compliant with all meal requirements on this study.

Study Restrictions:

- You will not be able to smoke or use any nicotine-containing products from 90 days prior to admission (Day -1) until after your final follow up visit.
- You must not consume any alcohol from at least 72 hours prior to admission until after your final follow up visit.
- You must not consume any food or beverages containing grapefruit, grapefruit juice, Seville oranges or orange juice for at least four days prior to admission and until after your final follow up visit.
- You must be fasted (no food, only water) for at least 10 hours prior to your screening visit, admission, and your follow up visit. Study staff will remind you prior to each visit that you need to be fasted.
- You are encouraged to avoid strenuous exercise, as well as saunas, steam baths, sunbathing, or prolonged UV exposure (e.g., tanning beds), from screening until after your final follow up visit.
- You must not donate blood from 56 days prior to admission or plasma from 7 days prior to admission, until 30 days following the dose of study drug.

The Investigator may request a random check of your bag(s) at admission for prohibited items (e.g., drinks or foods). Any prohibited items will be removed and returned to you on discharge from the unit.

3 WHAT ARE THE POSSIBLE BENEFITS AND RISKS TO YOU PARTICIPATING?

3.1 Benefits

You will not receive any direct medical benefits from taking part in this study. However, information learned from this study might help to develop better treatments for severe dyslipidaemias and NASH.

3.2 Reimbursement and Costs

All tests required to be done for this study will be paid for by The Liver Company, Inc. and there will be no cost for you to participate in this study.

You will be reimbursed the sum of $3,500 gross, following the final follow up visit**. We are required to deduct withholding tax at the applicable rate you have declared in the IR330C form**. You will be responsible for

any other tax obligations (e.g., ACC). If you are GST registered, it may be possible for you to submit a GST invoice. Contact paymentforms@nzcr.co.nz if you would like to discuss this further.

The payment will be made after you complete the study, to cover your time and inconvenience. If you are receiving a benefit or allowance, your usual payments may be affected. Your tax statement will state that you were paid from your dosing day through to your final study visit.

You will be reimbursed for travel and parking for your study visits if you live in the metropolitan area. If you live outside this area, we will discuss your travel costs individually. Full reimbursement requires completion of all visits according to study requirements. If you are withdrawn from the study for medical reasons as a result of treatment given as part of this study, you will receive reimbursement in full.

If you leave the study of your own choice or are released from the study for non-medical reasons, you will receive a partial reimbursement (a pro-rata reimbursement) based on how many study days you completed. If you are invited to participate in the study as a Stand-by or Back-up participant and not enrolled in the study, you will receive reimbursement ($350 and $150, respectively) for your time and inconvenience. If you completed the screening visit assessments but were not eligible to enrol in the study, you will not receive any reimbursement.

3.3 Possible Risks and Disadvantages?

Medical treatments may cause side effects. You may have none, some, or all of the side effects listed below. The side effects may feel mild, moderate, or severe. There may also be unknown side effects from taking TLC-2716 alone or with other drugs you may be taking. If you have any of these side effects, or are worried about them, talk with your study doctor. Your study doctor will also be looking out for side effects.

<u>What are the Risks or Side Effects of TLC-2716?</u>

TLC-2716 Common Adverse Events:

This is the first time that TLC-2716 is being studied in humans. As such, there are unknown risks involved in receiving TLC-2716.
TLC-2716 was tested in animals with doses that are much higher than what will be tested in this study. In animal studies, the below side effects were observed, all of which were considered mild and reversible:

- Slight body weight loss
- Occasional soft and watery stools (diarrhea)

Allergic Reaction:
An immune reaction or allergic reaction is always possible with a drug you have not taken. Serious allergic reactions that can be life-threatening may occur. Some things that happen during any allergic reaction to any type of medication are:
- Rash
- Having a hard time breathing
- Wheezing when you breathe
- Sudden drop in blood pressure
- Swelling around the mouth, throat, or eyes
- Fast heart rate
- Sweating
- Itching
- Pain in your abdomen
- Diarrhea
- Seizures

What are the Risks or Side Effects of Study Procedures?

Blood Sample Collection & Cannulas:
Risks include bruises, swelling with itching, and slight bleeding. The area may become inflamed. In rare cases, it may result in a blood clot, an infection or nerve damage. As needles can cause pain, you may feel light-headed or faint.

ECG Tests:
Sometimes the sticky pads used to attach the ECG leads can cause mild skin irritation (slight redness / itchiness).

Physical Examination:
During this examination you may be asked to remove some items of clothing so a full examination can be done. You can request a chaperone to be with you at the time of the physical examination, please ask one of our research nurses.

COVID-19 Vaccination

COVID-19 has been declared a pandemic by the World Health Organisation (WHO) and it is affecting nearly every community. During this study, you may not receive a COVID-19 vaccination or booster vaccination. Additionally, any vaccination must be completed at least 14 days prior to admission into the research unit.

3.4 Contraception

Reproductive Risks for Sexually Active Participants of Child-Bearing Potential

The effects of TLC-2716 in pregnancy and breastfeeding are unknown, but there is a risk it may cause birth defects or foetal deaths, and/or be passed on in breast milk. If you are pregnant or breastfeeding, you cannot take part in this study.

If you are sexually active and of child-bearing potential (able to become pregnant), it is very important that you do not become pregnant during this study. **You must use one of the methods of contraception listed below**, from at least screening until at least 30 days after your dose:

A woman of child-bearing potential is any pre-menopausal woman who may become pregnant. If you are unsure if this applies to you, please check with the study doctor before you start the study treatment.

A highly effective method (less than 1 pregnancy per 100 women using the method for one year) e.g.:

- Implant contraceptive (e.g., Jadelle®)
- Intra-uterine device (IUD) containing either copper or levonorgestrel (e.g., Mirena®)
- Male sterilisation (vasectomy)
- Female sterilisation (e.g., by bilateral tubal ligation ('clipping or tying tubes' or hysterectomy)

OR an effective method (5-10 pregnancies per 100 women using the method for one year) e.g.:

- Injectable contraceptive (e.g., Depo Provera)
- Oral Contraceptive Pill (combined hormonal pill or progestogen-only 'mini-pill')
- Vaginal contraceptive ring (e.g., NuvaRing®)

You / your partner must also use a barrier form of contraception if you are using an effective method of contraception, from your screening through until 30 days after your dose. Barrier methods of contraception include:

- Male condoms
- Female condoms
- Female diaphragm ('cap')

Please note that barrier methods alone are not highly effective methods of birth control.

Total abstinence from heterosexual intercourse during the entire period of risk associated with the study drug (from screening until at least 30 days after your last dose) is considered an acceptable form of contraception if this is in line with your preferred and usual lifestyle.

If you are unsure which method of birth control you are using (or want to start using) and whether it is acceptable for this study, please ask the study doctor for more information.

You must also agree to not donate eggs, from dosing (Day 1) until at least 30 days following the dose of study drug.

If you do become pregnant during the study, you must tell the study doctor as soon as possible. If you do become pregnant you will be asked to sign a separate consent form, to allow the Sponsor to collect information about your pregnancy and the outcome of your pregnancy.

Reproductive Risks for Sexually Active Participants able to Father a Child

The effects of TLC-2716 if passed on through semen are unknown, but there is a risk it may cause birth defects or foetal deaths. **You are responsible for informing your sexual partner of these possible risks.**

If you are sexually active and have any partner who is of child-bearing potential (meaning a partner who may become pregnant) it is very important that you use contraception during this study. It is highly recommended that you and your partner use one of the contraception options listed above for participants of child-bearing potential, from at least dosing of the study drug through until at least 90 days after your dose.

You / your partner must also use a male condom method of contraception, from your dose of study drug through until 90 days after your dose.

Please note that barrier methods alone are not highly effective methods of contraception.

Total abstinence from heterosexual intercourse during the entire period of risk associated with the study drug (from dosing until at least 90 after your dose) is considered an acceptable form of contraception if this is in line with your preferred and usual lifestyle.

If a pregnancy occurs, you must report this to the study doctor as soon as possible. Your partner will be asked to give consent for her information and her infant's information to be collected for monitoring purposes.

You must also agree to not donate sperm, from dosing (Day 1) until 90 days following the dose of study drug.

4 WHAT WOULD HAPPEN IF YOU WERE INJURED IN THE STUDY?

As this research study is for the principal benefit of its commercial sponsor, The Liver Company, Ltd, if you are injured as a result of taking part in this study you **won't** be eligible for compensation from ACC.

However, The Liver Company has satisfied the **Northern B** Health and Disability Ethics Committee that approved this study that it has up-to-date insurance for providing participants with compensation if they are injured as a result of taking part in this study.

New Zealand ethical guidelines for intervention studies require compensation for injury to be at least ACC equivalent. Compensation should be appropriate to the nature, severity and persistence of your injury and should be no less than would be awarded for similar injuries by New Zealand's ACC scheme.

Some sponsors voluntarily commit to providing compensation in accordance with guidelines that they have agreed between themselves, called the Medicines New Zealand Guidelines (Industry Guidelines). These are often referred to for information on compensation for commercial clinical trials. There are some important points to know about the Industry Guidelines:

- On their own they are not legally enforceable and may not provide ACC equivalent compensation.
- There are limitations on when compensation is available, for example compensation may be available for more serious, enduring injuries, and not for temporary pain or discomfort or less serious or curable complaints.
- Unlike ACC, the guidelines do not provide compensation on a no-fault basis:
- The Sponsor may not accept the compensation claim if:
    - Your injury was caused by the investigators, or;
    - There was a deviation from the proposed research plan, or;
    - Your injury was caused solely by you.

An initial decision whether to compensate you would be made the by the sponsor and/or its insurers.

If they decide not to compensate you, you may be able to take action through the Courts for compensation, but it could be expensive and lengthy, and you might require legal representation. You would need to be able to show that your injury was caused by participation in the trial.

You are strongly advised to read the Industry Guidelines and ask questions if you are unsure about what they mean for you.

If you have private health or life insurance, you may wish to check with your insurer that taking part in this study won't affect your cover.

5 WHAT WILL HAPPEN TO MY TEST SAMPLES?

Blood and urine samples will be collected throughout the study. These samples will be used for various tests. Some of the samples will be used for regular routine blood counts and blood chemistry, and to monitor your general health.

All these routine samples will be sent to LabPlus for testing and destroyed after 3 months by internationally accepted means.

All other study samples (PK, PD and biomarkers) will be sent to central laboratories in Colorado Springs, Colorado, USA (Pyxant Labs), Singapore (LabCorp), Melbourne, Australia (360biolabs), and Adelaide, Australia (GNOMIX) for testing and destroyed after 3 years by internationally accepted means

The maximum amount of blood collected from each participant during the study will be up to 210 mL. For comparison, a standard blood donation at a blood collection centre, is about 470 mL. Samples collected by NZCR will be identified by your study number, year of birth, initials, and sex, to allow study doctors to quickly respond to any abnormal results. Before the results of these tests are sent to the Sponsor, your identifiable information will be removed and replaced with a code.

The proposed blood tests include a screening test for HIV and for viral Hepatitis B and C, as well as a COVID-19 test. Signing the consent form means that you agree to have this testing performed. It is important to understand that a positive screening test does not necessarily mean you have the disease. Should you receive a positive screening result for HIV or either Hepatitis B or C, then the study doctors will provide initial counselling and medical advice and will assist in arranging any follow up tests that you require. HIV, Hepatitis B and C, and COVID-19 are all 'notifiable diseases', which means that it is required by law to notify government health authorities of any new cases.

5.1 Are There Any Cultural Considerations?

You may hold beliefs about sacred and shared values about your tissue samples and/or data originating from this tissue. The cultural issues associated with sending your tissue samples and data overseas and/or storing your tissue and data should be discussed with your family/whanau as appropriate. If you need cultural support this can be provided. Please let us know and we will arrange this for you, or you can ring the Māori cultural support number at the bottom of this Participant Information Sheet and Consent Form. Cultural support is different to knowing more about the study. In these cases, we can arrange for an Investigator to come and talk to you and your whānau. Due to your samples being sent to countries outside of New Zealand, a karakia will not be able to be performed at the time of your sample disposal.

Personal and health information is a tāonga and will be treated accordingly. The following data sovereignty principles are in place to ensure that the data generated from this research is protected and may benefit Māori now and into the future. Clinical trials are often driven by Sponsor companies overseas and involve very few Māori as participants. However, NZCR will consider the principles of whakapapa, whanaungatanga, rangatiratanga, kotahitanga, manaakitanga, and kaitiakitanga throughout the study.

6 WHAT ARE THE RIGHTS OF PARTICIPANTS IN THE STUDY?

6.1 Participation is Voluntary

Participation in any research study is voluntary. If you do not wish to take part, you do not have to. If you decide to take part and later change your mind, you are free to withdraw from the study at any stage. This will not affect your routine treatment/medical care which you may otherwise receive, or with NZCR.

If you do decide to take part, you will be given this Participant Information and Consent Form to sign and you will be given a copy to keep.

6.2 New Information

Sometimes during the course of a research study, new information becomes available about the treatment that is being studied. If this happens, your study doctor will tell you about it and discuss with you whether you want to continue in the research study. If you decide to withdraw, your study doctor will make arrangements for your regular health care to continue. If you decide to continue in the research study you may be asked to sign an updated consent form.

Also, on receiving new information, your study doctor might consider it to be in your best interests to withdraw you from the research study. If this happens, he/ she will explain the reasons and arrange for your regular health care to continue.

6.3 Privacy and Confidentiality and Right to Access Information Collected During the Study

What will Happen to my Information?
During this study, the study doctors, researchers, nurses and other NZCR staff will record information about you and your study participation. This includes the results of any study assessments. Your name, address, and phone number will be on the demographics page for the study so we can identify you correctly at visits and contact you. This information may be used to obtain health records (detailed below) but is not supplied to anyone overseas. If needed, information from your hospital records and your usual doctor (GP) may also be collected, and your GP may be notified about your participation in the study. You cannot take part in this study if you do not consent to the collection of this information.

Identifiable Information
Identifiable information is any data that could identify you (e.g., your name, date of birth, or address). The following groups may have access to your identifiable information:

- NZCR staff (to complete study assessments).
- Local laboratory staff, to process and report your screening and safety tests.
- Study monitors authorised by the Sponsor, to make sure the study is being run properly and that the data collected is accurate.
- Sponsor and its authorised representatives if you make a compensation claim as a result of study-related injury. Identifiable information is required to assess your claim.
- Sponsor, its authorised representatives, ethics committees, or government agencies from New Zealand or overseas, if the study and/or NZCR is audited. Audits are done to make sure that participants are protected, the study is run properly, and the data collected is correct.
- Your GP, if a study test gives an unexpected result that could be important in terms of your health. This allows appropriate follow-up to be arranged.
- The Medical Officer of Health, if you return a positive test for COVID-19, HIV, HBV, or HCV.

Rarely, it may be necessary for the study doctor to share your information with other people – for example, if there is a serious threat to public health or safety, or to the life or health of you or another person OR if the information is required in certain legal situations.

De-identified (Coded) Information
To make sure your personal information is kept confidential, information that identifies you will not be included in any study information sent to, or generated by, the Sponsor. Instead, you will be identified by a code. NZCR will keep a list linking your code with your name, so that you can be identified by your coded data if needed.

The following groups may have access to your coded information, which will be sent and stored overseas:

- The Sponsor, for the purposes of this study.
- People and companies working with or for the Sponsor, for the purposes of this study.
- Regulatory or other governmental agencies worldwide.

Anonymised Information
The Sponsor may remove the code from your de-identified information – this is called 'anonymisation'. This makes it very difficult (but not impossible) to identify the information that belongs to you. The Sponsor may use this information for future research (see below).

Future Research Using Your Information
Your coded information may be used for future research related to the development of TLC-2716 and/or relating to severe dyslipidaemias, NASH, or similar diseases.

This future research may be conducted overseas. You will not be told when future research is undertaken using your information. Your information may be shared widely with other researchers or companies. Your information may also be added to information from other studies, to form much larger sets of data.

You will not get reports or other information about any / some research that is done using your information.

Your information may be used indefinitely for future research unless you withdraw your consent. However, it may be extremely difficult or impossible to access your information or withdraw consent for its use once your information has been shared for future research.

Security and Storage of Your Information
During the study, your information will be stored on paper forms at NZCR and electronically on secure servers. When the study has finished, paper forms will be transferred to a secure site and stored for at least 15 years, then destroyed. De-identified information in electronic form will remain on a secure platform and will be retained indefinitely. Storage will comply with local and/or international data security guidelines.

Risks
Although efforts will be made to protect your privacy, absolute confidentiality of your information cannot be guaranteed. Even with coded and anonymised information, there is no guarantee that you cannot be identified. While the risk is currently very small, the chance that someone might access and misuse your information (for example, by making it harder for you to get or keep a job or health insurance) might increase in the future as people find new ways of tracing information.

Your coded information is being sent overseas. Other countries may have lower levels of data protection than New Zealand. There may be no New Zealand representation on overseas organisations which make decisions about the use of your information. There is a risk that overseas researchers may work with information in a way that is not culturally appropriate for New Zealanders.

Rights to Access Your Information and Results
You have the right to request access to your information held by the research team. You also have the right to request that any information you disagree with is corrected.

Please ask if you would like to access your screening and safety tests during the study. You may access other study-specific information before the study is over, but this could result in you being withdrawn from the study to protect the study's scientific integrity.

If you have any questions about the collection and use of information about you, you should ask a study doctor.

7 WHAT WILL HAPPEN AFTER THE STUDY ENDS, OR IF I WANT TO PULL OUT?

7.1 If You Decide to Withdraw

You may withdraw your consent for the collection and use of your information at any time, by informing your study doctor. Please notify a member of the research team before you withdraw. This notice will allow that person or the research supervisor to discuss any health risks or special requirements linked to withdrawing, such as follow up visits.

If you withdraw your consent, your study participation will end, and the study team will stop collecting information from you. Information collected up until your withdrawal from the study will continue to be used and included in the study. This is to protect the quality of the study.

7.2 Why the Study Might be Unexpectedly Stopped

This research study may be stopped unexpectedly for a variety of reasons. These may include a decision by the study sponsor or unacceptable side effects observed in the study cohorts prior to or during your participation.

If you withdraw your consent, your study participation will end, and the study team will stop collecting information from you. Information collected up until your withdrawal from the study will continue to be used and included in the study. This is to protect the quality of the study.

7.3 Results

When the study ends the data must be analysed, so the results of the study may not be available until about a year after the research finishes. The study doctors and/or Sponsor may decide to discuss or publish the results of the study. This may include publication in journals, presentation at conferences or other professional forums. In any publication, information will be provided in such a way that you cannot be identified. Results of the study will be provided to you. If you do not wish to receive a summary of the study results when they become available, then please inform the NZCR staff.

Appendix 1. Schedule of Assessments

Period	Screening	Treatment/In-patient stay					Follow-Up
**Study Day**	**-28 to -2**	**-1**	**1**	**2**	**3**	**4**	**EOS 15**
Admission to the unit		X					
Discharge from the unit						X	
Physical Exam	X	X	As required (symptom-directed)			X	X
Vital Signs	X	X	X	X	X	X	X
ECG	X	X	X	X		X	X
BMI (Height & Weight)*[1]	X	X				X	X
Drug and Alcohol Testing	X	X					
COVID-19 Testing	X	X	As necessary				
Pregnancy Blood/Urine[2] Test (for women who are able to have children)	X	X				X	X
FSH Test (if applicable for post-menopausal females)	X						
Hepatitis/HIV Testing	X						
Dose Administration			X				
Routine Blood & Urine Tests	X	X	X	X	X	X	X
Biomarkers/PD Blood Tests			X				
PK Blood & Urine Tests			X	X	X	X	

BMI = body mass index; ECG = electrocardiogram; EOS = end of study; FSH = follicle stimulating hormone; ; PD = pharmacodynamics; PK = pharmacokinetics

[1] Height is only taken at screening
[2] Urine pregnancy test is done at admission, pregnancy blood test is done at all other time points.

8 WHO DO I CONTACT FOR MORE INFORMATION OR IF I HAVE CONCERNS?

If you have any questions, concerns, or complaints about the study at any stage, you can contact:

Prof Ed Gane, Principal Investigator
Phone: (09) 373 3474 or 0800STUDIES (08007883437)
Email: waterfall@nzcr.co.nz

If you want to talk to someone who isn't involved with the study, you can contact an independent health and disability advocate on:

Phone:	0800 555 050
Fax:	0800 2 SUPPORT (0800 2787 7678)
Email:	advocacy@advocacy.org.nz
Website:	https://www.advocacy.org.nz/

Māori cultural support is available through:

The Office of the Chief Advisor Tikanga, He Kamaka Waiora, Waitematā and Auckland District Health Board:

Mobile:	021 0203 1167
Phone:	09 486 8320 ext 43204
Email:	hkwresearch@waitematadhb.govt.nz

You can also contact the health and disability ethics committee (HDEC) that approved this study on:

Phone:	0800 4 ETHIC (438 442)
Email:	hdecs@health.govt.nz

9 WHAT ABOUT ANY OTHER QUESTIONS I MAY HAVE?

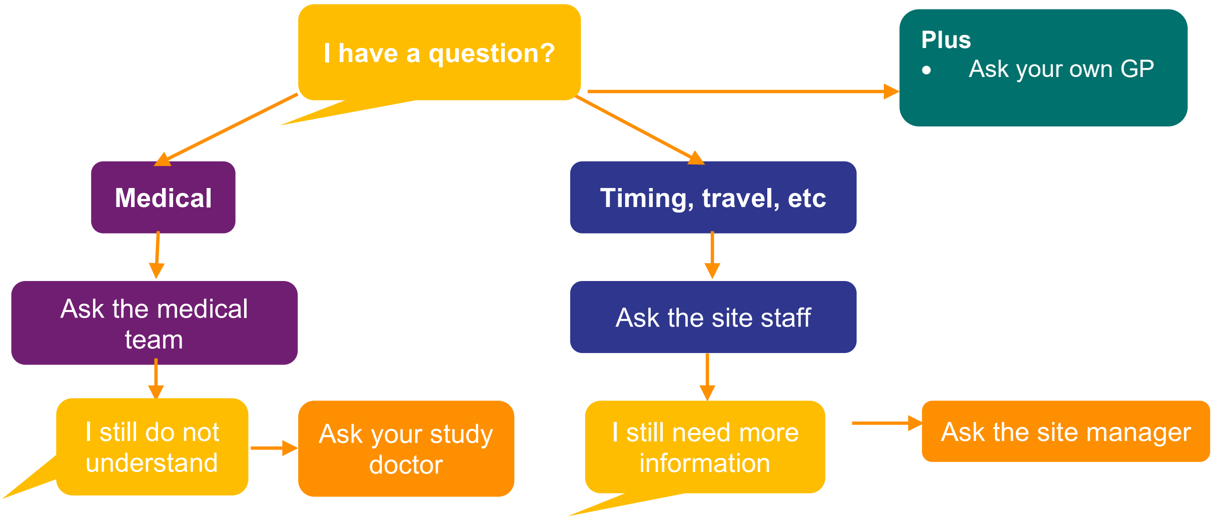

10 DO I HAVE TO DECIDE STRAIGHT AWAY?

No, you do not have to decide straight away. You should take some time to consider whether or not to participate in the study. We will be in touch in a week or so to discuss your decision**.** The following steps are useful in helping you reach a decision.

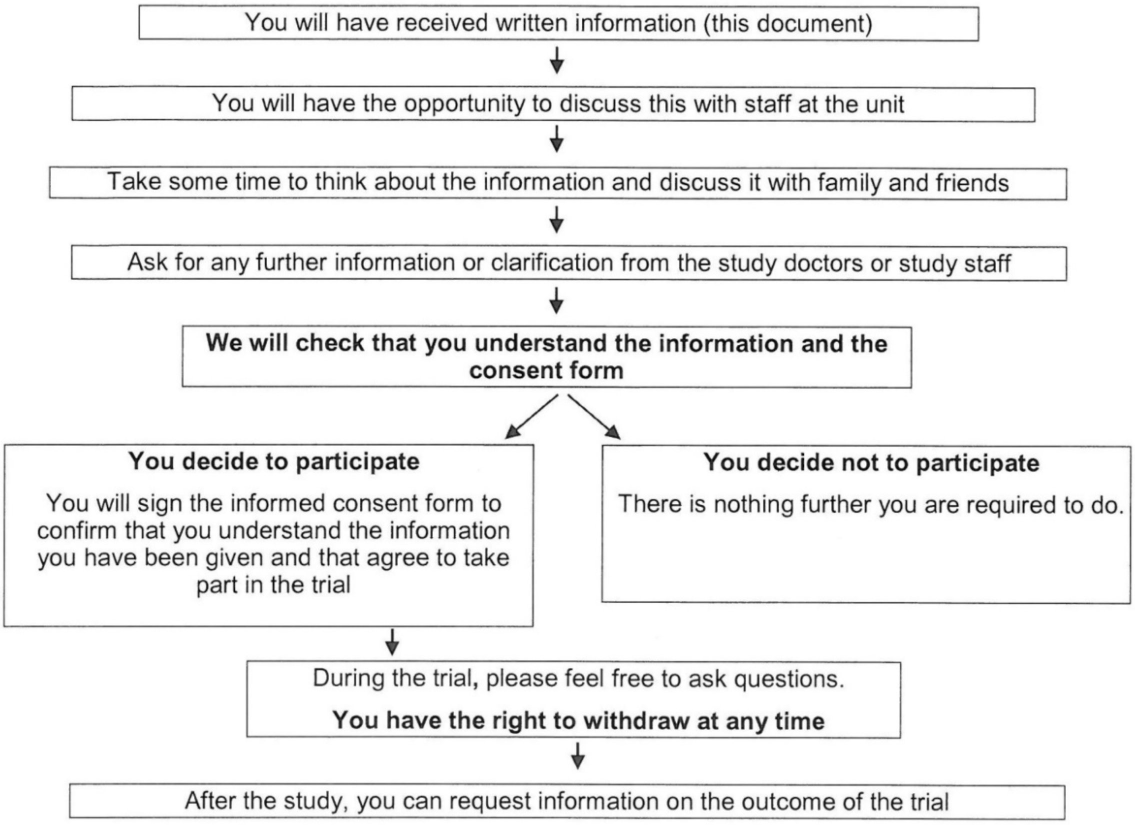

CONSENT FORM
(PART A)

**Short Title:**	A Study to Evaluate Single and Multiple Doses of TLC-2716 in Healthy Participants
**Protocol Number:**	2716-CL-101
**Principal Investigator:**	Prof Ed Gane

***Please let study staff know if you require an interpreter*.**

Declaration by participant:

- I have read or have had read to me in my first language, and I understand the Participant Information Sheet.
- I have been given sufficient time to consider whether or not to participate in this study.
- I have had the opportunity to use a legal representative, whanau/ family support or a friend to help me ask questions and understand the study.
- I am satisfied with the answers I have been given regarding the study and I have a copy of this consent form and information sheet.
- I understand that taking part in this study is voluntary (my choice) and that I may withdraw from the study at any time without this affecting my medical care.
- I consent to the research staff collecting and processing my information, including information about my health.
- If I decide to withdraw from the study, I agree that the information collected about me up to the point when I withdraw may continue to be processed.
- I understand that there may be risks associated with the treatment in the event of myself or my partner becoming pregnant. I undertake to inform my partner of the risks and to take responsibility for the prevention of pregnancy.
- I agree to my tissue samples being sent overseas, and I am aware that these samples will be disposed of using established guidelines for discarding biohazard waste.
- I agree to an approved auditor appointed by the New Zealand Health and Disability Ethic Committees, or any relevant regulatory authority or their approved representative reviewing my relevant medical records for the sole purpose of checking the accuracy of the information recorded for the study.
- I understand that my participation in this study is confidential and that no material, which could identify me personally, will be used in any reports on this study.
- I understand the compensation provisions in case of injury during the study.
- I know who to contact if I have any questions about the study in general.
- I understand my responsibilities as a study participant.
- I understand that I will receive a summary of the study results and if I do not want to receive this, I will inform site staff.
- I consent to my GP or current provider being informed about my participation in the study and of any significant abnormal results obtained during the study.

**Statement by Participant**	I hereby consent to take part in this study. I understand that I will receive a signed copy of this consent form for my records.<br><br>__________________________________ (full name)<br><br>__________________________________ (signature)<br><br>__ __ / __ __ __ / __ __ __ __ (Date DD/MMM/YYYY)

Statement by Consenter (Investigator/designee) I have discussed this study with the above-named participant. The participant appeared to fully understand the information provided about the study.

__________________________________ (full name)

__________________________________ (signature)

__________________________________ (project role)

__ __ / __ __ __ / __ __ __ __ (Date DD/MMM/YYYY)

PARTICIPANT INFORMATION SHEET AND CONSENT FORM
(Part B: Healthy Volunteer – Multiple Ascending)

**Short Title:**	A Study to Evaluate Single and Multiple Doses of TLC-2716 in Healthy Participants
**Protocol Number:**	2716-CL-101
**Sponsor:**	The Liver Company, Inc.<br>2671 Marshall Drive, Palo Alto, California United States of America
**Principal Investigator:**	Prof. Ed Gane
**Institution Address:**	Main Building: 3 Ferncroft Street, Grafton, Auckland, New Zealand<br>Outpatient Unit: 125 Grafton Road, Grafton, Auckland, New Zealand
**Phone Number:**	0800 STUDIES (0800 788 3437)
**Ethics Number:**	2022 FULL 12858

This is the first time that TLC-2716 will be studied in humans.
You will not get any health benefits from the drug used in this study; but there are risks of you having a drug reaction, injury, or illness.

You are invited to take part in a clinical research study. This study will test an investigational drug, named TLC-2716, that may potentially be used for the treatment of severe dyslipidaemias and non-alcoholic steatohepatitis (NASH). TLC-2716 is an investigational drug because it has not been approved by the New Zealand MedSafe or other drug regulatory authorities.

There are multiple parts to this study, and you are being asked to take part in Part B. This Participant Information Sheet will help you decide if you'd like to take part. It sets out why we are doing the study, what your participation would involve, what the benefits and risks to you might be, and what would happen after the study ends. We will go through this information with you and answer any questions you may have. We expect this will take about 30-60 minutes. You do not have to decide today whether or not you will participate in this study. Before you decide you may want to talk about the study with other people, such as family, whānau, friends, or healthcare providers. Feel free to do this.

Whether or not you take part is your choice. If you don't want to take part, you don't have to give a reason, and it won't affect the care you receive. If you do want to take part now, but change your mind later, you can pull out of the study at any time.

If you agree to take part in this study, you will be asked to sign and date the Consent Form on the last page of this document. You will be given a copy of both the Participant Information Sheet and the Consent Form to keep.

This document is **21** pages long, including the Consent Form. Please make sure you have read and understood all the pages.

1 WHY ARE WE DOING THE STUDY?

1.1 Purpose

TLC-2716 is being developed for the treatment of severe dyslipidaemias and NASH. Dyslipidaemia refers to unhealthy levels of one or more kinds of lipid (fat) in the blood. NASH is an advanced form of non-alcoholic fatty liver disease (NAFLD), which is caused by a build-up of fat in the liver. When NAFLD causes inflammation and damage in the liver, it progresses to NASH, which can then lead to liver cirrhosis (scarring of the liver), liver failure, and sometimes liver cancer. NASH is strongly linked to other conditions such as obesity, dyslipidaemia, high blood pressure, and type 2 diabetes. The prevalence of NASH is increasing worldwide, and the currently available treatment options are limited.

TLC-2716 works by inhibition (disruption the normal reaction) of a receptor in liver cells called liver X receptor (LXR) and is a key factor in regulation and metabolism of lipids, allowing the receptors to maintain normal or lower cholesterol and triglyceride levels throughout the body. It is hoped that, by maintaining or lowering cholesterol and triglyceride levels, TLC-2716 may be an effective treatment for severe dyslipidaemias or NASH.

This study will investigate the effects of single doses (Part A and C) or multiple doses (Parts B and C) of TLC-2716 in healthy participants. The purpose of Part B of this study is to:

- Evaluate how safe and well tolerated multiple doses of TLC-2716, in healthy participants.
- Measure levels of TLC-2716 in the blood over time, following multiple doses (pharmacokinetics/PK).
- Measure the body's response to multiple doses of TLC-2716 (pharmacodynamics/PD).

1.2 Study Design

Up to 150 healthy adults will take part in this study; 50 participants are to be enrolled in Part A, 50 in Part B, and 50 in Part C. <u>You are being asked to participate in Part B.</u>

This study requires an 18-night stay at the New Zealand Clinical Research (NZCR) research unit and 1 scheduled clinic visit.

This is a randomised, blinded, placebo-controlled study:

<u>Randomised</u> means that the study treatment you take (drug or placebo) will be assigned randomly (by chance).

<u>Blinded</u> means that neither you nor your study doctor will know whether you will be receiving TLC-2716 or placebo. In an emergency, the study doctor can find out what you are receiving.

On day -1 (the day prior to the first dose of TLC-2716/placebo), <u>all</u> participants in part B will receive a dose of placebo (a capsule that looks like TLC-2716 but contains no active medication), followed by blood tests to assess any changes in biological activity (biomarkers) in the absence of the active medication.

From day 1, every person in Part B of the study will receive one dose of TLC-2716 or placebo daily for 14 consecutive days. Each dose will be given by mouth, with a glass of water after a standardised meal.

Five dose groups (cohorts) are planned for Part B, respectively. If you are eligible for this study, you will be assigned to a cohort. The cohort you are assigned to will depend on when you join the study. You do not have a choice to which cohort you are assigned. You are only allowed to participate in one cohort. Details for the cohorts are as follows:

PART B		
Cohort	Planned Dose of TLC-2716 or Placebo	Frequency
6	0.5 mg	Once daily for 14 days via oral capsule (taken by mouth with water)
7	2 mg	
8	6 mg	
9	12 mg	
10	20 mg	

Each cohort will be comprised of 10 participants, where 8 participants will receive TLC-2716 and 2 participants will receive placebo. Whether you receive TLC-2716 or placebo will be assigned randomly (by chance). You will have a 4 out of 5 (80%) chance of getting TLC-2716 and 1 out of 5 (20%) chance of getting placebo.

Cohorts will be enrolled in order, where the next cohort testing a higher dose of TLC-2716 will be started if there are no safety concerns in the previous lower dose cohort. The Sponsor can decide to keep the dose the same, or select a lower dose for a cohort, based on the effects of TLC-2716 in previous cohorts. You will be told if any changes are made to the planned dose for your cohort.

Blood samples and other tests to measure study drug levels and effects on the body will be collected at specific time points during the study, your safety will be monitored, and any changes in your health will be recorded. Stool samples will be taken to measure pharmacodynamics and biomarkers of the study drug.

1.3 Nature and Sources of Funding of the Study

This research study is being sponsored globally and funded by The Liver Company, Inc. and conducted locally in New Zealand at NZCR.

By taking part in this research study, you agree that data generated from your assessments throughout the study, will be provided to the Sponsor. The knowledge gained from this data may lead to new discoveries, which would assist them in obtaining approval for a new drug and benefit the sponsor financially. There would be no financial benefit to you from these discoveries.

NZCR will receive a payment from The Liver Company, Inc. for undertaking this research study.

No member of the research team will receive a personal financial benefit from your involvement in this research study (other than their ordinary wages).

1.4 Approval by Ethics Committee

This study has been reviewed by an independent group of people called a Health and Disability Ethics Committee (HDEC). The ethical aspects of this research study have been approved by the Northern B Ethics Committee.

A description of this clinical study will be available on http://www.ClinicalTrials.gov. This web site will not include information that can identify you. At most, the web site will include a summary of the results. You can search this web site at any time.

2 WHAT WOULD YOUR PARTICIPATION INVOLVE?

Participation in this study will last up to approximately 8 weeks, which including a screening, treatment, and follow-up period. If you wish to participate in this study, you will be asked to sign this consent form before any study assessments can be performed.

The details of the study and tests performed are shown in the table below. During the screening period, assessments will be done to check whether the study is suitable for you. The day you have your first dose of TLC-2716 is called Day 1 and all other days are counted back or forward from this.

The results of the screening assessments will determine whether or not you can take part in the study. Entry into screening does not guarantee enrolment into the study. We may screen more participants than we need and so you may be asked to be a standby or a backup. This means you will be asked to come to the clinic and undergo the study tests and procedures until we have enrolled enough eligible participants for a cohort. You will then be discharged and where possible we will try to include you in a later cohort.

SCREENING	
**Screening Visit**<br><br>Day -28 to Day -3<br>Approx. 2 hours	Before any study procedures are performed, you will be asked to sign this informed consent form. Then the following assessments will be done:<br>• Review your health history<br>• Complete physical examination<br>• Height and weight<br>• Set of vital signs (blood pressure, heart rate, temperature)<br>• An ECG (to measure the electrical activity of your heart)<br>• Set of blood and urine samples for routine health tests<br>• Urine drug screen and alcohol breath test<br>• Viral infection blood tests (human immunodeficiency virus (HIV), hepatitis B virus (HBV), and hepatitis C virus (HCV))<br>• COVID-19 testing (and during the study if necessary)<br>• Pregnancy blood test (for women who are able to have children) or FSH test (to prove post-menopausal status)
A decision about whether you can take part will be made when all your screening results are available	
**TREATMENT PERIOD**	
**Inpatient Stay**<br><br>Day -2 to Day 17<br>*(18 nights inpatient total)*	**Day -2 (Admission):**<br>• Complete physical examination<br>• Weight<br>• Set of vital signs<br>• An ECG<br>• Urine drug screen and alcohol breath test<br>• Urine pregnancy test (for women who are able to have children)<br><br>**Day -1:**<br>• Dosed with a placebo capsule<br>• Set of vital signs<br>• Set of blood and urine samples for routine health tests<br>• Stool sample collection<br>• Blood samples to measure markers of biological activity and the effects of the drug on the body (biomarkers and PD)<br>**Day 1:**<br>• **Dose of TLC-2716/placebo**<br>• Physical examination (based on your symptoms)<br>• Set of vital signs<br>• Set of blood and urine samples for routine health tests<br>• An ECG<br>• Blood samples to measure level of study drug in your blood (pharmacokinetics/PK)<br>• Blood samples to measure biomarkers and PD<br><br>**Days 2, 3, 4, 5, 6, & 7:**<br>• **Dose of TLC-2716/placebo**<br>• Weight (Day 7)<br>• Physical examination (based on your symptoms)<br>• Set of vital signs (Days 2, 3, 5, and 7)<br>• Set of blood and urine samples for routine health tests (Days 2, 3, 5, and 7)<br>• An ECG (Days 3 and 7)<br>• Blood samples to measure PK (Days 3 and 7)<br>• Blood samples to measure biomarkers and PD (Day 7)

	**Days 8 & 9**<br>• **Dose of TLC-2716/placebo**<br>• Physical examination (based on your symptoms)<br><br>**Day 10:**<br>• **Dose of TLC-2716/placebo**<br>• Physical examination (based on your symptoms)<br>• Set of vital signs<br>• Set of blood and urine samples for routine health tests<br><br>**Days 11, 12, & 13:**<br>• **Dose of TLC-2716/placebo**<br>• Physical examination (based on your symptoms)<br><br>**Day 14:**<br>• **Dose of TLC-2716/placebo**<br>• Physical examination (based on your symptoms)<br>• Set of vital signs<br>• Set of blood and urine samples for routine health tests<br>• An ECG<br>• Blood samples to measure PK<br>• Blood samples to measure biomarkers and PD<br>• Stool sample<br><br>**Days 15 & 16:**<br>• Physical examination (based on your symptoms)<br>• Blood samples to measure PK<br><br>**Day 17 (Discharge):**<br>• Complete physical examination<br>• Weight<br>• Set of vital signs<br>• Set of blood and urine samples for routine health tests<br>• Blood samples to measure PK<br>• An ECG<br>• Pregnancy test (for women who are able to have children)<br><br>You will be discharged from the unit, following a study doctor's review.
**FOLLOW UP PERIOD**	
**Follow-Up Visit**<br>Day 28<br>Approx. 1 hour	• Complete physical examination<br>• Weight<br>• Set of vital signs<br>• An ECG<br>• Set of blood and urine samples for routine health tests<br>• Pregnancy test (for women who are able to have children)

You will be discharged from the study once your results have been checked by a study doctor.
END OF STUDY

2.1 Who Can Take Part in this Study?

To take part in this study you must:

- Be able to give consent and follow the study procedures.
- Be between the ages of 18 – 55 years, inclusive.
- Be in good health
- Have a body mass index (BMI) between 19.0 kg/m^2 – 35.0 kg/m^2.
- Be a non-smoker.

You cannot take part in this study if you:

- Are pregnant or breastfeeding.
- Have taken any prescription, over-the-counter medications, or herbal products (excluding contraceptives, ibuprofen, paracetamol or vitamins) within 28 days prior to dosing.
- Have used systemic steroids (e.g. prednisone or oral corticosteroids), immunosuppressive therapy, or chemotherapy within 3 months of screening, or are expecting to need these during the study.
- Have current alcohol or substance abuse.
- Have a history of a significant medical problem, mental health problem or severe allergy.
- Have a history of serious skin disease such as rashes/itching, food allergies, eczema, or psoriasis
- Have any history of significant heart problems, have a family history of long QT syndrome (a heart rhythm condition that can potentially cause fast, chaotic heartbeats).
- Have a history of fainting, heart palpitations, or unexplained dizziness.
- Have an implantable defibrillator or pacemaker.
- Have a have a history of liver disease.
- Have a history of severe peptic ulcer disease (open sores that develop on the inside lining of your stomach and the upper portion of your small intestine), or other gastric acid conditions such as gastric reflux.
- Have a history of medical or surgical treatment on the stomach or bowel (gallbladder removal is not exclusionary).
- Have received an investigational study drug in another clinical trial within 30 days, or potentially longer depending on the investigational drug, of your dose of TLC-2716 or placebo.
- Have donated blood within 56 days prior to admission

2.2 Study Instructions

It is important that you follow the instructions you are given and attend all scheduled clinic visits. It is also possible that the study doctor may schedule extra visits or tests for you, if considered necessary.

You will be given a Participant Identification Card if you participate in this study, stating the name of the study and the study doctor's contact information. This card should be carried with you at all times: you can contact the study doctor at any time or use it to inform any other doctor, dentist, or pharmacist that you are participating in this study.

At each visit, the study staff will ask you questions about your health and the medications you are currently taking. You should always report any changes in your health, unusual feelings, or symptoms to the study staff. It is also important that you do not start, stop, or make changes to any of the medications you are currently taking without first discussing this with your study doctor.

All of your meals will be provided during your inpatient stay. Meals play an important role in clinical trials due to their relationship with study drug metabolism (the way that the drug is processed and broken down by the

body). By signing the informed consent form, you are agreeing to be compliant with all meal requirements on this study.

Study Restrictions:

- You will not be able to smoke or use any nicotine-containing products from 90 days prior to admission (Day -1) until after your final follow up visit.
- You must not consume any alcohol from at least 72 hours prior to admission until after your final follow up visit.
- You must not consume any food or beverages containing grapefruit, grapefruit juice, Seville oranges or orange juice for at least four days prior to admission and until after your final follow up visit.
- You must be fasted (no food, only water) for at least 10 hours prior to your screening visit, and your follow up visit. Study staff will remind you prior to each visit that you need to be fasted.
- You are encouraged to avoid strenuous exercise, as well as saunas, steam baths, sunbathing, or prolonged UV exposure (e.g., tanning beds), from screening until after your final follow up visit.
- You must not donate blood from 56 days prior to admission or plasma from 7 days prior to admission, until 30 days following the dose of study drug.

The Investigator may request a random check of your bag(s) at admission for prohibited items (e.g., drinks or foods). Any prohibited items will be removed and returned to you on discharge from the unit.

3 WHAT ARE THE POSSIBLE BENEFITS AND RISKS TO YOU PARTICIPATING?

3.1 Benefits

You will not receive any direct medical benefits from taking part in this study. However, information learned from this study might help to develop better treatments for severe dyslipidaemias and NASH.

3.2 Reimbursement and Costs

All tests required to be done for this study will be paid for by The Liver Company, Inc. and there will be no cost for you to participate in this study.

You will be reimbursed the sum of $11,000 gross, following the final study visit. **We are required to deduct withholding tax at the applicable rate you have declared in the IR330C form**. You will be responsible for any other tax obligations (e.g., ACC). If you are GST registered, it may be possible for you to submit a GST invoice. Contact paymentforms@nzcr.co.nz if you would like to discuss this further.

The payment will be made after you complete the study, to cover your time and inconvenience. If you are receiving a benefit or allowance, your usual payments may be affected. Your tax statement will state that you were paid from your dosing day through to your final study visit.

You will be reimbursed for travel and parking for your study visits if you live in the metropolitan area. If you live outside this area, we will discuss your travel costs individually. Full reimbursement requires completion of all visits according to study requirements. If you are withdrawn from the study for medical reasons as a result of treatment given as part of this study, you will receive reimbursement in full.

If you leave the study of your own choice or are released from the study for non-medical reasons, you will receive a partial reimbursement (a pro-rata reimbursement) based on how many study days you completed. If you are invited to participate in the study as a Stand-by or Back-up participant and not enrolled in the study, you will receive reimbursement ($350 and $150, respectively) for your time and inconvenience. If you

completed the screening visit assessments but were not eligible to enrol in the study, you will not receive any reimbursement.

3.3 Possible Risks and Disadvantages?

Medical treatments may cause side effects. You may have none, some, or all of the side effects listed below. The side effects may feel mild, moderate, or severe. There may also be unknown side effects from taking TLC-2716 alone or with other drugs you may be taking. If you have any of these side effects, or are worried about them, talk with your study doctor. Your study doctor will also be looking out for side effects.

<u>What are the Risks or Side Effects of TLC-2716?</u>

This is the first time that TLC-2716 is being studied in humans. As such, there are unknown risks involved in receiving TLC-2716.

TLC-2716 was tested in animals with doses that are much higher than what will be tested in this study. In animal studies, the below side effects were observed, all of which were considered mild and reversible:

- Slight body weight loss
- Occasional soft and watery stools (diarrhea)

Allergic Reaction:
An immune reaction or allergic reaction is always possible with a drug you have not taken. Serious allergic reactions that can be life-threatening may occur. Some things that happen during any allergic reaction to any type of medication are:

- Rash
- Having a hard time breathing
- Wheezing when you breathe
- Sudden drop in blood pressure
- Swelling around the mouth, throat, or eyes
- Fast heart rate
- Sweating
- Itching
- Pain in your abdomen
- diarrhea
- Seizures

<u>What are the Risks or Side Effects of Study Procedures?</u>

Blood Sample Collection & Cannulas:
Risks include bruises, swelling with itching, and slight bleeding. The area may become inflamed. In rare cases, it may result in a blood clot, an infection or nerve damage. As needles can cause pain, you may feel light-headed or faint.

ECG Tests:
Sometimes the sticky pads used to attach the ECG leads can cause mild skin irritation (slight redness / itchiness).

Physical Examination:
During this examination you may be asked to remove some items of clothing so a full examination can be done. You can request a chaperone to be with you at the time of the physical examination, please ask one of our research nurses.

COVID-19 Vaccination

COVID-19 has been declared a pandemic by the World Health Organisation (WHO) and it is affecting nearly every community. During this study, you may not receive a COVID-19 vaccination or booster vaccination. Additionally, any vaccination must be completed at least 14 days prior to admission into the research unit.

Blood Sample Collection & Cannulas:

Risks include bruises, swelling with itching, and slight bleeding. The area may become inflamed. In rare cases, it may result in a blood clot, an infection or nerve damage. As needles can cause pain, you may feel light-headed or faint.

ECG Tests:

Sometimes the sticky pads used to attach the ECG leads can cause skin irritation (redness / itchiness).

Physical Examination:

During this examination you may be asked to remove some items of clothing so a full examination can be done. You can request a chaperone to be with you at the time of the physical examination, please ask one of our research nurses.

COVID-19

COVID-19 has been declared a pandemic by the World Health Organisation (WHO) and it is affecting nearly every community. During this study, you may not receive a COVID-19 vaccination or booster vaccination. Additionally, any vaccination must be completed at least 14 days prior to admission into the research unit.

3.4 Contraception

Reproductive Risks for Sexually Active Participants of Child-Bearing Potential

The effects of TLC-2716 in pregnancy and breastfeeding are unknown, but there is a risk it may cause birth defects or foetal deaths, and/or be passed on in breast milk. If you are pregnant or breastfeeding, you cannot take part in this study.

If you are sexually active and of child-bearing potential (able to become pregnant), it is very important that you do not become pregnant during this study. **You must use one of the methods of contraception listed below**, from at least screening until at least 30 days after your dose:

A woman of child-bearing potential is any pre-menopausal woman who may become pregnant. If you are unsure if this applies to you, please check with the study doctor before you start the study treatment.

A highly effective method (less than 1 pregnancy per 100 women using the method for one year) e.g.:

- Implant contraceptive (e.g., Jadelle®)
- Intra-uterine device (IUD) containing either copper or levonorgestrel (e.g., Mirena®)
- Male sterilisation (vasectomy)
- Female sterilisation (e.g., by bilateral tubal ligation ('clipping or tying tubes' or hysterectomy)

OR an effective method (5-10 pregnancies per 100 women using the method for one year) e.g.:

- Injectable contraceptive (e.g., Depo Provera)
- Oral Contraceptive Pill (combined hormonal pill or progestogen-only 'mini-pill')
- Vaginal contraceptive ring (e.g., NuvaRing®)

You / your partner must also use a barrier form of contraception if you are using an effective method of contraception from your screening through until 30 days after your dose. Barrier methods of contraception include:

- Male condoms
- Female condoms
- Female diaphragm ('cap')

Please note that barrier methods alone are not highly effective methods of birth control.

Total abstinence from heterosexual intercourse during the entire period of risk associated with the study drug (from screening until at least 30 days after your last dose) is considered an acceptable form of contraception if this is in line with your preferred and usual lifestyle.

If you are unsure which method of birth control you are using (or want to start using) and whether it is acceptable for this study, please ask the study doctor for more information.

You must also agree to not donate eggs, from dosing (Day 1) until at least 30 days following the dose of study drug.

If you do become pregnant during the study, you must tell the study doctor as soon as possible. If you do become pregnant you will be asked to sign a separate consent form, to allow the Sponsor to collect information about your pregnancy and the outcome of your pregnancy.

Reproductive Risks for Sexually Active Participants able to Father a Child

The effects of TLC-2716 if passed on through semen are unknown, but there is a risk it may cause birth defects or foetal deaths. **You are responsible for informing your sexual partner of these possible risks.**

If you are sexually active and have any partner who is of child-bearing potential (meaning a partner who may become pregnant) it is very important that you use contraception during this study. It is highly recommended that you and your partner use one of the contraception options listed above for participants of child-bearing potential, from at least dosing of the study drug through until at least 90 days after your dose.

You / your partner must also use a male condom method of contraception, from your dose of study drug through until 90 days after your dose.

Please note that barrier methods alone are not highly effective methods of contraception.

Total abstinence from heterosexual intercourse during the entire period of risk associated with the study drug (from dosing until at least 90 after your dose) is considered an acceptable form of contraception if this is in line with your preferred and usual lifestyle.

If a pregnancy occurs, you must report this to the study doctor as soon as possible. Your partner will be asked to give consent for her information and her infant's information to be collected for monitoring purposes.

You must also agree to not donate sperm, from dosing (Day 1) until 90 days following the dose of study drug.

4 WHAT WOULD HAPPEN IF YOU WERE INJURED IN THE STUDY?

As this research study is for the principal benefit of its commercial sponsor, The Liver Company, if you are injured as a result of taking part in this study you **won't** be eligible for compensation from ACC.

However, The Liver Company has satisfied the Northern B Health and Disability Ethics Committee that approved this study that it has up-to-date insurance for providing participants with compensation if they are injured as a result of taking part in this study.

New Zealand ethical guidelines for intervention studies require compensation for injury to be at least ACC equivalent. Compensation should be appropriate to the nature, severity and persistence of your injury and should be no less than would be awarded for similar injuries by New Zealand's ACC scheme.

Some sponsors voluntarily commit to providing compensation in accordance with guidelines that they have agreed between themselves, called the Medicines New Zealand Guidelines (Industry Guidelines). These are often referred to for information on compensation for commercial clinical trials. There are some important points to know about the Industry Guidelines:

- On their own they are not legally enforceable and may not provide ACC equivalent compensation.
- There are limitations on when compensation is available, for example compensation may be available for more serious, enduring injuries, and not for temporary pain or discomfort or less serious or curable complaints.
- Unlike ACC, the guidelines do not provide compensation on a no-fault basis:
- The Sponsor may not accept the compensation claim if:
    - Your injury was caused by the investigators, or;
    - There was a deviation from the proposed research plan, or;
    - Your injury was caused solely by you.

An initial decision whether to compensate you would be made the by the sponsor and/or its insurers.

If they decide not to compensate you, you may be able to take action through the Courts for compensation, but it could be expensive and lengthy, and you might require legal representation. You would need to be able to show that your injury was caused by participation in the trial.

You are strongly advised to read the Industry Guidelines and ask questions if you are unsure about what they mean for you.

If you have private health or life insurance, you may wish to check with your insurer that taking part in this study won't affect your cover.

5 WHAT WILL HAPPEN TO MY TEST SAMPLES?

Blood, urine and stool samples will be collected throughout the study. These samples will be used for various tests. Some of the samples will be used for regular routine blood counts and blood chemistry, and to monitor your general health.

All these routine samples will be sent to LabPlus for testing and destroyed after 3 months by internationally accepted means.

All other study samples (PK, PD and biomarkers) will be sent to central laboratories in Colorado Springs, Colorado, USA (Pyxant Labs), Singapore (LabCorp), Melbourne, Australia (360biolabs), and Adelaide, Australia (GNOMIX) for testing and destroyed after 3 years by internationally accepted means

The maximum amount of blood collected from each participant during the study will be up to 520 mL. For comparison, a standard blood donation at a blood collection centre, is about 470 mL. Samples collected by NZCR will be identified by your study number, year of birth, initials, and sex, to allow study doctors to quickly respond to any abnormal results. Before the results of these tests are sent to the Sponsor, your identifiable information will be removed and replaced with a code.

The proposed blood tests include a screening test for HIV and for viral Hepatitis B and C, as well as a COVID-19 test. Signing the consent form means that you agree to have this testing performed. It is important to understand that a positive screening test does not necessarily mean you have the disease. Should you receive a positive screening result for HIV or either Hepatitis B or C, then the study doctors will provide initial counselling and medical advice and will assist in arranging any follow up tests that you require. HIV, Hepatitis B and C, and COVID-19 are all 'notifiable diseases', which means that it is required by law to notify government health authorities of any new cases.

5.1 Are There Any Cultural Considerations?

You may hold beliefs about sacred and shared values about your tissue samples and/or data originating from this tissue. The cultural issues associated with sending your tissue samples and data overseas and/or storing your tissue and data should be discussed with your family/whanau as appropriate. If you need cultural support this can be provided. Please let us know and we will arrange this for you, or you can ring the Māori cultural support number at the bottom of this Participant Information Sheet and Consent Form. Cultural support is different to knowing more about the study. In these cases, we can arrange for an Investigator to come and talk to you and your whānau. Due to your samples being sent to countries outside of New Zealand, a karakia will not be able to be performed at the time of your sample disposal.

Personal and health information is a tāonga and will be treated accordingly. The following data sovereignty principles are in place to ensure that the data generated from this research is protected and may benefit Māori now and into the future. Clinical trials are often driven by Sponsor companies overseas and involve very few Māori as participants. However, NZCR will consider the principles of whakapapa, whanaungatanga, rangatiratanga, kotahitanga, manaakitanga and kaitiakitanga throughout the study.

6 WHAT ARE THE RIGHTS OF PARTICIPANTS IN THE STUDY?

6.1 Participation is Voluntary

Participation in any research study is voluntary. If you do not wish to take part, you do not have to. If you decide to take part and later change your mind, you are free to withdraw from the study at any stage. This will not affect your routine treatment/medical care which you may otherwise receive, or with NZCR.

If you do decide to take part, you will be given this Participant Information and Consent Form to sign and you will be given a copy to keep.

6.2 New Information

Sometimes during the course of a research study, new information becomes available about the treatment that is being studied. If this happens, your study doctor will tell you about it and discuss with you whether you want to continue in the research study. If you decide to withdraw, your study doctor will make arrangements for your regular health care to continue. If you decide to continue in the research study you may be asked to sign an updated consent form.

Also, on receiving new information, your study doctor might consider it to be in your best interests to withdraw you from the research study. If this happens, he/ she will explain the reasons and arrange for your regular health care to continue.

6.3 Privacy and Confidentiality and Right to Access Information Collected During the Study

What will Happen to my Information?

During this study, the study doctors, researchers, nurses and other NZCR staff will record information about you and your study participation. This includes the results of any study assessments. Your name, address, and phone number will be on the demographics page for the study so we can identify you correctly at visits and contact you. This information may be used to obtain health records (detailed below) but is not supplied to anyone overseas. If needed, information from your hospital records and your usual doctor (GP) may also be collected, and your GP may be notified about your participation in the study. You cannot take part in this study if you do not consent to the collection of this information.

Identifiable Information
Identifiable information is any data that could identify you (e.g., your name, date of birth, or address). The following groups may have access to your identifiable information:

- NZCR staff (to complete study assessments).
- Local laboratory staff, to process and report your screening and safety tests.
- Study monitors authorised by the Sponsor, to make sure the study is being run properly and that the data collected is accurate.
- Sponsor and its authorised representatives if you make a compensation claim as a result of study-related injury. Identifiable information is required to assess your claim.
- Sponsor, its authorised representatives, ethics committees, or government agencies from New Zealand or overseas, if the study and/or NZCR is audited. Audits are done to make sure that participants are protected, the study is run properly, and the data collected is correct.
- Your GP, if a study test gives an unexpected result that could be important in terms of your health. This allows appropriate follow-up to be arranged.
- The Medical Officer of Health, if you return a positive test for COVID-19, HIV, HBV, or HCV.

Rarely, it may be necessary for the study doctor to share your information with other people – for example, if there is a serious threat to public health or safety, or to the life or health of you or another person OR if the information is required in certain legal situations.

De-identified (Coded) Information
To make sure your personal information is kept confidential, information that identifies you will not be included in any study information sent to, or generated by, the Sponsor. Instead, you will be identified by a code. NZCR will keep a list linking your code with your name, so that you can be identified by your coded data if needed.

The following groups may have access to your coded information, which will be sent and stored overseas:

- The Sponsor, for the purposes of this study.
- People and companies working with or for the Sponsor, for the purposes of this study.
- Regulatory or other governmental agencies worldwide.

Anonymised Information
The Sponsor may remove the code from your de-identified information – this is called ‘anonymisation’. This makes it very difficult (but not impossible) to identify the information that belongs to you. The Sponsor may use this information for future research (see below).

Future Research Using Your Information
Your coded information may be used for future research related to the development of TLC-2716 and/or relating to severe dyslipidaemias, NASH, or similar diseases.

This future research may be conducted overseas. You will not be told when future research is undertaken using your information. Your information may be shared widely with other researchers or companies. Your information may also be added to information from other studies, to form much larger sets of data.

You will not get reports or other information about any / some research that is done using your information.

Your information may be used indefinitely for future research unless you withdraw your consent. However, it may be extremely difficult or impossible to access your information or withdraw consent for its use once your information has been shared for future research.

Security and Storage of Your Information
During the study, your information will be stored on paper forms at NZCR and electronically on secure servers. When the study has finished, paper forms will be transferred to a secure site and stored for at least 15 years, then destroyed. De-identified information in electronic form will remain on a secure platform and will be retained indefinitely. Storage will comply with local and/or international data security guidelines.

Risks
Although efforts will be made to protect your privacy, absolute confidentiality of your information cannot be guaranteed. Even with coded and anonymised information, there is no guarantee that you cannot be identified. While the risk is currently very small, the chance that someone might access and misuse your information (for example, by making it harder for you to get or keep a job or health insurance) might increase in the future as people find new ways of tracing information.

Your coded information is being sent overseas. Other countries may have lower levels of data protection than New Zealand. There may be no New Zealand representation on overseas organisations which make decisions about the use of your information. There is a risk that overseas researchers may work with information in a way that is not culturally appropriate for New Zealanders.

Rights to Access Your Information and Results
You have the right to request access to your information held by the research team. You also have the right to request that any information you disagree with is corrected.

Please ask if you would like to access your screening and safety tests during the study. You may access other study-specific information before the study is over, but this could result in you being withdrawn from the study to protect the study's scientific integrity.

If you have any questions about the collection and use of information about you, you should ask a study doctor.

7 WHAT WILL HAPPEN AFTER THE STUDY ENDS, OR IF I WANT TO PULL OUT?

7.1 If You Decide to Withdraw

You may withdraw your consent for the collection and use of your information at any time, by informing your study doctor. Please notify a member of the research team before you withdraw. This notice will allow that person or the research supervisor to discuss any health risks or special requirements linked to withdrawing, such as follow up visits.

If you withdraw your consent, your study participation will end, and the study team will stop collecting information from you. Information collected up until your withdrawal from the study will continue to be used and included in the study. This is to protect the quality of the study.

7.2 Why the Study Might be Unexpectedly Stopped

This research study may be stopped unexpectedly for a variety of reasons. These may include a decision by the study sponsor or unacceptable side effects observed in the study cohorts prior to or during your participation.

If you withdraw your consent, your study participation will end, and the study team will stop collecting information from you. Information collected up until your withdrawal from the study will continue to be used and included in the study. This is to protect the quality of the study.

7.3 Results

When the study ends the data must be analysed, so the results of the study may not be available until about a year after the research finishes. The study doctors and/or Sponsor may decide to discuss or publish the results of the study. This may include publication in journals, presentation at conferences or other professional forums. In any publication, information will be provided in such a way that you cannot be identified. Results of the study will be provided to you. If you do not wish to receive a summary of the study results when they become available, then please inform the NZCR staff.

NEW ZEALAND CLINICAL RESEARCH
Advancing global health

Appendix 1. Schedule of Assessments

Period	Screening	Treatment Period/Inpatient stay															Follow Up
**Study Day(s)**	**-28 to -1**	**-2**	**-1**	**1**	**2**	**3**	**4**	**5**	**6**	**7**	**8-9**	**10**	**11-13**	**14**	**15-16**	**17**	**28 EOS**
Admission to the unit		X															
Discharge from the unit																X	
Physical Exam	X	X	As required (symptom-directed)													X	X
Vital Signs	X	X	X	X	X	X		X		X		X		X		X	X
ECG	X	X		X						X				X	X	X	
BMI (Height & Weight)[1]	X	X								X						X	X
Drug and Alcohol Testing	X		X														
COVID-19 Testing	X		X														
Hepatitis/HIV Testing	X																
Dose Administration			X[2]	X	X	X	X	X	X	X	X	X	X	X			
Routine Blood & Urine Tests	X		X	X	X	X		X		X		X		X		X	X
PK Blood Tests				X		X				X				X	X	X	
Pregnancy Blood or Urine[3] Test	X	X														X	X
FSH test (post-menopausal women)	X																
PD & Biomarker Blood Tests			X	X						X				X			
Stool Sample			X											X			

BMI = body mass index; ECG = electrocardiogram; EOS = end of study; FSH = follicle stimulating hormone; PD = pharmacodynamics; PK = pharmacokinetics

[1] Height only taken at screening
[2] On day -1, all participants will receive a placebo dosing. Dosing with study drug/placebo where you are randomised and blinded to which arm you are in.
[3] Urine pregnancy test is done at admission, pregnancy blood tests are done at all other time points. These tests will only be done on women who are able to have children.

8 WHO DO I CONTACT FOR MORE INFORMATION OR IF I HAVE CONCERNS?

If you have any questions, concerns, or complaints about the study at any stage, you can contact:

Prof Ed Gane, Principal Investigator
Phone: (09) 373 3474 or 0800STUDIES (08007883437)
Email: waterfall@nzcr.co.nz

If you want to talk to someone who isn't involved with the study, you can contact an independent health and disability advocate on:

Phone:	0800 555 050
Fax:	0800 2 SUPPORT (0800 2787 7678)
Email:	advocacy@advocacy.org.nz
Website:	https://www.advocacy.org.nz/

Māori cultural support is available through:

The Office of the Chief Advisor Tikanga, He Kamaka Waiora, Waitematā and Auckland District Health Board:

Mobile:	021 0203 1167
Phone:	09 486 8320 ext 43204
Email:	hkwresearch@waitematadhb.govt.nz

You can also contact the health and disability ethics committee (HDEC) that approved this study on:

Phone:	0800 4 ETHIC (438 442)
Email:	hdecs@health.govt.nz

9 WHAT ABOUT ANY OTHER QUESTIONS I MAY HAVE?

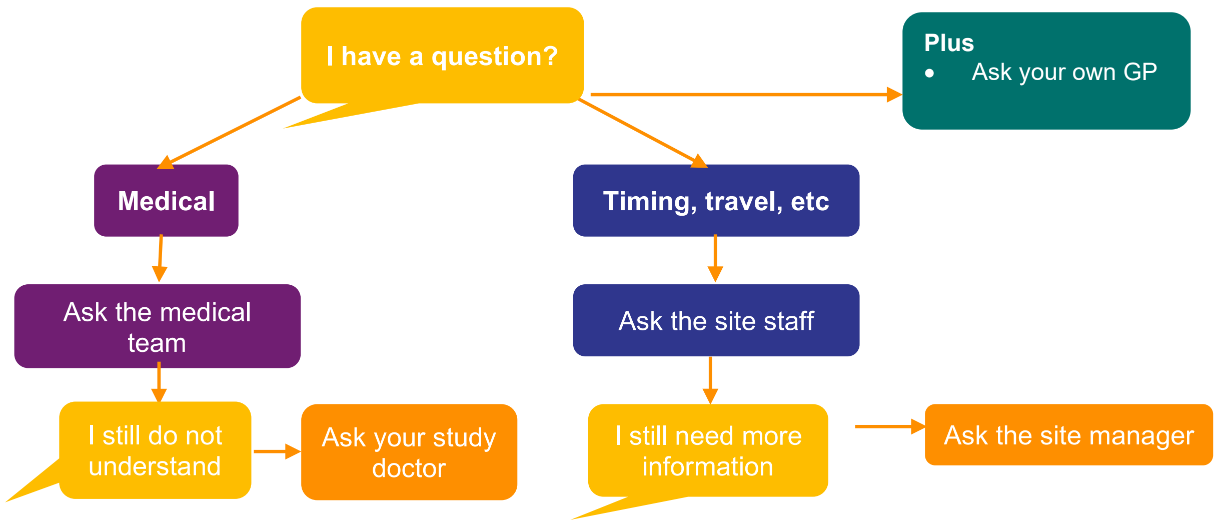

10 DO I HAVE TO DECIDE STRAIGHT AWAY?

No, you do not have to decide straight away. You should take some time to consider whether or not to participate in the study. We will be in touch in a week or so to discuss your decision**.** The following steps are useful in helping you reach a decision.

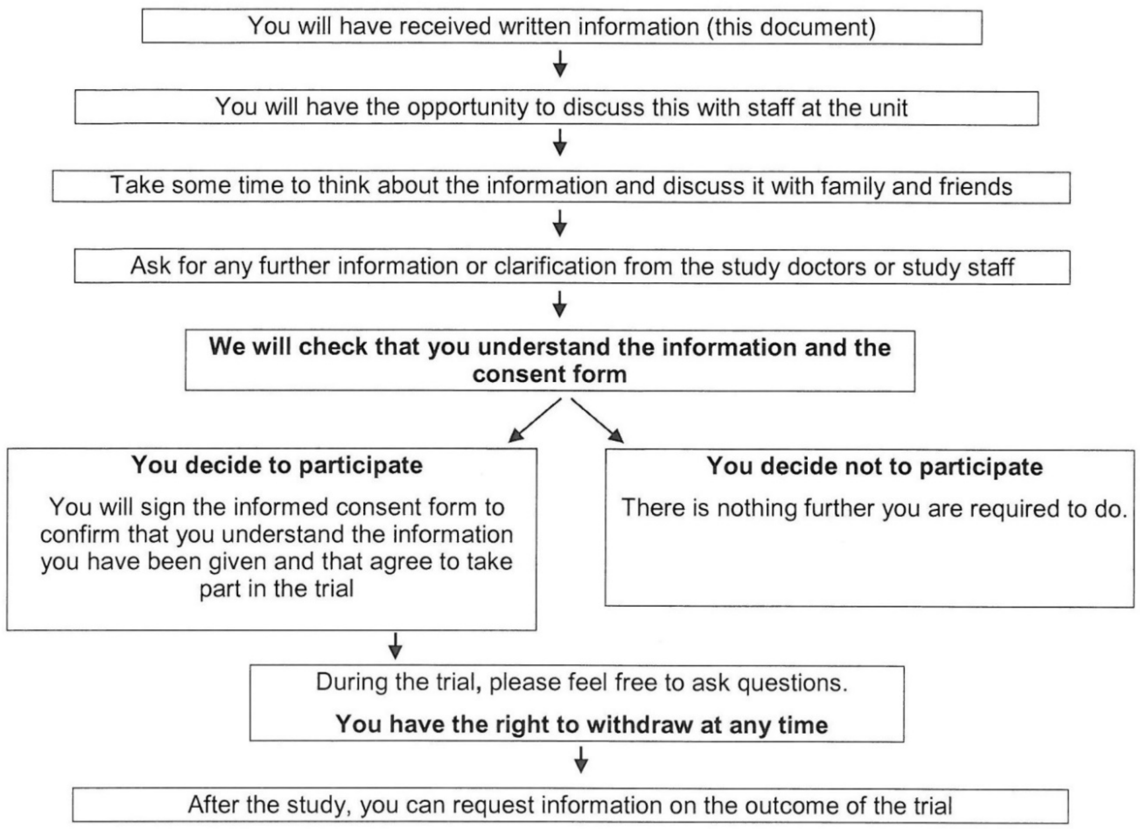

CONSENT FORM
(PART B)

**Short Title:**	A Study to Evaluate Single and Multiple Doses of TLC-2716 in Healthy Participants
**Protocol Number:**	2716-CL-101
**Principal Investigator:**	Prof Ed Gane

***Please let study staff know if you require an interpreter*.**

Declaration by participant:

- I have read or have had read to me in my first language, and I understand the Participant Information Sheet.
- I have been given sufficient time to consider whether or not to participate in this study.
- I have had the opportunity to use a legal representative, whanau/ family support or a friend to help me ask questions and understand the study.
- I am satisfied with the answers I have been given regarding the study and I have a copy of this consent form and information sheet.
- I understand that taking part in this study is voluntary (my choice) and that I may withdraw from the study at any time without this affecting my medical care.
- I consent to the research staff collecting and processing my information, including information about my health.
- If I decide to withdraw from the study, I agree that the information collected about me up to the point when I withdraw may continue to be processed.
- I understand that there may be risks associated with the treatment in the event of myself or my partner becoming pregnant. I undertake to inform my partner of the risks and to take responsibility for the prevention of pregnancy.
- I agree to my tissue samples being sent overseas, and I am aware that these samples will be disposed of using established guidelines for discarding biohazard waste.
- I agree to an approved auditor appointed by the New Zealand Health and Disability Ethic Committees, or any relevant regulatory authority or their approved representative reviewing my relevant medical records for the sole purpose of checking the accuracy of the information recorded for the study.
- I understand that my participation in this study is confidential and that no material, which could identify me personally, will be used in any reports on this study.
- I understand the compensation provisions in case of injury during the study.
- I know who to contact if I have any questions about the study in general.
- I understand my responsibilities as a study participant.
- I understand that I will receive a summary of the study results and if I do not want to receive this, I will inform site staff.
- I consent to my GP or current provider being informed about my participation in the study and of any significant abnormal results obtained during the study.

**Statement by Participant**	I hereby consent to take part in this study. I understand that I will receive a signed copy of this consent form for my records.<br><br>______________________________ (full name)<br><br>______________________________ (signature)<br><br>__ __ / __ __ __ / __ __ __ __ (Date DD/MMM/YYYY)

Statement by Consenter (Investigator/designee) I have discussed this study with the above-named participant. The participant appeared to fully understand the information provided about the study.

______________________________ (full name)

______________________________ (signature)

______________________________ (project role)

__ __ / __ __ __ / __ __ __ __ (Date DD/MMM/YYYY)

PARTICIPANT INFORMATION SHEET AND CONSENT FORM
(Part C: Adaptive Single and Multiple Ascending Dosing)

**Short Title:**	A Study to Evaluate Single and Multiple Doses of TLC-2716 in Healthy Participants
**Protocol Number:**	2716-CL-101
**Sponsor:**	The Liver Company, Inc.<br>2671 Marshall Drive, Palo Alto, California, United States of America
**Principal Investigator:**	Prof. Ed Gane
**Institution Address:**	Main Building: 3 Ferncroft Street, Grafton, Auckland, New Zealand<br>Outpatient Unit: 125 Grafton Road, Grafton, Auckland, New Zealand
**Phone Number:**	0800 STUDIES (0800 788 3437)
**Ethics Number:**	2022 FULL 12858

This is the first time that TLC-2716 will be studied in humans.
You will not get any health benefits from the drug used in this study; but there are risks of you having a drug reaction, injury, or illness.

You are invited to take part in a clinical research study. This study will test an investigational drug, named TLC-2716, that may potentially be used for the treatment of severe dyslipidaemias and non-alcoholic steatohepatitis (NASH). TLC-2716 is an investigational drug because it has not been approved by the New Zealand MedSafe or other regulatory authorities.

There are multiple parts to this study, and you are being asked to take part in Part C. This Participant Information Sheet will help you decide if you'd like to take part. It sets out why we are doing the study, what your participation would involve, what the benefits and risks to you might be, and what would happen after the study ends. We will go through this information with you and answer any questions you may have. We expect this will take about 30-60 minutes. You do not have to decide today whether or not you will participate in this study. Before you decide you may want to talk about the study with other people, such as family, whānau, friends, or healthcare providers. Feel free to do this.

Whether or not you take part is your choice. If you don't want to take part, you don't have to give a reason, and it won't affect the care you receive. If you do want to take part now, but change your mind later, you can pull out of the study at any time.

If you agree to take part in this study, you will be asked to sign and date the Consent Form on the last page of this document. You will be given a copy of both the Participant Information Sheet and the Consent Form to keep.

This document is **23** pages long, including the Consent Form. Please make sure you have read and understood all the pages.

1 WHY ARE WE DOING THE STUDY?

1.1 Purpose

TLC-2716 is being developed for the treatment of severe dyslipidaemias and NASH. Dyslipidaemia refers to unhealthy levels of one or more kinds of lipid (fat) in the blood. NASH is an advanced form of non-alcoholic fatty liver disease (NAFLD), which is caused by a build-up of fat in the liver. When NAFLD causes inflammation and damage in the liver, it progresses to NASH, which can then lead to liver cirrhosis (scarring of the liver), liver failure, and sometimes liver cancer. NASH is strongly linked to other conditions such as obesity, dyslipidaemia, high blood pressure, and type 2 diabetes. The prevalence of NASH is increasing worldwide, and the currently available treatment options are limited.

TLC-2716 works by inhibition (disruption the normal reaction) of a receptor in liver cells called liver X receptor (LXR) and is a key factor in regulation and metabolism of lipids, allowing the receptors to maintain normal or lower cholesterol and triglyceride levels throughout the body. It is hoped that, by maintaining or lowering cholesterol and triglyceride levels, TLC-2716 may be an effective treatment for severe dyslipidaemias or NASH.

This study will investigate the effects of single doses (Parts A and C) or multiple doses (Parts B and C) of TLC-2716 in healthy participants. The purpose of Part C of this study is to:

- Evaluate how safe and well tolerated TLC-2716 is, in healthy participants.
- Measure levels of TLC-2716 in the blood over time, following a single or multiple dose(s) of TLC-2716 (pharmacokinetics/PK).
- Measure the body's response to single or multiple dose(s) of TLC-2716, in either fed or fasted states (pharmacodynamics/PD).

1.2 Study Design

Up to 150 healthy adults will take part in this study; 50 participants will be enrolled in Part A, 50 in Part B, and 50 in Part C. <u>You are being asked to participate in Part C.</u>

Part C of this study is an **adaptive design.** Adaptive means that depending on what group (cohort) you enrol in, you may receive a single dose or multiple doses (taken either once or twice a day), under either fed or fasted conditions. The dose level (the amount of active drug you receive) may differ as well. A decision will be made on what these cohorts will be after we receive some results from Part A and Part B.

- **Single Ascending Dosing (SAD):** If you are in a cohort that receives a single dose you will require to stay 4 consecutive nights in the research clinic and come in for one scheduled visit.

- **Multiple Ascending Dosing (MAD):** If you are in a cohort that receives multiple doses you will require to stay 18 consecutive nights in the research clinic and come in for one scheduled visit.

<u>You will be told what cohort you will be going into.</u>

This is a randomised, blinded, placebo-controlled study:

<u>Randomised</u> means that the study treatment you take (drug or placebo) will be assigned randomly (by chance).

<u>Blinded</u> means that neither you nor your study doctor will know whether you will be receiving TLC-2716 or placebo. In an emergency, the study doctor can find out what you are receiving.

Every person in the study will receive either a single dose (SAD) **OR** 14 doses consecutive doses (MAD) of TLC-2716 or placebo (a capsule that looks like TLC-2716 but contains no active medication). Each dose will be given by mouth, with a glass of water. A decision will be made closer to the time around whether you will have a meal prior to dosing (non-fasting), or whether you will have the medication on an empty stomach (fasting).

Five cohorts (Cohorts 11-15) are planned for the study which will include 10 participants each. As this study is adaptive, the cohort design will be decided closer to the time. The cohort you are assigned to will depend on when you join the study. Details for the SAD and MAD design is as follows:

PART C		
**SAD or MAD Cohorts 11-15**	**Planned Dose of TLC-2716 or Placebo**	**Frequency**
SAD	≤ 50 mg total daily dose*	1 on day 1 via oral capsule (taken by mouth with water)
MAD	≤ 50 mg total daily dose*	1 or 2 doses **daily for 14 days** via oral capsule (taken by mouth with water)

** Doses for Part C will be determined based on data from previous cohorts. Your total daily dose of TLC-2716 or placebo will not exceed 50 mg and may be given either with a meal or on an empty stomach.*

SAD cohorts only: In each SAD cohort, two participants will be dosed first (one will receive TLC-2716 and the other will receive placebo). These participants are called 'sentinels. The sentinel participants will be monitored for approximately 2 days, and if there are no safety concerns, the next 8 participants will be dosed (7 participants will receive TLC-2716 and 1 will receive placebo).

MAD cohorts only: On Day -1 (the day prior to the first dose of TLC-2716/placebo), all participants will receive a dose of placebo, followed by blood tests to assess any changes in biological activity (biomarkers) in the absence of the active medication. There may be a MAD cohort that receives a total daily dose of TLC-2716 that is greater than what has been evaluated in previous cohorts. For this MAD cohort, two sentinel participants will be dosed first (one will receive TLC-2716 and the other will receive placebo). The sentinel participants will be monitored for approximately 2 days, and if there are no safety concerns, the next 8 participants will be dosed (7 participants will receive TLC-2716 and 1 will receive placebo).

Whether you receive TLC-2716 or placebo will be assigned randomly (by chance). From Day 1, 8 participants will receive TLC-2716 and 2 will receive placebo in each cohort. You will be told what cohort you will go into, whether its one or two doses per day, and whether you will be fasted or non-fasted.

Cohorts will be enrolled in order. You will be told which cohort you will be in. You will also be told if any changes are made to the planned dose for your cohort.

Blood samples and other tests to measure study drug levels and effects on the body will be collected at specific time points during the study, your safety will be monitored, and any changes in your health will be recorded.

1.3 Nature and Sources of Funding of the Study

This research study is being sponsored globally and funded by The Liver Company, Inc. and conducted locally in New Zealand at NZCR.

By taking part in this research study, you agree that data generated from your assessments throughout the study, will be provided to the Sponsor. The knowledge gained from this data may lead to new discoveries, which would assist them in obtaining approval for a new drug and benefit the sponsor financially. There would be no financial benefit to you from these discoveries.

NZCR will receive a payment from The Liver Company, Inc. for undertaking this research study.

No member of the research team will receive a personal financial benefit from your involvement in this research study (other than their ordinary wages).

1.4 Approval by Ethics Committee.

This study has been reviewed by an independent group of people called a Health and Disability Ethics Committee (HDEC). The ethical aspects of this research study have been approved by the **Northern B** Ethics Committee.

A description of this clinical study will be available on http://www.ClinicalTrials.gov. This web site will not include information that can identify you. At most, the web site will include a summary of the results. You can search this web site at any time.

2 WHAT WOULD YOUR PARTICIPATION INVOLVE?

Participation in this study will last up to approximately 6 weeks (SAD cohorts) or 8 weeks (MAD cohorts), which includes the screening, treatment, and follow-up periods. If you wish to participate in this study, you will be asked to sign this consent form before any study assessments can be performed.

The details of the study and tests performed are shown in the table below. During the screening period, assessments will be done to check that you meet the requirements to be a participant in the study. The day you have your first dose of TLC-2716 is called Day 1 and all other study days are counted backward or forward from this day.

The results of the screening assessments will determine whether or not you can take part in the study. Entry into screening does not guarantee enrolment into the study. We may screen more participants than we need and so you may be asked to be a standby or a backup. This means you will be asked to come to the clinic and undergo the study tests and procedures until we have enrolled enough eligible participants for a cohort. You will then be discharged and where possible we will try to include you in a later cohort.

SINGLE ASCENDING DOSING (SAD) SCHEDULE	
**SCREENING**	
**Screening Visit**<br><br>Day -28 to Day -2<br>Approx. 2 hours	Before any study procedures are performed, you will be asked to sign this informed consent form. Then the following assessments will be done:<br>• Review your health history<br>• Complete physical examination<br>• Height and weight<br>• Set of vital signs (blood pressure, heart rate, temperature)<br>• An ECG (to measure the electrical activity of your heart)<br>• Set of blood and urine samples for routine health tests<br>• Urine drug screen and alcohol breath test<br>• Viral infection blood tests (human immunodeficiency virus [HIV], hepatitis B virus [HBV], and hepatitis C virus [HCV])<br>• COVID-19 testing (and during the study if necessary)<br>• Pregnancy blood test (for women who are able to have children)
A decision about whether you can take part will be made when all your screening results are available	
**TREATMENT PERIOD**	
**Inpatient Stay**<br><br>Day -1 to Day 4	**Day -1 (Admission):**<br>• Complete physical examination<br>• Weight<br>• Set of vital signs<br>• An ECG<br>• Set of blood and urine samples for routine health tests<br>• Urine drug screen and alcohol breath test<br>• Urine pregnancy test (for women who are able to have children)<br>• -<br>**Day 1:**<br>• **Dose of either one OR two doses of TLC-2716 or Placebo**<br>• Physical exam (based on your symptoms)<br>• Set of vital signs<br>• Set of blood and urine samples for routine health tests<br>• An ECG<br>• Blood samples to measure level of study drug in your blood (PK)<br>• Blood samples to measure biomarkers and PD<br><br>**Days 2 & 3:**<br>• Physical exam (based on your symptoms)<br>• Set of vital signs<br>• Set of safety blood and urine samples<br>• An ECG (Day 2 only)<br>• Blood samples to measure PK<br>**Day 4 (Discharge):**<br>• Complete physical examination<br>• Weight<br>• Set of vital signs<br>• Physical examination<br>• Set of blood and urine samples<br>• Pregnancy blood test (for women who are able to have children)<br>• An ECG<br>• Blood samples to measure PK<br>You will be discharged from the unit, following a study doctor's review.
**FOLLOW UP PERIOD**	

**Follow-up Visit**<br>Day 15<br>Approx. 1 hour	• Complete physical examination<br>• Weight<br>• Set of vital signs<br>• An ECG<br>• Set of blood and urine samples for routine health tests<br>• Pregnancy blood test (for women who are able to have children)
**You will be discharged from the study once your results have been checked by a study doctor.**<br>**END OF STUDY**	

MULTIPLE ASCENDING DOSING (MAD) SCHEDULE	
**SCREENING**	
**Screening Visit**<br><br>Day -28 to Day -3<br>Approx. 2 hours	Before any study procedures are performed, you will be asked to sign this informed consent form. Then the following assessments will be done:<br>• Review your health history<br>• Complete physical examination<br>• Height and weight<br>• Set of vital signs (blood pressure, heart rate, temperature)<br>• An ECG (to measure the electrical activity of your heart)<br>• Set of blood and urine samples for routine health tests<br>• Urine drug screen and alcohol breath test<br>• Testing for HIV, Hepatitis B and C<br>• COVID-19 testing (and during the study if necessary)<br>• Pregnancy blood test (for women who are able to have children)
A decision about whether you can take part will be made when all your screening results are available	
**TREATMENT PERIOD**	
**Inpatient Stay**<br><br>Day -2 to Day 17<br>*(18 nights inpatient total)*	**Day -2 (Admission):**<br>• Complete physical examination<br>• Weight<br>• Set of vital signs<br>• An ECG<br>• Urine drug screen and alcohol breath test<br>• Urine pregnancy test (for women who are able to have children)<br><br>**Day -1:**<br>• Dosed with a placebo capsule<br>• Set of vital signs<br>• Set of blood and urine samples for routine health tests<br>• Stool sample collection<br>• Blood samples to measure markers of biological activity and the effects of the drug on the body (biomarkers and PD)<br>**Day 1:**<br>• **One OR two doses of TLC-2716/placebo**<br>• Physical examination (based on your symptoms)<br>• Set of vital signs<br>• Set of blood and urine samples for routine health tests<br>• An ECG<br>• Blood samples to measure level of study drug in your blood (PK)<br>• Blood samples to measure biomarkers and PD<br><br>**Days 2, 3, 4, 5, 6, & 7:**<br>• **One OR two doses of TLC-2716/placebo**<br>• Weight (Day 7)<br>• Physical examination (based on your symptoms)<br>• Set of vital signs (Days 2, 3, 5, and 7)<br>• Set of blood and urine samples for routine health tests (Days 2, 3, 5, and 7)<br>• An ECG (Days 3 and 7)<br>• Blood samples to measure PK (Days 3 and 7)<br>• Blood samples to measure biomarkers and PD (Day 7)<br><br>**Days 8 & 9**

	• **One OR two doses of TLC-2716/placebo**<br>• Physical examination (based on your symptoms)<br><br>**Day 10:**<br>• **One OR two doses of TLC-2716/placebo**<br>• Physical examination (based on your symptoms)<br>• Set of vital signs<br>• Set of blood and urine samples for routine health tests<br><br>**Days 11, 12, & 13:**<br>• **One OR two doses of TLC-2716/placebo**<br>• Physical examination (based on your symptoms)<br><br>**Day 14:**<br>• **One OR two doses of TLC-2716/placebo**<br>• Physical examination (based on your symptoms)<br>• Set of vital signs<br>• Set of blood and urine samples for routine health tests<br>• An ECG<br>• Blood samples to measure PK<br>• Blood samples to measure biomarkers and PD<br>• Stool sample<br><br>**Days 15 & 16:**<br>• Physical examination (based on your symptoms)<br>• Blood samples to measure PK<br><br>**Day 17 (Discharge):**<br>• Complete physical examination<br>• Weight<br>• Set of vital signs<br>• Set of blood and urine samples for routine health tests<br>• Blood samples to measure PK<br>• An ECG<br>• Pregnancy test (for women who are able to have children)<br><br>**You will be discharged from the unit, following a study doctor's review.**
**FOLLOW UP PERIOD**	
**Study Exit Visit**<br>Day 28<br>Approx. 1 hour	• Complete physical examination<br>• Weight<br>• Set of vital signs<br>• An ECG<br>• Set of blood and urine samples for routine health tests<br>• Pregnancy test (for women who are able to have children)

You will be discharged from the study once your results have been checked by a study doctor.
END OF STUDY

2.1 Who Can Take Part in this Study?

To take part in this study you must:

- Be able to give consent and follow the study procedures.
- Be between the ages of 18 – 55 years, inclusive.
- Be in good health
- Have a body mass index (BMI) between 19.0 kg/m^2 – 35.0 kg/m^2.
- Be a non-smoker.

You cannot take part in this study if you:

- Are pregnant or breastfeeding.
- Have taken any prescription, over-the-counter medications, or herbal products (excluding contraceptives, ibuprofen, paracetamol or vitamins) within 28 days prior to dosing.
- Have used systemic steroids (e.g. prednisone or oral corticosteroids), immunosuppressive therapy, or chemotherapy within 3 months of screening, or are expecting to need these during the study.
- Have current alcohol or substance abuse.
- Have a history of a significant medical problem, mental health problem or severe allergy.
- Have a history of serious skin disease such as rashes/itching, food allergies, eczema, or psoriasis
- Have any history of significant heart problems, have a family history of long QT syndrome (a heart rhythm condition that can potentially cause fast, chaotic heartbeats).
- Have a history of fainting, heart palpitations, or unexplained dizziness.
- Have an implantable defibrillator or pacemaker.
- Have a have a history of liver disease.
- Have a history of severe peptic ulcer disease (open sores that develop on the inside lining of your stomach and the upper portion of your small intestine), or other gastric acid conditions such as gastric reflux.
- Have a history of medical or surgical treatment on the stomach or bowel (gallbladder removal is not exclusionary).
- Have received an investigational study drug in another clinical trial within 30 days, or potentially longer depending on the investigational drug, of your dose of TLC-2716 or placebo.
- Have donated blood within 56 days prior to admission

2.2 Study Instructions

It is important that you follow the instructions you are given and attend all scheduled clinic visits. It is also possible that the study doctor may schedule extra visits or tests for you, if considered necessary.

You will be given a Participant Identification Card if you participate in this study, stating the name of the study and the study doctor's contact information. This card should be carried with you at all times: you can contact the study doctor at any time or use it to inform any other doctor, dentist, or pharmacist that you are participating in this study.

At each visit, the study staff will ask you questions about your health and the medications you are currently taking. You should always report any changes in your health, unusual feelings, or symptoms to the study staff. It is also important that you do not start, stop, or make changes to any of the medications you are currently taking without first discussing this with your study doctor.

All of your meals will be provided during your inpatient stay. Meals play an important role in clinical trials due to their relationship with study drug metabolism (the way that the drug is processed and broken down by the

body). By signing the informed consent form, you are agreeing to be compliant with all meal requirements on this study.

Restrictions:

- You will not be able to smoke or use any nicotine-containing products from 90 days prior to admission (Day -1) until after your final follow up visit.
- You must not consume any alcohol from at least 72 hours prior to admission until after your final follow up visit.
- You must not consume any food or beverages containing grapefruit, grapefruit juice, Seville oranges or orange juice for at least four days prior to admission and until after your final follow up visit.
- You must be fasted (no food, only water) for at least 10 hours prior to your screening visit, admission (SAD cohorts only) and your follow up visit. Study staff will remind you prior to each visit that you need to be fasted.
- You are encouraged to avoid strenuous exercise, as well as saunas, steam baths, sunbathing, or prolonged UV exposure (e.g., tanning beds), from screening until after your final follow up visit.
- You must not donate blood from 56 days prior to admission or plasma from 7 days prior to admission, until 30 days following the dose of study drug.

The Investigator may request a random check of your bag(s) at admission for prohibited items (e.g., drinks or foods). Any prohibited items will be removed and returned to you on discharge from the unit.

3 WHAT ARE THE POSSIBLE BENEFITS AND RISKS TO YOU PARTICIPATING?

3.1 Benefits

You will not receive any direct medical benefits from taking part in this study. However, information learned from this study might help to develop better treatments for severe dyslipidaemias and NASH.

3.2 Reimbursement and Costs

All tests required to be done for this study will be paid for by The Liver Company, Inc. and there will be no cost for you to participate in this study.

You will be reimbursed the sum of $3,500 gross (if you are in a group that stays 4-nights/SAD), or $11,000 gross (if you are in a group that stays 18-nights/MAD) following the final follow up visit**. We are required to deduct withholding tax at the applicable rate you have declared in the IR330C form**. You will be responsible for any other tax obligations (e.g., ACC). If you are GST registered, it may be possible for you to submit a GST invoice. Contact paymentforms@nzcr.co.nz if you would like to discuss this further.

The payment will be made after you complete the study, to cover your time and inconvenience. If you are receiving a benefit or allowance, your usual payments may be affected. Your tax statement will state that you were paid from your dosing day through to your final study visit.

You will be reimbursed for travel and parking for your study visits if you live in the metropolitan area. If you live outside this area, we will discuss your travel costs individually. Full reimbursement requires completion of all visits according to study requirements. If you are withdrawn from the study for medical reasons as a result of treatment given as part of this study, you will receive reimbursement in full.

If you leave the study of your own choice or are released from the study for non-medical reasons, you will receive a partial reimbursement (a pro-rata reimbursement) based on how many study days you completed. If you are invited to participate in the study as a Stand-by or Back-up participant and not enrolled in the study,

you will receive reimbursement ($350 and $150, respectively) for your time and inconvenience. If you completed the screening visit assessments but were not eligible to enrol in the study, you will not receive any reimbursement.

3.3 Possible Risks and Disadvantages?

Medical treatments may cause side effects. You may have none, some, or all of the side effects listed below. The side effects may feel mild, moderate, or severe. There may also be unknown side effects from taking TLC-2716 alone or with other drugs you may be taking. If you have any of these side effects, or are worried about them, talk with your study doctor. Your study doctor will also be looking out for side effects.

What are the Risks or Side Effects of TLC-2716?

This is the first time that TLC-2716 is being studied in humans. As such, there are unknown risks involved in receiving TLC-2716.

TLC-2716 was tested in animals with doses that are much higher than what will be tested in this study. In animal studies, the below side effects were observed, all of which were considered mild and reversible:

- Slight body weight loss
- Occasional soft and watery stools (diarrhea)

Allergic Reaction:
An immune reaction or allergic reaction is always possible with a drug you have not taken. Serious allergic reactions that can be life-threatening may occur. Some things that happen during any allergic reaction to any type of medication are:

- Rash
- Having a hard time breathing
- Wheezing when you breathe
- Sudden drop in blood pressure
- Swelling around the mouth, throat, or eyes
- Fast heart rate
- Sweating
- Itching
- Pain in your abdomen
- diarrhea
- Seizures

What are the Risks or Side Effects of Study Procedures?

Blood Sample Collection & Cannulas:
Risks include bruises, swelling with itching, and slight bleeding. The area may become inflamed. In rare cases, it may result in a blood clot, an infection or nerve damage. As needles can cause pain, you may feel light-headed or faint.
ECG Tests:
Sometimes the sticky pads used to attach the ECG leads can cause mild skin irritation (slight redness / itchiness).
Physical Examination:
During this examination you may be asked to remove some items of clothing so a full examination can be done. You can request a chaperone to be with you at the time of the physical examination, please ask one of our research nurses.

COVID-19

COVID-19 has been declared a pandemic by the World Health Organisation (WHO) and it is affecting nearly every community. During this study, you may not receive a COVID-19 vaccination or booster vaccination. Additionally, any vaccination must be completed at least 14 days prior to admission into the research unit.

3.4 Contraception

Reproductive Risks for Sexually Active Participants of Child-Bearing Potential

The effects of TLC-2716 in pregnancy and breastfeeding are unknown, but there is a risk it may cause birth defects or foetal deaths, and/or be passed on in breast milk. If you are pregnant or breastfeeding, you cannot take part in this study.

If you are sexually active and of child-bearing potential (able to become pregnant), it is very important that you do not become pregnant during this study. **You must use one of the methods of contraception listed below**, from at least screening until at least 30 days after your dose:

A woman of child-bearing potential is any pre-menopausal woman who may become pregnant. If you are unsure if this applies to you, please check with the study doctor before you start the study treatment.

A highly effective method (less than 1 pregnancy per 100 women using the method for one year) e.g.:

- Implant contraceptive (e.g., Jadelle®)
- Intra-uterine device (IUD) containing either copper or levonorgestrel (e.g., Mirena®)
- Male sterilisation (vasectomy)
- Female sterilisation (e.g., by bilateral tubal ligation (‘clipping or tying tubes’ or hysterectomy)

OR an effective method (5-10 pregnancies per 100 women using the method for one year) e.g.:

- Injectable contraceptive (e.g., Depo Provera)
- Oral Contraceptive Pill (combined hormonal pill or progestogen-only ‘mini-pill’)
- Vaginal contraceptive ring (e.g., NuvaRing®)

You / your partner must also use a barrier form of contraception if you are using an effective method of contraception, from your screening through until 30 days after your dose. Barrier methods of contraception include:

- Male condoms
- Female condoms
- Female diaphragm (‘cap’)

Please note that barrier methods alone are not highly effective methods of birth control.

Total abstinence from heterosexual intercourse during the entire period of risk associated with the study drug (from screening until at least 30 days after your last dose) is considered an acceptable form of contraception if this is in line with your preferred and usual lifestyle.

If you are unsure which method of birth control you are using (or want to start using) and whether it is acceptable for this study, please ask the study doctor for more information.

You must also agree to not donate eggs, from dosing (Day 1) until at least 30 days following the dose of study drug.

If you do become pregnant during the study, you must tell the study doctor as soon as possible. If you do become pregnant you will be asked to sign a separate consent form, to allow the Sponsor to collect information about your pregnancy and the outcome of your pregnancy.

Reproductive Risks for Sexually Active Participants able to Father a Child

The effects of TLC-2716 if passed on through semen are unknown, but there is a risk it may cause birth defects or foetal deaths. **You are responsible for informing your sexual partner of these possible risks.**

If you are sexually active and have any partner who is of child-bearing potential (meaning a partner who may become pregnant) it is very important that you use contraception during this study. It is highly recommended that you and your partner use one of the contraception options listed above for participants of child-bearing potential, from at least dosing of the study drug through until at least 90 days after your dose.

You / your partner must also use a male condom method of contraception, from your dose of study drug through until 90 days after your dose.

Please note that barrier methods alone are not highly effective methods of contraception.

Total abstinence from heterosexual intercourse during the entire period of risk associated with the study drug (from dosing until at least 90 after your dose) is considered an acceptable form of contraception if this is in line with your preferred and usual lifestyle.

If a pregnancy occurs, you must report this to the study doctor as soon as possible. Your partner will be asked to give consent for her information and her infant's information to be collected for monitoring purposes.

You must also agree to not donate sperm, from dosing (Day 1) until 90 days following the dose of study drug.

4 WHAT WOULD HAPPEN IF YOU WERE INJURED IN THE STUDY?

As this research study is for the principal benefit of its commercial sponsor, The Liver Company, Ltd, if you are injured as a result of taking part in this study you **won't** be eligible for compensation from ACC.

However, The Liver Company has satisfied the Northern B Health and Disability Ethics Committee that approved this study that it has up-to-date insurance for providing participants with compensation if they are injured as a result of taking part in this study.

New Zealand ethical guidelines for intervention studies require compensation for injury to be at least ACC equivalent. Compensation should be appropriate to the nature, severity and persistence of your injury and should be no less than would be awarded for similar injuries by New Zealand's ACC scheme.

Some sponsors voluntarily commit to providing compensation in accordance with guidelines that they have agreed between themselves, called the Medicines New Zealand Guidelines (Industry Guidelines). These are often referred to for information on compensation for commercial clinical trials. There are some important points to know about the Industry Guidelines:

- On their own they are not legally enforceable and may not provide ACC equivalent compensation.
- There are limitations on when compensation is available, for example compensation may be available for more serious, enduring injuries, and not for temporary pain or discomfort or less serious or curable complaints.

- Unlike ACC, the guidelines do not provide compensation on a no-fault basis:
- The Sponsor may not accept the compensation claim if:
  - Your injury was caused by the investigators, or;
  - There was a deviation from the proposed research plan, or;
  - Your injury was caused solely by you.

An initial decision whether to compensate you would be made the by the sponsor and/or its insurers.

If they decide not to compensate you, you may be able to take action through the Courts for compensation, but it could be expensive and lengthy, and you might require legal representation. You would need to be able to show that your injury was caused by participation in the trial.

You are strongly advised to read the Industry Guidelines and ask questions if you are unsure about what they mean for you.

If you have private health or life insurance, you may wish to check with your insurer that taking part in this study won't affect your cover.

5 WHAT WILL HAPPEN TO MY TEST SAMPLES?

Blood, urine and stool (MAD cohorts only) samples will be collected throughout the study. These samples will be used for various tests. Some of the samples will be used for regular routine blood counts and blood chemistry, and to monitor your general health.

All these routine samples will be sent to LabPlus for testing and destroyed after 3 months by internationally accepted means.

All other study samples (PK, PD and biomarkers) will be sent to central laboratories in Colorado Springs, Colorado, USA (Pyxant Labs), Singapore (LabCorp), Melbourne, Australia (360biolabs), and Adelaide, Australia (GNOMIX) for testing and destroyed after 3 years by internationally accepted means

The maximum amount of blood collected from each participant during the study will be up to 210 mL (SAD cohorts) and up to 520mL (MAD cohorts). For comparison, a standard blood donation at a blood collection centre, is about 470 mL. Samples collected by NZCR will be identified by your study number, year of birth, initials, and sex, to allow study doctors to quickly respond to any abnormal results. Before the results of these tests are sent to the Sponsor, your identifiable information will be removed and replaced with a code.

The proposed blood tests include a screening test for HIV and for viral Hepatitis B and C, as well as a COVID-19 test. Signing the consent form means that you agree to have this testing performed. It is important to understand that a positive screening test does not necessarily mean you have the disease. Should you receive a positive screening result for HIV or either Hepatitis B or C, then the study doctors will provide initial counselling and medical advice and will assist in arranging any follow up tests that you require. HIV, Hepatitis B and C, and COVID-19 are all 'notifiable diseases', which means that it is required by law to notify government health authorities of any new cases.

5.1 Are There Any Cultural Considerations?

You may hold beliefs about sacred and shared values about your tissue samples and/or data originating from this tissue. The cultural issues associated with sending your tissue samples and data overseas and/or storing your tissue and data should be discussed with your family/whanau as appropriate. If you need cultural support this can be provided. Please let us know and we will arrange this for you, or you can ring the Māori cultural support number at the bottom of this Participant Information Sheet and Consent Form. Cultural support is different to knowing more about the study. In these cases, we can arrange for an Investigator to come and

talk to you and your whānau. Due to your samples being sent to countries outside of New Zealand, a karakia will not be able to be performed at the time of your sample disposal.

Personal and health information is a tāonga and will be treated accordingly. The following data sovereignty principles are in place to ensure that the data generated from this research is protected and may benefit Māori now and into the future. Clinical trials are often driven by Sponsor companies overseas and involve very few Māori as participants. However, NZCR will consider the principles of whakapapa, whanaungatanga, rangatiratanga, kotahitanga, manaakitanga and kaitiakitanga throughout the study.

6 WHAT ARE THE RIGHTS OF PARTICIPANTS IN THE STUDY?

6.1 Participation is Voluntary

Participation in any research study is voluntary. If you do not wish to take part, you do not have to. If you decide to take part and later change your mind, you are free to withdraw from the study at any stage. This will not affect your routine treatment/medical care which you may otherwise receive, or with NZCR.

If you do decide to take part, you will be given this Participant Information and Consent Form to sign and you will be given a copy to keep.

6.2 New Information

Sometimes during the course of a research study, new information becomes available about the treatment that is being studied. If this happens, your study doctor will tell you about it and discuss with you whether you want to continue in the research study. If you decide to withdraw, your study doctor will make arrangements for your regular health care to continue. If you decide to continue in the research study you may be asked to sign an updated consent form.

Also, on receiving new information, your study doctor might consider it to be in your best interests to withdraw you from the research study. If this happens, he/ she will explain the reasons and arrange for your regular health care to continue.

6.3 Privacy and Confidentiality and Right to Access Information Collected During the Study

What will Happen to my Information?

During this study, the study doctors, researchers, nurses and other NZCR staff will record information about you and your study participation. This includes the results of any study assessments. Your name, address, and phone number will be on the demographics page for the study so we can identify you correctly at visits and contact you. This information may be used to obtain health records (detailed below) but is not supplied to anyone overseas. If needed, information from your hospital records and your usual doctor (GP) may also be collected, and your GP may be notified about your participation in the study. You cannot take part in this study if you do not consent to the collection of this information.

Identifiable Information
Identifiable information is any data that could identify you (e.g., your name, date of birth, or address). The following groups may have access to your identifiable information:

- NZCR staff (to complete study assessments).
- Local laboratory staff, to process and report your screening and safety tests.
- Study monitors authorised by the Sponsor, to make sure the study is being run properly and that the data collected is accurate.

- Sponsor and its authorised representatives if you make a compensation claim as a result of study-related injury. Identifiable information is required to assess your claim.
- Sponsor, its authorised representatives, ethics committees, or government agencies from New Zealand or overseas, if the study and/or NZCR is audited. Audits are done to make sure that participants are protected, the study is run properly, and the data collected is correct.
- Your GP, if a study test gives an unexpected result that could be important in terms of your health. This allows appropriate follow-up to be arranged.
- The Medical Officer of Health, if you return a positive test for COVID-19, HIV, HBV, or HCV.

Rarely, it may be necessary for the study doctor to share your information with other people – for example, if there is a serious threat to public health or safety, or to the life or health of you or another person OR if the information is required in certain legal situations

De-identified (Coded) Information
To make sure your personal information is kept confidential, information that identifies you will not be included in any study information sent to, or generated by, the Sponsor. Instead, you will be identified by a code. NZCR will keep a list linking your code with your name, so that you can be identified by your coded data if needed.

The following groups may have access to your coded information, which will be sent and stored overseas:

- The Sponsor, for the purposes of this study.
- People and companies working with or for the Sponsor, for the purposes of this study.
- Regulatory or other governmental agencies worldwide.

Anonymised Information
The Sponsor may remove the code from your de-identified information – this is called 'anonymisation'. This makes it very difficult (but not impossible) to identify the information that belongs to you. The Sponsor may use this information for future research (see below).

Future Research Using Your Information
Your coded information may be used for future research related to the development of TLC-2716 and/or relating to severe dyslipidaemias, NASH, or similar diseases.

This future research may be conducted overseas. You will not be told when future research is undertaken using your information. Your information may be shared widely with other researchers or companies. Your information may also be added to information from other studies, to form much larger sets of data.

You will not get reports or other information about any / some research that is done using your information.

Your information may be used indefinitely for future research unless you withdraw your consent. However, it may be extremely difficult or impossible to access your information or withdraw consent for its use once your information has been shared for future research.

Security and Storage of Your Information
During the study, your information will be stored on paper forms at NZCR and electronically on secure servers. When the study has finished, paper forms will be transferred to a secure site and stored for at least 15 years, then destroyed. De-identified information in electronic form will remain on a secure platform and will be retained indefinitely. Storage will comply with local and/or international data security guidelines.

Risks
Although efforts will be made to protect your privacy, absolute confidentiality of your information cannot be guaranteed. Even with coded and anonymised information, there is no guarantee that you cannot be

identified. While the risk is currently very small, the chance that someone might access and misuse your information (for example, by making it harder for you to get or keep a job or health insurance) might increase in the future as people find new ways of tracing information.

Your coded information is being sent overseas. Other countries may have lower levels of data protection than New Zealand. There may be no New Zealand representation on overseas organisations which make decisions about the use of your information. There is a risk that overseas researchers may work with information in a way that is not culturally appropriate for New Zealanders.

7 WHAT WILL HAPPEN AFTER THE STUDY ENDS, OR IF I WANT TO PULL OUT?

7.1 If You Decide to Withdraw

You may withdraw your consent for the collection and use of your information at any time, by informing your study doctor. Please notify a member of the research team before you withdraw. This notice will allow that person or the research supervisor to discuss any health risks or special requirements linked to withdrawing, such as follow up visits.

If you withdraw your consent, your study participation will end, and the study team will stop collecting information from you. Information collected up until your withdrawal from the study will continue to be used and included in the study. This is to protect the quality of the study.

7.2 Why the Study Might be Unexpectedly Stopped

This research study may be stopped unexpectedly for a variety of reasons. These may include a decision by the study sponsor or unacceptable side effects observed in the study cohorts prior to or during your participation.

7.3 Results

When the study ends the data must be analysed, so the results of the study may not be available until about a year after the research finishes. The study doctors and/or Sponsor may decide to discuss or publish the results of the study. This may include publication in journals, presentation at conferences or other professional forums. In any publication, information will be provided in such a way that you cannot be identified. Results of the study will be provided to you if you wish. If you do not wish to receive a summary of the study results when they become available, then please inform the NZCR staff.

Appendix 1. Single Ascending Dosing (SAD) Schedule of Assessments

**Period**	**Screening**	**Treatment/In-patient stay**					**Follow-Up**
**Study Day**	**-28 to -2**	**-1**	**1**	**2**	**3**	**4**	**EOS 15**
Admission to the unit		X					
Discharge from the unit						X	
Physical Exam	X	X	As required (symptom-directed)			X	X
Vital Signs	X	X	X	X	X	X	X
ECG	X	X	X	X		X	X
BMI (Height & Weight)*[1]	X	X				X	X
Drug and Alcohol Testing	X	X					
COVID-19 Testing	X	X	As necessary				
Pregnancy Blood/Urine[2] Test (for women who are able to have children)	X	X				X	X
FSH Test (if applicable for post-menopausal females)	X						
Hepatitis/HIV Testing	X						
Dose Administration			X				
Routine Blood & Urine Tests	X	X	X	X	X	X	X
Biomarkers/PD Blood Tests		-	X				
PK Blood & Urine Tests			X	X	X	X	

BMI = body mass index; ECG = electrocardiogram; EOS = end of study; FSH = follicle stimulating hormone; ; PD = pharmacodynamics; PK = pharmacokinetics

[1] Height is only taken at screening
[2] Urine pregnancy test is done at admission, pregnancy blood test is done at all other time points.

Appendix 1. Multiple Ascending Dosing (MAD) Schedule of Assessments

Period	Screening	Treatment Period/Inpatient stay															Follow Up
**Study Day(s)**	**-28 to -1**	**-2**	**-1**	**1**	**2**	**3**	**4**	**5**	**6**	**7**	**8-9**	**10**	**11-13**	**14**	**15-16**	**17**	**28 EOS**
Admission to the unit		X															
Discharge from the unit																X	
Physical Exam	X	X	As required (symptom-directed)													X	X
Vital Signs	X	X	X	X	X	X		X		X		X		X		X	X
ECG	X	X		X		X				X				X	X	X	
BMI (Height & Weight)[3]	X	X								X						X	X
Drug and Alcohol Testing	X		X														
COVID-19 Testing	X	X															
Hepatitis/HIV Testing	X																
Dose Administration			X[4]	X	X	X	X	X	X	X	X	X	X	X			
Routine Blood & Urine Tests	X		X	X	X	X		X		X		X		X		X	X
PK Blood Tests				X		X				X				X	X	X	
Pregnancy Blood or Urine[5] Test	X	X														X	X
FSH test (post-menopausal women)	X																
PD & Biomarker Blood Tests			X	X						X				X			
Stool Sample			X											X			

BMI = body mass index; ECG = electrocardiogram; EOS = end of study; FSH = follicle stimulating hormone; PD = pharmacodynamics; PK = pharmacokinetics

[3] Height only taken at screening
[4] On day -1, all participants will receive a placebo dosing. Dosing with study drug/placebo where you are randomised and blinded to which arm you are in.
[5] Urine pregnancy test is done at admission, pregnancy blood tests are done at all other time points. These tests will only be done on women who are able to have children.

8 WHO DO I CONTACT FOR MORE INFORMATION OR IF I HAVE CONCERNS?

If you have any questions, concerns, or complaints about the study at any stage, you can contact:

Prof Ed Gane, Principal Investigator
Phone: (09) 373 3474 or 0800STUDIES (08007883437)
Email: waterfall@nzcr.co.nz

If you want to talk to someone who isn't involved with the study, you can contact an independent health and disability advocate on:

Phone:	0800 555 050
Fax:	0800 2 SUPPORT (0800 2787 7678)
Email:	advocacy@advocacy.org.nz
Website:	https://www.advocacy.org.nz/

Māori cultural support is available through:

The Office of the Chief Advisor Tikanga, He Kamaka Waiora, Waitematā and Auckland District Health Board:

Mobile:	021 0203 1167
Phone:	09 486 8320 ext 43204
Email:	hkwresearch@waitematadhb.govt.nz

You can also contact the health and disability ethics committee (HDEC) that approved this study on:

Phone:	0800 4 ETHIC (438 442)
Email:	hdecs@health.govt.nz

9 WHAT ABOUT ANY OTHER QUESTIONS I MAY HAVE?

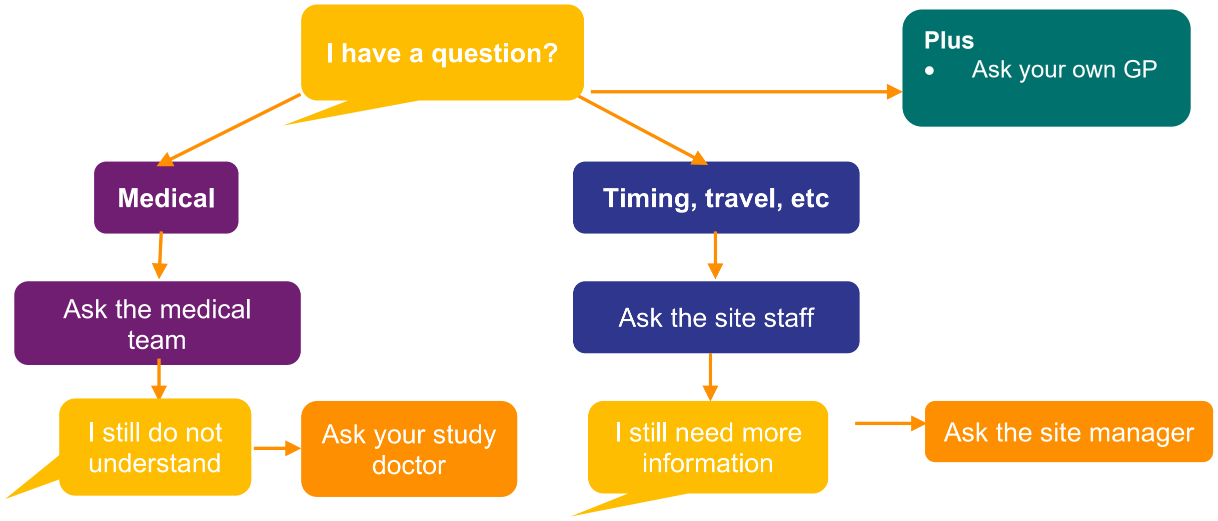

10 DO I HAVE TO DECIDE STRAIGHT AWAY?

No, you do not have to decide straight away. You should take some time to consider whether or not to participate, we will be in touch in a week or so to discuss your decision**.** The following steps are useful in helping you reach a decision.

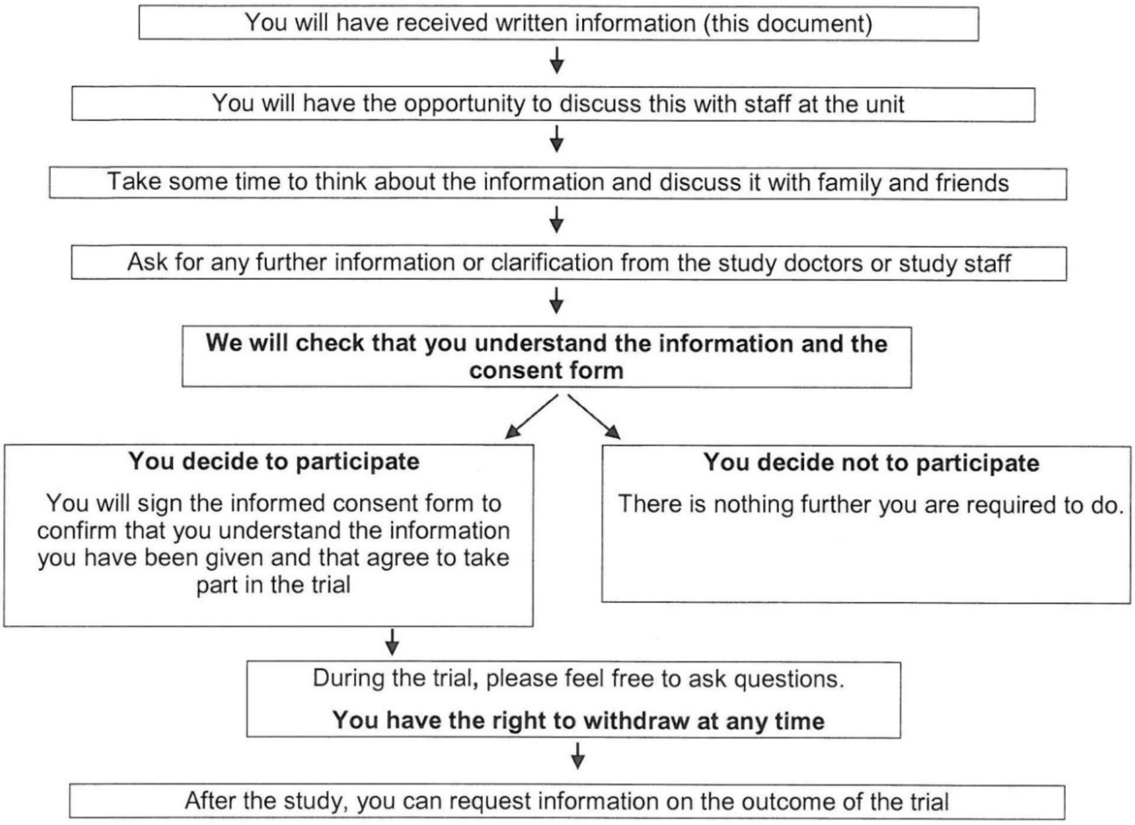

CONSENT FORM
(PART C)

**Short Title:**	A Study to Evaluate Single and Multiple Doses of TLC-2716 in Healthy Participants
**Protocol Number:**	2716-CL-101
**Principal Investigator:**	Prof Ed Gane

***Please let study staff know if you require an interpreter*.**

Declaration by participant:

- I have read or have had read to me in my first language, and I understand the Participant Information Sheet.
- I have been given sufficient time to consider whether or not to participate in this study.
- I have had the opportunity to use a legal representative, whanau/ family support or a friend to help me ask questions and understand the study.
- I am satisfied with the answers I have been given regarding the study and I have a copy of this consent form and information sheet.
- I understand that taking part in this study is voluntary (my choice) and that I may withdraw from the study at any time without this affecting my medical care.
- I consent to the research staff collecting and processing my information, including information about my health.
- If I decide to withdraw from the study, I agree that the information collected about me up to the point when I withdraw may continue to be processed.
- I understand that there may be risks associated with the treatment in the event of myself or my partner becoming pregnant. I undertake to inform my partner of the risks and to take responsibility for the prevention of pregnancy.
- I agree to my tissue samples being sent overseas, and I am aware that these samples will be disposed of using established guidelines for discarding biohazard waste.
- I agree to an approved auditor appointed by the New Zealand Health and Disability Ethic Committees, or any relevant regulatory authority or their approved representative reviewing my relevant medical records for the sole purpose of checking the accuracy of the information recorded for the study.
- I understand that my participation in this study is confidential and that no material, which could identify me personally, will be used in any reports on this study.
- I understand the compensation provisions in case of injury during the study.
- I know who to contact if I have any questions about the study in general.
- I understand my responsibilities as a study participant.
- I understand that I will receive a summary of the study results and if I do not want to receive this, I will inform site staff.
- I consent to my GP or current provider being informed about my participation in the study and of any significant abnormal results obtained during the study.

**Statement by Participant**	I hereby consent to take part in this study. I understand that I will receive a signed copy of this consent form for my records.<br><br>___________________________________ (full name)<br><br>___________________________________ (signature)<br><br>__ __ / __ __ __ / __ __ __ __ (Date DD/MMM/YYYY)

Statement by Consenter (Investigator/designee) I have discussed this study with the above-named participant. The participant appeared to fully understand the information provided about the study.

_________________________________ (full name)

_________________________________ (signature)

_________________________________ (project role)

__ __ / __ __ __ / __ __ __ __ (Date DD/MMM/YYYY)

CLINICAL STUDY PROTOCOL

**Study Title:**	A Phase 1 Study to Evaluate the Safety, Tolerability, Pharmacokinetics, and Pharmacodynamics of Single and Multiple Ascending Doses of TLC-2716 in Healthy Subjects
**Sponsor:**	The Liver Company, Inc.<br>2671 Marshall Drive<br>Palo Alto, CA, 94303, USA
**IND Number:**	This is a non-IND study
**EudraCT Number:**	Not Applicable
**Clinical Trials.gov Identifier:**	NCT05483998
**Indication:**	Severe dyslipidemia and NASH
**Protocol ID:**	2716-CL-101
**TLC Medical Monitor**	Name: Ryan Huss, MD<br>Telephone: +1 (850) 532-9083<br>Email: ryan@tlc-tx.com
**Protocol Version/Date:**	Original: 17 June 2022<br>Amendment 1: 04 August 2022

CONFIDENTIALITY STATEMENT

Information contained in this document, including unpublished data, is the property or under control of The Liver Company, Inc., and is provided to you in confidence as an investigator, potential investigator, or consultant, for review by you, your staff, and an applicable institutional review board or independent ethics committee. The information is only to be used by you in connection with authorized clinical trials of the investigational drug described in the protocol. You will not disclose any of the information included in this document without written authorization from The Liver Company, Inc., except to the extent necessary to obtain informed consent from those persons to whom the investigational drug may be administered.

TABLE OF CONTENTS

TABLE OF CONTENTS 2
LIST OF APPENDICES 6
LIST OF IN-TEXT TABLES 6
LIST OF IN-TEXT FIGURES 6
PROTOCOL SYNOPSIS 7
GLOSSARY OF ABBREVIATIONS AND DEFINITION OF TERMS 17
1. INTRODUCTION 20
1.1. Background 20
1.1.1. Severe Dyslipidemias 20
1.1.1.1. Severe Hypertriglyceridemia (SHTG) 20
1.1.1.2. Familial Hypercholesterolemia (FH) 20
1.1.2. Nonalcoholic Steatohepatitis (NASH) 21
1.2. TLC-2716 22
1.2.1. General Information 22
1.2.2. Nonclinical Pharmacology 23
1.2.3. Nonclinical Pharmacokinetics (PK) 23
1.2.4. Nonclinical Toxicology 24
1.2.5. Clinical Trials of TLC-2716 25
1.3. Rationale for the Current Study 25
1.3.1. Rationale for the Study Design 25
1.3.2. Rationale for Dose Selection 26
1.4. Risk/Benefit Assessment for the Study 27
1.5. Compliance 27
2. OBJECTIVES 28
3. STUDY DESIGN 29
3.1. Study Design Description 29
3.1.1. Part A: Single-Ascending Dose [SAD] (Cohorts 1-5) 29
3.1.2. Part B: Multiple-Ascending Dose [MAD] (Cohorts 6-10) 31
3.1.3. Part C: Adaptive SAD and/or MAD (Cohorts 11-15) 32
3.2. Dose Escalation Criteria 34
3.3. Protocol-Specific Stopping Criteria 35
3.3.1. Subject Study Drug Discontinuation Criteria 35
3.3.2. Study-Specific Stopping Criteria 36
3.4. Duration of Dosing 36
3.5. Clinic Confinement 37
3.6. Pharmacokinetic Assessments 37
3.7. Pharmacodynamic Assessments 37
3.8. Biomarker Sample for Optional Genomic Research 37
3.9. Safety Assessments 37
3.10. End of Study 38
4. SUBJECT POPULATION 39
4.1. Number of Subjects and Subject Selection 39
4.1.1. Subject Replacement 39
4.2. Inclusion Criteria 39
4.3. Exclusion Criteria 40

5. STUDY DRUGS 43
5.1. Randomization, Blinding, and Treatment Codes Access 43
5.1.1. Randomization 43
5.1.2. Blinding 43
5.1.3. Procedures for Breaking Treatment Codes 43
5.2. Description and Handling of Study Drugs 44
5.2.1. Formulation 44
5.2.2. Packaging and Labeling 44
5.2.3. Storage and Handling 44
5.3. Administration of Study Drug 44
5.3.1. Fasting and Meals 45
5.4. Dispensing, Accountability, and Disposal or Return of Study Drug 45
5.5. Concomitant Medications and Other Protocol Restrictions 46
5.5.1. Concomitant Medications 46
5.5.2. Other Protocol Restrictions 46
6. STUDY ASSESSMENTS 48
6.1. Subject Enrollment and Treatment Assignment 48
6.2. Pretreatment Assessments 54
6.2.1. Screening Visit 54
6.2.2. Admission Assessments 54
6.2.2.1. Admission 54
6.2.2.2. Clinic Confinement 55
6.3. Check-In Assessments 55
6.4. Treatment Assessments 55
6.5. Pharmacokinetic Assessments 55
6.5.1. Plasma PK Collection 55
6.6. Pharmacodynamic Assessments 56
6.6.1. Plasma and/or Serum Pharmacodynamic Assessments 56
6.6.2. Stool Pharmacodynamic Assessments 57
6.6.3. Biomarker Assessments 57
6.7. Safety Assessments 58
6.7.1. Body Mass Index 59
6.7.2. Physical Examination 59
6.7.3. Vital Signs 59
6.7.4. Electrocardiogram Assessment 59
6.7.5. Clinical Laboratory Tests/Assessments 60
6.7.5.1. Blood Sampling 60
6.7.5.2. Urine Samples 61
6.7.6. Estimated Glomerular Filtration Rate Using MDRD 61
6.7.7. Adverse Events/Concomitant Medications/Protocol Restrictions 61
6.8. Posttreatment Assessments – Follow-up Visit 61
6.9. Management of Subjects Following Premature Discontinuation of Study Drug or Study 61
6.10. Optional Future Research 62
7. ADVERSE EVENTS AND TOXICITY MANAGEMENT 63
7.1. Definitions of Adverse Events, Adverse Reactions, and Serious Adverse Events 63
7.1.1. Adverse Events 63
7.1.2. Clinical Laboratory Abnormalities and Other Abnormal Assessments as Adverse Events or Serious Adverse Events 63
7.1.3. Serious Adverse Events 64
7.2. Assessment of Adverse Events and Serious Adverse Events 64
7.2.1. Assessment of Causality for Study Drugs and Procedures 64

7.2.2. Assessment of Severity ... 65
7.3. Investigator Requirements and Instructions for Reporting Adverse Events and Serious Adverse Events ... 65
7.3.1. Requirements for Collection Prior to Study Drug Initiation ... 65
7.3.1.1. Adverse Events ... 65
7.3.1.2. Serious Adverse Events ... 65
7.3.1.3. Serious Adverse Event Paper Reporting Process ... 66
7.4. TLC Reporting Requirements ... 66
7.5. Toxicity Management ... 67
7.6. Special Situations Reports ... 67
7.6.1. Definitions of Special Situations ... 67
7.6.2. Instructions for Reporting Special Situations ... 68
7.6.2.1. Instructions for Reporting Pregnancies ... 68
7.6.2.2. Reporting Other Special Situations ... 68
8. STATISTICAL CONSIDERATIONS ... 70
8.1. Analysis Objectives and Endpoints ... 70
8.1.1. Analysis Objectives ... 70
8.1.2. Primary Endpoint(s) ... 70
8.1.3. Exploratory Endpoint(s) ... 71
8.2. Planned Analyses ... 71
8.2.1. Dose Escalation Analysis ... 71
8.2.2. Final Analysis ... 71
8.3. Analysis Conventions ... 71
8.3.1. Analysis Sets ... 71
8.3.1.1. Safety ... 71
8.3.1.2. Pharmacokinetics ... 71
8.3.1.3. Pharmacodynamics ... 72
8.4. Data Handling Conventions ... 72
8.5. Demographic Data and Baseline Characteristics ... 72
8.6. Safety Analysis ... 72
8.6.1. Extent of Exposure ... 72
8.6.2. Adverse Events ... 72
8.6.3. Laboratory Evaluations ... 73
8.6.4. Other Safety Evaluations ... 73
8.7. Pharmacokinetic Analysis ... 73
8.8. Pharmacodynamics Analysis ... 73
8.9. Pharmacokinetics and Pharmacodynamics Analysis ... 74
8.10. Sample Size ... 74
9. RESPONSIBILITIES ... 75
9.1. Investigator Responsibilities ... 75
9.1.1. Good Clinical Practice ... 75
9.1.2. Financial Disclosure ... 75
9.1.3. Institutional Review Board/Independent Ethics Committee Review and Approval ... 75
9.1.4. Informed Consent ... 75
9.1.5. Confidentiality ... 76
9.1.6. Study Files and Retention of Records ... 76
9.1.7. Case Report Forms ... 77
9.1.8. Study Drug Accountability and Return ... 78
9.1.9. Inspections ... 78
9.1.10. Protocol Compliance ... 78
9.2. Sponsor Responsibilities ... 79

9.2.1. Protocol Modifications ... 79
9.2.2. Study Report ... 79
9.3. Joint Investigator/Sponsor Responsibilities ... 79
9.3.1. Payment Reporting ... 79
9.3.2. Access to Information for Monitoring ... 79
9.3.3. Access to Information for Auditing or Inspections ... 79
9.3.4. Study Discontinuation ... 80
10. REFERENCES ... 81
11. APPENDICES ... 84

LIST OF APPENDICES

Appendix 1. Investigator Signature Page 85
Appendix 2. Management of Clinical and Laboratory Adverse Events 86
Appendix 3. Pregnancy Precautions, Definition for Female of Childbearing Potential, and Contraceptive Requirements 87
Appendix 4. Common Terminology Criteria for Adverse Events (CTCAE) Grading Scale v5.0 90

LIST OF IN-TEXT TABLES

Table 1. Estimated Dose Margins Following Oral Administration of TLC-2716 Based on 4-week, GLP Chronic Toxicology Studies 26
Table 2. Treatments to be Administered in Cohorts 1-5 30
Table 3. Treatments to be Administered in Cohorts 6-10 32
Table 4. Treatments to be Administered in Cohorts 11-15 33
Table 5. Schedule of Assessments: SAD Cohorts in Parts A and C 49
Table 6. Schedule of Assessments: MAD Cohorts in Parts B and C 51

LIST OF IN-TEXT FIGURES

Figure 1. Study Schema – Part A, SAD Cohorts 1-5 31
Figure 2. Study Schema – Part B, MAD Cohorts 6-10 34

PROTOCOL SYNOPSIS
The Liver Company, Inc.

Study Title: A Phase 1 Study to Evaluate the Safety, Tolerability, Pharmacokinetics, and Pharmacodynamics of Single and Multiple Ascending Doses of TLC-2716 in Healthy Subjects

IND Number: This is a non-IND study

EudraCT Number: Not Applicable

ClinicalTrials.gov Identifier: NCT05483998

Study Centers Planned: Single Phase 1 center in New Zealand

Objectives:

Primary Objectives:

- To evaluate the safety and tolerability of escalating single and multiple doses of TLC-2716
- To characterize the single- and multiple-dose pharmacokinetics (PK) of TLC-2716 and its metabolite(s)

Exploratory Objectives:

- To evaluate the pharmacodynamics (PD) of liver X receptor (LXR) inverse agonism by TLC-2716 as measured by metabolic parameters including fasting lipids and serum biomarkers of *de novo* lipogenesis (DNL)
- To characterize TLC-2716 dose and/or exposure-response relationships for PD markers
- To evaluate exploratory biomarkers

Study Design:

- **Part A; Single-Ascending Dose [SAD] (Cohorts 1-5):** Randomized, sponsor-unblinded, placebo-controlled, single-ascending doses of TLC-2716
- **Part B; Multiple-Ascending Dose [MAD] (Cohorts 6-10):** Randomized, sponsor-unblinded, placebo-controlled, multiple-ascending doses of TLC-2716
- **Part C; Adaptive SAD and/or MAD (Cohorts 11-15):** Randomized, sponsor-unblinded, placebo-controlled, single- and/or multiple-ascending doses with adaptive dose selection of TLC-2716

Number of Subjects Planned: Up to 150 subjects total (8 active, 2 placebo-to-match [PTM] per cohort):

- Part A (SAD); Cohorts 1-5: 50 subjects (10 per cohort)
- Part B (MAD); Cohorts 6-10: 50 subjects (10 per cohort)
- Part C (Adaptive SAD and/or MAD); Cohorts 11-15: 50 subjects (10 per cohort)

Target Population: Healthy male and non-pregnant, non-lactating female subjects, 18-55 years of age, inclusive

Duration of Dosing:

- Part A (SAD): 1 day
- Part B (MAD): 15 days (including PTM dosing for all subjects on Day -1)
- Part C (Adaptive SAD and/or MAD): 1 or 15 days (including PTM dosing for all multiple-dose subjects on Day -1)

Study Duration:

- Screening window: 28 days
- Part A (SAD): Up to 18 days (not including the Screening window)
- Part B (MAD): Up to 32 days (not including the Screening window)
- Part C (Adaptive SAD and/or MAD): Up to 32 days (not including the Screening window)

Main Eligibility Criteria:

Eligible subjects will be an approximately even distribution of healthy male and non-pregnant, non-lactating female subjects, age 18 to 55 years inclusive, with a body mass index (BMI) of $19 \leq \text{BMI} \leq 35\ \text{kg/m}^2$, normal 12-lead electrocardiogram (ECG), or one with abnormalities that are considered clinically insignificant by the investigator, normal renal function (estimated glomerular filtration rate calculated using the Cockcroft-Gault equation ≥ 80 mL/min), liver biochemistry tests within the normal range (total bilirubin > 1.0 to ≤ 1.5x ULN permitted in subjects with a medical history of Gilbert's syndrome). In any MAD cohorts, attempts will be made to enroll at least 3 subjects in each cohort with Screening triglycerides (TG) ≥ 150 mg/dL and/or low-density lipoprotein cholesterol (LDL-C) ≥ 130 mg/dL. Otherwise, eligible subjects must have no significant medical history, and be in good general health, in the opinion of the investigator, as per the Screening evaluations performed within 28 days prior to the scheduled first dose in any cohort.

Study Procedures/Frequency:

Following the completion of Screening and admission assessments, eligible subjects will be enrolled on Day -1.

This study will proceed in 3 parts governed within and between parts by reviews of safety and tolerability data and application of stopping criteria as detailed in Section 3.3.

Based on safety and available PK and/or PD data, and at the discretion of the sponsor, the adaptive cohorts may not be initiated or may be held until additional data are available.

Part A (SAD), Pre-specified Cohorts 1-5

Part A will evaluate the safety, tolerability, PK, and PD of single-ascending doses of oral TLC-2716 or PTM under fed conditions. Part A will proceed in up to 5 dose-escalation cohorts and will be governed by a review of safety and tolerability, any relevant and available PK and/or PD data, and study-specific stopping criteria.

Within each cohort, 10 unique subjects will be randomized 4:1 to receive either blinded TLC-2716 (N = 8) or PTM (N = 2). All study drugs in Part A will be administered in the morning following a standard meal.

At the initiation of each cohort in Part A, prior to randomization of the entire cohort, two sentinel subjects will be randomized, one to TLC-2716 at the dose to be evaluated in the planned cohort, and one to corresponding PTM. Enrollment and randomization of the remaining eight subjects in each

cohort will be determined upon evaluation of all safety and tolerability data through Day 2 for these two sentinel subjects.

For Cohorts 2-5, initiation of single-dose administration will be permitted after evaluation of cumulative blinded safety data, and any relevant and available PK and/or PD data up to and including Day 4, following single-dose administration of the previous cohort.

The cohorts and study treatments for Part A are as follows:

Cohort	Day 1
**1**	0.5 mg TLC-2716 (1 x 0.5 mg) or PTM capsule SD, fed
**2**	2 mg TLC-2716[a] (1 x 2 mg) or PTM capsule SD, fed
**3**	6 mg TLC-2716[a] (3 x 2 mg) or PTM capsules SD, fed
**4**	12 mg TLC-2716[a] (2 x 5 mg; 1 x 2 mg) or PTM capsules SD, fed
**5**	20 mg TLC-2716[a] (4 x 5 mg) or PTM capsules SD, fed

PTM = placebo-to-match; SD = single dose
a The planned doses may be modified based on the observed safety, tolerability, and any relevant and available PK and/or PD data. Any modified dose will be within 3-fold of a dose previously tested.

Part B (MAD), Pre-specified Cohorts 6-10

Part B will proceed in up to 5 dose-escalation cohorts and will be governed by reviews of safety and tolerability, and any relevant and available PK and/or PD data, and study-specific stopping criteria. Within each cohort, 10 unique subjects will be randomized 4:1 to receive either blinded TLC-2716 (N = 8) or PTM (N = 2) QD for 14 days.

All study drugs in Part B will be administered in the morning following a standard meal.

Part B (Cohort 6) may be initiated after the evaluation of cumulative safety, tolerability, and any relevant and available PK and/or PD data from all subjects enrolled in Cohort 3 in Part A (up to and including Day 4).

For Cohorts 7-10, dosing may be initiated after evaluation of cumulative safety, tolerability, and any relevant and available PK and/or PD data from all subjects enrolled in the previous cohort in Part B through Day 14.

In both Parts A and B, escalation to a dose higher than previously studied may occur only in the absence of dose-limiting toxicity and/or not meeting any prespecified stopping criteria.

The cohorts and study treatments for Part B are as follows:

Cohort	Day -1	Days 1-14
**6**	PTM capsule SD	0.5 mg TLC-2716 (1 x 0.5 mg) or PTM capsule, fed
**7**	PTM capsule SD	2 mg TLC-2716[a] (1 x 2 mg) or PTM capsule, fed
**8**	PTM capsules SD	6 mg TLC-2716[a] (3 x 2 mg) or PTM capsules, fed
**9**	PTM capsules SD	12 mg TLC-2716[a] (2 x 5 mg; 1 x 2 mg) or PTM capsules, fed
**10**	PTM capsules SD	20 mg TLC-2716[a] (4 x 5 mg) or PTM capsules, fed

PTM = placebo-to-match; SD = single dose
a The planned doses may be modified based on the observed safety, tolerability, and any relevant and available PK and/or PD data. Any modified dose will be within 3-fold of a dose previously tested.

Part C (SAD and/or MAD), Adaptive Cohorts 11-15

Based on available safety, PK, and/or PD data from Part A and Part B (if applicable), doses for Part C (optional, adaptive Cohorts 11-15) will be chosen up to a total daily dose of 50 mg. The frequency of dosing (QD or twice daily [BID]), duration of dosing (single on one day or multiple over 14 days), and fasting versus fed status may also be modified versus Parts A and B. If dosing is administered in the fed state, standard meal conditions may also be modified at sponsor discretion. QD doses will be administered in the morning following a standard meal, and BID doses will be administered after a standard morning meal and evening snack, with the second dose administered 12 hours (± 10 minutes) after the morning dose. Once determined, dose level, duration of dosing, frequency of dosing, and meal conditions will remain consistent within a cohort. For any multiple-dose cohort in Part C with BID dosing, both morning and evening doses must be administered on Day 14.

Within each cohort, 10 unique subjects will be randomized 4:1 to receive up to a total daily dose of 50 mg TLC-2716 (N = 8) or PTM (N = 2) for either 1 day (as in Part A) or 14 days (as in Part B). Adaptive, multiple-dose cohorts in Part C may be initiated in parallel with previous cohorts if the total daily dose under evaluation is at or below a dose already evaluated in Part B. If doses chosen in 2 or more adaptive cohorts exceed the dose evaluated in a previous cohort in Part B, these cohorts will be conducted in a staggered manner (lowest dose first), with the same stopping criteria as detailed in Section 3.3. Additionally, if the total daily dose in any cohort in Part C is greater than has been evaluated in any prior cohort, then sentinel dosing including one subject randomized to placebo and one subject to TLC-2716 will be included. Randomization of the remaining eight subjects in the cohort will be based on evaluation of all safety and tolerability data through Day 2 for these two sentinel subjects.

The sponsor, in consultation with the investigator, may choose not to initiate any or all adaptive cohorts if deemed unnecessary.

The cohorts and study treatments for Part C are as follows:

Cohort	Day -1[a]	Single Dose (Day 1) or Multiple Doses (Days 1-14)
**11-15**	PTM capsule(s)<br>QD or BID	≤ 50 mg total daily dose TLC-2716 or PTM,<br>administered QD or BID, fasting or fed

BID = twice daily; PTM = placebo-to-match; QD = once daily
a Dosing of PTM on Day -1 will only be performed in multiple-dose adaptive cohorts (as in Part B).

Study Visits and Confinement

Following Screening and admission procedures, eligible subjects will be confined to the study center beginning at admission (Day -1 for SAD cohorts, Day -2 for MAD cohorts) until the completion of assessments on Day 4 (SAD cohorts) or Day 17 (MAD cohorts). Subjects will return 14 ± 2 days after the last dose for an in-clinic follow-up visit (i.e., Day 15 [± 2 days] for SAD cohorts; Day 28 [± 2 days] for MAD cohorts).

Pharmacokinetic Assessments

Plasma PK:

SAD Cohorts:

PK sampling will occur relative to the morning dosing of TLC-2716 or PTM at the following time points for each cohort:

Day 1: Pre-dose (≤ 10 minutes prior to dosing), 0.25, 0.5, 1, 1.5, 2, 2.5, 3, 3.5, 4, 6, 12, 24, 48, and 72 hours post-dose

MAD Cohorts:

Day 1: Pre-dose (≤ 10 minutes prior to dosing), 0.25, 0.5, 1, 1.5, 2, 2.5, 3, 3.5, 4, 6, and 12 hours post-dose

Days 3, and 7: Pre-dose (≤ 10 minutes prior to dosing), 2 and 4 hours post-dose

Day 14: Pre-dose (≤ 10 minutes prior to dosing), 0.25, 0.5, 1, 1.5, 2, 2.5, 3, 3.5, 4, 6, 12, 24, 48, and 72 hours post-dose

If in any MAD cohort in Part C, BID administration is evaluated, PK assessments should be performed at the following time points:

Day 1: Pre-dose morning (≤ 10 minutes prior to dosing), 0.25, 0.5, 1, 1.5, 2, 2.5, 3, 3.5, 4, 6, and 12 hours post morning dose (12-hour time point collected prior to evening dose)

Days 3 and 7: Pre-dose morning (≤ 10 minutes prior to dosing), 2, and 4 hours post morning dose

Day 14: Pre-dose morning (≤ 10 minutes prior to dosing), 0.25, 0.5, 1, 1.5, 2, 2.5, 3, 3.5, 4, 6, and 12 hours post morning dose (12-hour time point collected prior to evening dose), 12.5, 13, 14, 16, 18, 24, 48, and 72 hours post-dose

Plasma concentrations of TLC-2716 (and its metabolites, as applicable) will be determined and other PK parameters evaluated, as appropriate. Sampling time points may be modified by the sponsor based upon emerging data and feasibility.

For all cohorts, a single PK sample will be collected at the Early Termination (ET) visit, if applicable.

Pharmacodynamic Assessments

Plasma and/or Serum PD Collection

Blood samples will be collected to measure PD biomarkers for TLC-2716 at the time points described below, where ‘0’ indicates the pre-dose time point in the morning. Blood samples for PD assessments will be collected in the same subject order on each day, including Day -1 (as applicable), in each cohort. For MAD cohorts in Parts B and C, the collection time on Day -1 should match the time of the collection on the day of dosing time points (± 5 minutes).

Relevant PD biomarkers including measurement of DNL, serum angiopoietin-like protein-3 (ANGPTL3), a comprehensive serum lipid profile (e.g., LDL-C, high-density lipoprotein cholesterol [HDL-C], TG, Apolipoprotein B [ApoB]), as well as other exploratory PD markers will be determined and evaluated.

SAD Cohorts:
Day 1: Pre-dose (≤ 10 minutes prior to dosing) and 4 hours post-dose

MAD Cohorts:
Day -1: (all ± 5 minutes of Days 1, 7, and 14 time points as applicable), 0, 2, 4, 6, and 12 hours
Days 1, 7, and 14: Pre-dose (≤ 10 minutes prior to dosing), 2, 4, 6, and 12 hours post-dose

All post-dose timings above are relative to morning dose for QD or BID dosing.

Sampling time points may be modified by the sponsor based on emerging data and assay feasibility.

Additional Biomarker Assessments

To support the evaluation of the effects of TLC-2716 on lipid homeostasis, a separate serum aliquot will be collected for potential exploratory biomarker assessments, including but not limited to inducible degrader of low-density lipoprotein (IDOL), apolipoprotein C and E, and nuclear magnetic resonance (NMR)-based lipid particle profiles.

Whole blood samples will also be collected to measure ABCA1 and ABCG1 mRNA expression, at the time points described below, where '0' corresponds to the immediately pre-dose time point in the morning on Day 1, or later as applicable. Blood samples for biomarkers will be collected in the same subject order on each day, including the pre-dose day (for MAD cohorts), in each cohort. The collection times on Day -1 in MAD cohorts must match the time of the collection on the day of dosing time points (± 5 minutes).

NMR LipoProfile

SAD Cohorts: None

MAD Cohorts:
Days 1 and 14: Pre-dose (≤ 10 minutes prior to dosing), and 2, 4, 6, and 12 hours post-dose

All post-dose timings above are relative to the morning dose for QD dosing or BID dosing.

Exploratory Biomarkers

SAD Cohorts: Day 1: Pre-dose (≤ 10 minutes prior to dosing) and 4 hours post-dose

MAD Cohorts:
Day -1: (all ± 5 minutes of Day 1, 7, and 14 time points as applicable), 0, 2, 4, 6, and 12 hours

Days 1, 7, and 14: Pre-dose (≤ 10 minutes prior to dosing), 2, 4, 6, and 12 hours post-dose

All post-dose timings above are relative to the morning dose for QD dosing or BID dosing.

Whole Blood Collection

SAD Cohorts: None

MAD Cohorts:
Days 1 and 14: Pre-dose (≤ 10 minutes prior to dosing), 4 hours post-dose

Sampling time points may be modified by the sponsor based upon emerging data and assay feasibility.

Stool Collection

All stools will be collected over approximately 24-hour intervals to measure PD and other exploratory biomarkers for TLC-2716 at the time points described below.

SAD Cohorts: None

MAD Cohorts: Single sample collections
Day -1: Day -2 Admission – Day 1 (0, pre-dose)

Day 14: 0 (pre-dose) – 24 hours post-dose

Safety Assessments

SAD Cohorts:

Height: Screening

Weight: Screening, Days -1 (Admission), the day of discharge, and at the in-clinic follow-up visit

BMI: Screening, Days -1 (Admission), the day of discharge, and at the in-clinic follow-up visit

Vital signs (blood pressure, pulse, respiration rate, and body temperature): Screening, Days -1, 1, 2, 3, the day of discharge, and at the in-clinic follow-up visit or at the ET visit (if applicable)

Complete physical exam: Screening, Day -1, the day of discharge, and at the in-clinic follow-up visit or at the ET visit (if applicable)

Symptom-driven physical exam: As needed on days of confinement based on reported signs and symptoms

HIV-1, HBV, HCV serology: Screening

Clinical laboratory tests (hematology, serum chemistry, coagulation, lipids, and urinalysis): Screening, Days -1, 1 (prior to morning meal and 12 hours post-dose), 2, 3, the day of discharge, and at the in-clinic follow-up visit or at the ET visit (if applicable)

Estimated glomerular filtration rate (eGFR): Screening

Serum Pregnancy Test (women of childbearing potential only): Screening, the day of discharge, and at the in-clinic follow-up visit or at the ET visit (if applicable)

Urine Pregnancy Test (women of childbearing potential only): Day -1

Urine Drug and Alcohol Assessments: Screening and Day -1

12-lead ECG: Screening, Days -1, 1 (2 hours post-dose), 2, the day of discharge, and at the in-clinic follow-up visit or at the ET visit (if applicable)

MAD Cohorts:

Height: Screening

Weight: Screening, Days -2, 7, the day of discharge, and at the in-clinic follow-up visit

BMI: Screening, Days -2, 7, the day of discharge, and at the in-clinic follow-up visit

Vital signs: Screening, Days -2, -1, 1, 2, 3, 5, 7, 10, 14, the day of discharge, and at the in-clinic follow-up visit or at the ET visit (if applicable)

Complete physical exam: Screening, Day -2, the day of discharge, and at the in-clinic follow-up visit or at the ET visit (if applicable)

Symptom-driven physical exam: As needed on days of confinement based on reported signs and symptoms

HIV-1, HBV, HCV Serology: Screening

Clinical laboratory tests (hematology, serum chemistry, coagulation, lipids, and urinalysis): Screening, Days -1, 1 (prior to morning meal and 12 hours post-dose), 2, 3, 5, 7, 10, 14, the day of discharge, and at the in-clinic follow-up visit or at the ET visit (if applicable)

Estimated glomerular filtration rate (eGFR): Screening

Serum Pregnancy Test (women of childbearing potential only): Screening, the day of discharge, and at the in-clinic follow-up visit or at the ET visit (if applicable)

Urine Pregnancy Test (women of childbearing potential only): Day -2

Urine Drug and Alcohol Assessments: Screening and Day -2

12-lead ECG: Screening, Days -2, 1 (2 hours post-dose), 3, 7, 14, the day of discharge, and at the in-clinic follow-up visit or at the ET visit (if applicable).

For all subjects, assessment of AEs and concomitant medications will continue throughout the study. Additionally, based on study site protocols and investigator discretion, subjects may be tested for COVID-19 infection at Screening, Admission, and on an as needed basis.

If subjects experience any clinically significant AEs during the in-clinic period, they may remain in-clinic for further observation at the discretion of the investigator. Additional follow-up visits may be undertaken, as needed, for assessment of persistent AEs and laboratory abnormalities.

Optional Genomic Testing

For subjects who provide consent, a separate blood specimen will be collected on Day -1 to isolate DNA for genomic testing. This specimen will be used to study genetic and genomic mechanisms that may contribute to underlying metabolic diseases and outcomes. Stored specimens may also be used to evaluate the association of genetic and genomic markers with study drug response, including metabolism and/or adverse events (AEs), and to determine future treatment predictions for TLC-2716 and/or other approved or exploratory medications. This sample should be collected on Day -1 prior to dosing but may be collected at any time during the study, if necessary.

Protocol-Specific Stopping Criteria

Subject Study Drug Discontinuation Criteria

Study drug will be discontinued for a subject who experiences any of the following:

1. A confirmed, treatment-emergent, treatment-related, serious AE (SAE) or ≥ Grade 3 AE
2. Any ≥ Grade 3 clinically significant laboratory abnormality (confirmed by repeat testing) as defined by the Common Terminology Criteria for Adverse Events (CTCAE) v5.0 for Severity of Adverse Events and Laboratory Abnormalities
3. Symptoms of drug-related hepatotoxicity, and/or ALT or AST > 5 x ULN, or ALT > 3 x ULN and total bilirubin > 2 x ULN or INR > 1.5, confirmed by immediate repeat testing

Study-Specific Stopping Criteria

Dose escalation or study drug dosing of a cohort will be suspended and, based on a full review of the clinical data by The Liver Company (TLC) medical monitor (MM) or designee, may be halted when:

- 1 subject dosed with TLC-2716 experiences a treatment-emergent SAE possibly related to study drug
- 2 subjects dosed with TLC-2716 within 1 cohort experience elevations in liver biochemistry tests of ALT and/or AST > 5 x the ULN, or ALT > 3 x ULN and total bilirubin > 2 x ULN or INR > 1.5, confirmed by immediate repeat testing
- 2 or more subjects dosed with TLC-2716 experience the same ≥ Grade 3 AE possibly related to the study drug

- 2 or more subjects dosed with TLC-2716 experience a clinically significant ≥ Grade 3 laboratory abnormality of similar nature and possibly related to the study drug (confirmed by repeat testing)
- The number and/or severity of AEs justify study discontinuation
- The sponsor requests study discontinuation

Decisions to reinitiate the study will be made in consultation with the sponsor and pending a comprehensive safety review.

Test Product, Dose, and Mode of Administration:

Cohort 1: TLC-2716 0.5 mg (1 x 0.5 mg capsule), administered orally in a single dose in a fed state

Cohort 2: TLC-2716 2 mg (1 x 2 mg capsule), administered orally in a single dose in a fed state

Cohort 3: TLC-2716 6 mg (3 x 2 mg capsules), administered orally in a single dose in a fed state

Cohort 4: TLC-2716 12 mg (2 x 5 mg capsules, 1 x 2 mg capsule), administered orally in a single dose in a fed state

Cohort 5: TLC-2716 20 mg (4 x 5 mg capsules), administered orally in a single dose in a fed state

Cohorts 6-10: TLC-2716 up to 20 mg total daily dose, administered orally in a single dose once daily for 14 days (0.5 mg, 2 mg, and/or 5 mg capsules) in a fed state

Cohorts 11-15: TLC-2716 up to 50 mg total daily dose, administered orally in a single dose or as single or twice daily doses for 14 days (0.5 mg, 2 mg, and/or 5 mg capsules) in a fed or fasted state

Reference Therapy, Dose, and Mode of Administration:

Cohort 1: TLC-2716 0.5 mg PTM (1 x placebo 0.5 mg capsule), administered orally in a single dose in a fed state

Cohort 2: TLC-2716 2 mg PTM (1 x placebo 2 mg capsule), administered orally in a single dose in a fed state

Cohort 3: TLC-2716 6 mg PTM (3 x placebo 2 mg capsules), administered orally in a single dose in a fed state

Cohort 4: TLC-2716 12 mg PTM (2 x placebo 5 mg capsules, 1 x placebo 2 mg capsule), administered orally in a single dose in a fed state

Cohort 5: TLC-2716 20 mg PTM (4 x placebo 5 mg capsules), administered orally in a single dose in a fed state

Cohorts 6-10: TLC-2716 PTM, administered orally in a single dose once daily (0.5 mg, 2 mg, and/or 5 mg placebo capsules) for 14 days in a fed state

Cohorts 11-15: TLC-2716 PTM, administered orally in a single dose or as single or twice daily doses for 14 days (0.5 mg, 2 mg, and/or 5 mg placebo capsules) in a fed or fasted state

All subjects in MAD cohorts in Parts B and C will receive PTM on Day -1, with the number and type of PTM capsule(s) corresponding with the planned dose in each individual cohort.

Criteria for Evaluation:

Safety: Safety will be evaluated by assessment of clinical laboratory tests, ECGs, periodic physical examinations including vital signs at various time points throughout the study, and by the documentation of AEs and concomitant medications.

Efficacy: Not applicable

**Pharmacokinetics:** The following single- or multiple-dose plasma PK parameters of TLC-2716 and its metabolites will be calculated as appropriate: AUC_{last}, AUC_{inf} [single-dose], $\%AUC_{exp}$ [single dose], CL/F [single dose], AUC_{tau} [multiple-dose], C_{tau} [multiple-dose], CL_{ss}/F [multiple dose], and V_z/F, C_{max}, T_{max}, C_{last}, T_{last}, λ_z, CL/F, and $t_{1/2}$.<br><br>**Pharmacodynamics:** Evaluated by PD markers including assessment of DNL, ANGPTL3, and a comprehensive serum lipid profile. Dose and/or exposure-response relationships for LXR inverse agonism by TLC-2716 will be evaluated.
**Statistical Methods:**<br><br>**Safety**:<br><br>Safety data will be listed by subject and treatment and summarized by treatment for each cohort. As appropriate, data from placebo-treated subjects will be pooled across Parts A, B, and/or C.<br><br>Treatment-emergent AEs will be summarized by system organ class (SOC) and preferred term (PT) using the most current version of the Medical Dictionary for Regulatory Activities (MedDRA®). All AEs and all treatment-related AEs will be listed by treatment and subject. The frequency of subjects who experience AEs will be summarized overall within the cohort and by treatment. All AEs will also be summarized by relationship to study drug and severity.<br><br>Listings of individual subject laboratory results will be provided. Selected laboratory data will be summarized by cohort at scheduled visits and for the corresponding change from Baseline (Day 1 pre-dose for SAD cohorts in Parts A and C; Day -1 for MAD cohorts in Parts B and C). The incidence of treatment-emergent, graded laboratory abnormalities will be summarized by cohort and treatment.<br><br>Individual data for physical examination findings, ECGs, and vital sign measurements will be listed by treatment and subject, and summarized overall within the cohort and by visit, as applicable.<br><br>**Pharmacokinetics**:<br><br>Plasma concentrations and PK parameters for TLC-2716 will be listed and summarized using descriptive statistics by treatment group in each cohort.<br><br>Dose proportionality will be obtained by comparing PK parameters of TLC-2716 across evaluated dose levels. Additional analyses such as accumulation ratio and time to steady-state may be conducted as appropriate.<br><br>**Pharmacodynamics:**<br><br>PD and PK/PD relationships will be explored.<br><br>**Sample Size**:<br><br>Due to its exploratory nature and no reliable variability estimation, no formal power or sample size calculations were used to determine the sample size for this study. Empirically, a sample size of 100-150 subjects (10 subjects per cohort, including 8 active and 2 placebo) was selected to provide an adequate characterization of safety assessments and PK and PD of TLC-2716.

This study will be conducted in accordance with the guidelines of Good Clinical Practices (GCPs) including archiving of essential documents.

GLOSSARY OF ABBREVIATIONS AND DEFINITION OF TERMS

ACC	acetyl-CoA carboxylase
AE	adverse event
ALT	alanine aminotransferase
ANGPTL3	angiopoietin-like protein-3
ANOVA	analysis of variance
ApoC3	Apolipoprotein C3
AST	aspartate aminotransferase
AUC	area under the concentration versus time curve
AUC_{0-24}	area under the plasma concentration-time curve from time 0 to 24 hours concentration
AUC_{0-last}	area under the plasma concentration-time curve from time 0 to the last measurable concentration
AUC_{inf}	area under the plasma/serum/PBMC concentration versus time curve extrapolated to infinite time, calculated as AUC_{0-last} + (Clast/λ_z)
AUC_{tau}	area under the plasma/serum/PBMC concentration versus time curve over the dosing interval
aVR	augmented Vector Right
BLQ	below the limit of quantitation
BMI	body mass index
BUN	blood urea nitrogen
CAD	coronary artery disease
CBC	complete blood count
CFR	Code of Federal Regulations
CK	creatine kinase
CL	systemic clearance
CL/F	apparent oral clearance
C_{last}	last observed quantifiable serum/plasma/PBMC concentration of the drug
C_{max}	maximum observed concentration of drug
CNS	central nervous system
COVID-19	coronavirus disease 2019
CRF	case report form(s)
CRO	contract (or clinical) research organization
C_{tau}	observed drug concentration at the end of the dosing interval
CV	cardiovascular
DDI	drug-drug interaction
DIO	diet-induced obese
DNL	*de novo* lipogenesis
EC_{50}	half-maximal effective concentration
ECG	electrocardiogram
eCRF	electronic case report form(s)

eSAE	electronic serious adverse event
ET	early termination
FASN	fatty acid synthase
FDA	(United States) Food and Drug Administration
FH	familial hypercholesterolemia
FIH	first-in-human
GCKR	glucokinase regulator
GCP	Good Clinical Practice (Guidelines)
HBV	hepatitis B virus
HCC	hepatocellular carcinoma
HCV	hepatitis C virus
HED	human equivalent dose(s)
HeFH	heterozygous familial hypercholesterolemia
HIV	human immunodeficiency virus
HMGCR	3-hydroxy-3-methylglutaryl-CoA reductase
HoFH	homozygous familial hypercholesterolemia
HOMA-IR	homeostatic model assessment for insulin resistance
IB	investigator’s brochure
IC_{50}	Half maximal inhibitory concentration
ICF	informed consent form
ICH	International Conference on Harmonization
IDOL	inducible degrader of low-density lipoprotein
IEC	independent ethics committee
IND	investigational new drug (application)
iPSC	induced pluripotent stem cell
IRB	institutional review board
IUD	intrauterine device
IV	intravenous
LAM	lactational amenorrhea method
LDL	low-density lipoprotein
LDL-C	low-density lipoprotein cholesterol
LDLR	low-density lipoprotein receptor
LLOQ	lower limit of quantitation
LPL	lipoprotein lipase
LXR	liver X receptor
MAD	multiple-ascending dose
MedDRA	medical dictionary for regulatory activities
MM	medical monitor
NAFLD	nonalcoholic fatty liver disease
NASH	nonalcoholic steatohepatitis

NHANES	US National Health and Nutrition Examination Surveys
NMR	nuclear magnetic resonance
NOAEL	no-observed-adverse-effect-level
NTCP	sodium-taurocholate cotransporting polypeptide
OATP	organic anion transporting polypeptides
PD	pharmacodynamics
PK	pharmacokinetic(s)
PT	preferred term
PT	prothrombin time
PTM	placebo-to-match
QD	once daily
QT	electrocardiographic interval between the beginning of the Q wave and termination of the T wave representing the time for both ventricular depolarization and repolarization to occur
SAD	single-ascending dose
SADR	serious adverse drug reaction
SAE	serious adverse event
SD	single dose
SD	Sprague Dawley
SHTG	severe hypertriglyceridemia
SOC	system organ class
SOP	standard operating procedure
SUSAR	suspected unexpected serious adverse reaction
$t_{1/2}$	an estimate of the terminal elimination half-life of the drug, calculated by dividing the natural log of 2 by the terminal elimination rate constant (λ_z)
TEAE	treatment-emergent adverse event
TG	triglycerides
T_{last}	time (observed time point) of C_{last}
TLC	The Liver Company, Inc.
t_{max}	the time (observed time point) of C_{max}
ULN	upper limit of normal
ULOQ	upper limit of quantitation
US	United States
VLDL	very low-density lipoprotein
V_{ss}	volume of distribution
ZDF	Zucker diabetic fatty
Λz	terminal elimination rate constant; estimated by linear regression of the terminal elimination phase of the log serum/plasma/PBMC concentration versus time curve of the drug
° C	degrees celsius
° F	degrees fahrenheit

1. INTRODUCTION

1.1. Background

TLC-2716 is a potent, first-in-class, small molecule, liver-targeted, inverse agonist of the nuclear hormone receptor, liver X receptor (LXR), and is under development for the treatment of severe dyslipidemias and nonalcoholic steatohepatitis (NASH).

1.1.1. Severe Dyslipidemias

1.1.1.1. Severe Hypertriglyceridemia (SHTG)

Hypertriglyceridemia (serum triglycerides [TG] > 150 mg/dL) is common among adults. According to the US National Health and Nutrition Examination Surveys (NHANES) from 2007 to 2014, the percentages of non-statin-treated adults with TG > 150 mg/dL, > 500 mg/dL, and > 1000 mg/dL were 25%, 1%, and < 1%, respectively (Tarugi, Bertolini et al. 2019). While no definition of SHTG has been uniformly accepted, the risk of acute pancreatitis increases progressively with serum TG > 500 mg/dL (Scherer, Singh et al. 2014). Hypertriglyceridemia results from an imbalance between the production and release of TG-rich lipoproteins from the liver (very low-density lipoprotein [VLDL]) and intestine (chylomicrons) and lipolytic removal of TG from these lipoproteins and their remnants. As stated above, SHTG is a cause of pancreatitis and is associated with an increased risk for atherosclerotic heart disease.

Management of SHTG focuses primarily on reducing serum TG concentrations by lifestyle modification (e.g., diet, exercise, alcohol reduction) and fibrates (e.g., fenofibrate, gemfibrozil) as first-line therapy. Adjunctive therapies include niacin, omega-3 fatty acids (e.g., icosapent ethyl), and statins. Due to the importance of lipoprotein lipase (LPL)-mediated clearance of TG, additional therapies aimed at decreasing the activity of proteins that inhibit LPL such as Apolipoprotein C3 (ApoC3) and angiopoietin-like protein-3 (ANGPTL3) are under investigation (Lang and Frishman 2019, Ahmad, Pordy et al. 2021).

In patients with SHTG, treatment with TLC-2716 is expected to reduce serum TG by two primary mechanisms: 1) inhibition of hepatic ANGPTL3 and ApoC3 leading to increased LPL activity and clearance of TG-rich lipoproteins (e.g., VLDL); and 2) inhibition of sterol regulatory element-binding protein 1c (SREBP1c) leading to reductions in *de novo* lipogenesis (DNL) and VLDL synthesis and release by the liver.

1.1.1.2. Familial Hypercholesterolemia (FH)

Familial hypercholesterolemia (FH) is a diagnosis which refers to individuals with very significantly elevated low-density lipoprotein cholesterol (LDL-C) and an increased risk for premature atherosclerotic heart disease if not sufficiently treated. Most commonly, individuals have heterozygous FH (HeFH), caused by a single DNA variant inherited from one affected parent. In rare cases, an individual can have homozygous FH (HoFH), a more severe form of disease caused by having two causal variants, one from each parent (Bouhairie and Goldberg

2015, Santos, Gidding et al. 2016, Defesche, Gidding et al. 2017). While HeFH is one of the most common genetic diseases, affecting approximately 1 in every 250 to 300 individuals, HoFH is rare, affecting approximately 1 in 300,000 individuals (Beheshti, Madsen et al. 2020).

People with FH have very high levels of LDL-C from birth, typically > 190 mg/dL in adults with HeFH and > 400 mg/dL in those with HoFH. In both populations, untreated hypercholesterolemia can lead to premature coronary artery disease (CAD), cerebrovascular disease, peripheral vascular disease, and other manifestations (e.g., aortic aneurysm, xanthelasma, xanthomas). Untreated, HeFH is associated with an approximately 10 to 20-fold increased risk for CAD; patients with HoFH experience severe CAD by their mid-twenties (Bouhairie and Goldberg 2015, Santos, Gidding et al. 2016, Defesche, Gidding et al. 2017).

Treatment of FH is focused on reducing LDL-C levels in order to decrease the risk for atherosclerotic heart disease. The goal of therapy is to lower LDL-C as much as is practical (intensive lowering), with management typically consisting of a lifestyle intervention (e.g., diet, exercise) and cholesterol-lowering medication including statins, ezetimibe, bile acid sequestrants, bempedoic acid, icosapent ethyl, or PCSK9 inhibitors (Raal, Hovingh et al. 2018, McGowan, Hosseini Dehkordi et al. 2019). In patients with HoFH, additional treatments are often required (e.g., evinacumab, lomitapide, LDL-C apheresis) (Cuchel, Meagher et al. 2013, Luirink, Determeijer et al. 2019, Raal, Rosenson et al. 2020, Underberg, Cannon et al. 2020).

In patients with FH, treatment with TLC-2716 is expected to inhibit hepatic inducible degrader of the LDL receptor [LDLR] (IDOL), thereby increasing levels of LDLR in hepatocytes, resulting in lowering of serum LDL-C concentrations due to increased hepatic uptake by LDLR. Moreover, reduced hepatic expression of 3-hydroxy-3-methylglutaryl-CoA reductase (HMGCR), the rate-limiting enzyme in cholesterol biosynthesis, has been demonstrated in pre-clinical models of LXR inhibition, providing an additional potential mechanism for LDL-C lowering in these patients.

1.1.2. Nonalcoholic Steatohepatitis (NASH)

In addition to patients with severe dyslipidemias, the potential benefits of TLC-2716 on lipid-lowering, driven primarily by activity within the liver and inhibition of hepatic DNL, are potentially beneficial in patients with NASH. Due to the emerging epidemics of obesity, metabolic syndrome, and diabetes, the prevalence of chronic liver disease, specifically nonalcoholic fatty liver disease, is increasing globally. The prevalence of NAFLD is approximately 30% globally (Ong and Younossi 2007, Vernon, Baranova et al. 2011). Nonalcoholic steatohepatitis (NASH), the aggressive variant of NAFLD, is characterized by hepatic inflammation and hepatocellular ballooning, with or without fibrosis, is present in approximately 25% of patients with NAFLD. The unmet medical need in NASH is significant and growing; thus far, no therapies have been approved. Current management consists of lifestyle modification (e.g., diet, exercise, minimization of alcohol intake) and optimization of comorbid conditions including dyslipidemia and diabetes mellitus.

A central feature of NASH is the accumulation of lipids within hepatocytes, due in part, to increased hepatic DNL. Increased DNL leads to the generation of fatty acid signaling molecules

with pro-inflammatory and pro-fibrotic effects (lipotoxicity) (Neuschwander-Tetri 2010). The importance of increased DNL to the pathogenesis of NASH has been confirmed in pre-clinical models and human studies employing stable isotope tracers, and are supportive of the development of lipogenesis inhibitors, many of which are currently in development (e.g., inhibitors of acetyl-CoA carboxylase [ACC], fatty acid synthase [FASN], and stearoyl-CoA desaturase-1 [SCD-1]) (Lambert, Ramos-Roman et al. 2014, Lawitz, Coste et al. 2018).

Based on the importance of increased DNL in the pathogenesis of NASH, TLC-2716 is a promising potential therapy, from both hepatic and metabolic perspectives. The liver X receptor is a key regulator of SREBP1c, the master transcriptional regulator of DNL. Inhibition of LXR in the liver is expected to downregulate SREBP1c and downstream lipogenic genes including ACC1, FASN, and SCD1, thereby reducing hepatic steatosis, and potentially in turn, inflammation and fibrosis (Griffett, Solt et al. 2013, Griffett, Welch et al. 2015, Hambruch, Deuschle et al. 2017). Indeed, such effects have been observed with short-term TLC-2716 treatment in multiple dysmetabolic rodent models of NASH. In mice and in Sprague Dawley (SD) and Zucker Diabetic Fatty (ZDF) rats, all fed a high-fat diet (HFD), treatment with TLC-2716 for 2-3 weeks led to decreases in liver and plasma TG, reductions in hepatic expression of lipogenic genes (e.g., SREBP1c, ACC1, FASN, SCD1), and improvements in plasma cholesterol.

In addition to these potential hepatic benefits of LXR inhibition, treatment with TLC-2716 may address the metabolic dysfunction characteristic of NASH; specifically, dyslipidemia and insulin resistance. For example, in a study of HFD-fed ZDF rats, treatment with TLC-2716 for 4 weeks led to improvements in plasma TG, glucose, and hepatic insulin sensitivity, as measured by the Homeostatic Model Assessment for Insulin Resistance (HOMA-IR). These effects are likely mediated by increased glucose utilization in peripheral tissues (e.g., muscle) and/or preservation of insulin-producing β-cells in the pancreas.

Overall, in the context of high unmet need amongst patients with severe dyslipidemias and NASH, and on promising pre-clinical data, the clinical development program for TLC-2716 will include patients with SHTG, FH, and NASH.

1.2. TLC-2716

1.2.1. General Information

TLC-2716 is a potent, first-in-class, small molecule, liver-targeted, inverse agonist of the nuclear hormone receptor, LXR. The liver X receptors (LXRs) — LXRα and LXRβ — are members of the nuclear hormone receptor superfamily of ligand-activated transcription factors and key transcriptional regulators of systemic lipid metabolism (Janowski, Willy et al. 1996, Janowski, Grogan et al. 1999). The endogenous ligands for LXRs are oxidized forms of cholesterol (oxysterols) that increase coordinately with intracellular cholesterol levels, thus allowing these receptors to function as sensors to maintain cholesterol homeostasis throughout the body via a feedforward mechanism. In addition, LXRs play a central role in hepatic DNL and in the regulation of plasma TG by upregulating key hepatic lipogenic genes. In pre-clinical models, inverse agonism (functionally, inhibition) of LXR in the liver by TLC-2716 has demonstrated

reductions in hepatic lipogenic genes as well as hepatic and plasma TG, and plasma cholesterol, supporting the development of TLC-2716 for the treatment of severe dyslipidemias and NASH.

For further information on TLC-2716, refer to the current investigator's brochure (IB).

1.2.2. Nonclinical Pharmacology

Based on biochemical binding assays, cellular mammalian two-hybrid interaction assays evaluating nuclear receptor co-repressor recruitment, cellular reporter assays, and an Upcyte® hepatocytes lipid droplet assay, TLC-2716 is a potent and selective inverse agonist (inhibitor) of LXRα and LXRβ ($EC_{50}/IC_{50} \leq 15$ nM). In addition, *in vitro* evidence of the biological activity of TLC-2716 was confirmed in induced pluripotent stem cell (iPSC)-derived human liver organoids (HLOs), which demonstrated profound inhibition of lipogenic genes (e.g., SREBP1c, ACC1, FASN, SCD1) and reductions in hepatocyte steatosis when exposed to TLC-2716. These changes were particularly pronounced in HLOs with genetic risk variants (glucokinase regulator [GCKR] loss of function) associated with higher DNL, providing the rationale for exploring the effects of TLC-2716 in NASH patients with increased DNL.

In vivo data in rodents support rapid hepatocyte uptake of TLC-2716, likely mediated by organic anion transporting polypeptides (OATP) and sodium taurocholate cotransporting polypeptide (NTCP) transporters. *In vivo* data supporting the liver-specific activity of TLC-2716 includes reductions in serum markers of liver-specific inhibition of DNL, without systemic inhibition of LXR-related gene expression in peripheral whole blood assays. Further, the effects of repeat oral dosing of TLC-2716 have been evaluated in multiple dysmetabolic rodent models including the diet-induced obese (DIO) mouse and HFD-fed Zucker diabetic fatty (ZDF) and Sprague-Dawley (SD) rats. Across these models, TLC-2716 exhibited profound reductions in liver and plasma TG, improvements in plasma cholesterol and insulin sensitivity, and potent inhibition of the expression of genes involved in DNL and the homeostasis of TG and cholesterol.

1.2.3. Nonclinical Pharmacokinetics (PK)

Absorption, distribution, metabolism, and elimination of TLC-2716, as well as the potential for drug-drug interactions, were assessed *in vitro* and *in vivo* in preclinical models. The pharmacokinetic (PK) profile indicates limited systemic exposure, with undetectable plasma levels 2-4 hours after dosing, and high levels in the liver consistent with rapid transporter-mediated uptake into hepatocytes.

TLC-2716 is a lipophilic, small molecule, LXR inverse agonist with a high binding affinity to plasma proteins (> 99%) in humans, mice, rats, dogs, and monkeys. Following intestinal absorption into the portal vein, TLC-2716 is highly extracted by the liver (92% hepatic extraction ratio in rats). Consistent with high liver loading, systemic exposure of TLC-2716 was generally low following oral administration in multiple species. In rodents, TLC-2716 was predominantly distributed to the liver and intestine; brain distribution data suggest that little or no TLC-2716-derived compounds cross the blood-brain barrier. *In vitro*, TLC-2716 was stable in primary hepatocytes from human, mouse, and dog. Together with the metabolites observed in

livers from preclinical species *in vivo*, the biotransformation pathway of TLC-2716 appears to involve oxidation, deamination, desulfation, glucuronidation, and taurine conjugation.

In *in vitro* studies, TLC-2716 was not a substrate of CYP1A2, CYP2B6, CYP2C8, CYP2C9, CYP2C19, CYP2D6, or CYP3A5, however, it was a modest substrate of CYP3A4 suggesting that co-administration of CYP3A4 inhibitors or inducers may affect the exposure of TLC-2716. TLC-2716 did not inhibit CYP1A2, CYP2C19, or CYP2D6, but demonstrated weak to modest inhibition of CYP2B6, CYP2C8, CYP2C9, and CYP3A4. In primary human hepatocytes, TLC-2716 induced CYP2B6 and CYP3A4 (> 2-fold relative to control), and therefore, may alter the exposures of co-administered substrates of these CYP enzymes *in vivo*. Finally, inhibition of P-gp, BCRP, OATP1B1, and OATP1B3 by TLC-2716 was observed *in vitro* at 10 μM. While these findings suggest that TLC-2716 may alter the exposure of concomitant medications (and vice versa), the unique PK profile of TLC-2716, characterized by minimal systemic exposure and high protein binding, may reduce the potential for clinically relevant drug-drug interactions. Additional studies will be conducted to evaluate the clinical relevance of these findings.

1.2.4. Nonclinical Toxicology

The nonclinical toxicity profile of TLC-2716 has been assessed in repeat-dose toxicity studies of up to 4 weeks duration in mice and monkeys. These species were selected based on their suitable PK profiles and similarity of their *in vitro* metabolic profiles to that of humans. In mice, treatment with TLC-2716 at dose levels of 4, 20, and 80 mg/kg/day for 28 days was well tolerated with only transient and slight body weight loss (< 3.5%) noted at 80 mg/kg/day. There were no test article-related changes in food and water consumption, ophthalmology, clinical chemistry, hematology, or urinalysis parameters. Similarly, no changes in organ weight or macro- or microscopic findings were attributed to TLC-2716. Therefore, the no-observed-adverse-effect-level (NOAEL) was considered to be 80 mg/kg/day.

The potential toxicity of TLC-2716 has been assessed in male and female cynomolgus monkeys for 2- and 4-week durations. In the 4-week study, animals (n = 3/sex/group) were administered vehicle control or TLC-2716 at dose levels of 0, 1, 5, or 15 mg/kg QD by oral gavage. Overall, oral administration of TLC-2716 at all dose levels for 28 days was well tolerated. Only reversible changes in stools (soft or watery) were observed occasionally in all dose groups, but predominantly at the 15 mg/kg/day dose in males and the 5 and 15/mg/kg/day doses in females. No test article-related changes in body weight, food consumption, body temperature, ophthalmology, clinical chemistry, hematology, coagulation, or urinalysis parameters were observed. Similarly, there were no changes in organ weights or macro- or microscopic findings attributed to the administration of TLC-2716. Therefore, the NOAEL was considered to be 15 mg/kg/day. Of note, a single death was observed in the low dose (1 mg/kg/day) group on Day 5 of the study. This death, which was attributed to renal insufficiency and urinary stasis, was considered not related to TLC-2716. At the end of the study, no findings in clinical chemistry, urinalysis, or histologic parameters suggestive of TLC-2716-induced alterations on the renal system were observed.

Please refer to the TLC-2716 IB for additional details regarding nonclinical pharmacology, PK, and toxicology.

1.2.5. Clinical Trials of TLC-2716

No prior clinical studies involving TLC-2716 have been conducted.

1.3. Rationale for the Current Study

This Phase 1 study will evaluate the administration of TLC-2716 in humans for the first time. The objectives of this first-in-human (FIH) study are to evaluate, in a stepwise fashion, the safety, tolerability, PK, and pharmacodynamics (PD) of single- and multiple-ascending doses of TLC-2716. The results from this study, including safety and PD data across the expected therapeutic range of drug exposures planned for future studies, will form the basis for further evaluation of TLC-2716 and dose selection for a subsequent Phase 2 trial in patients with SHTG, FH, and NASH. In the context of evaluating safety, tolerability, PK, and PD, the latter of which includes changes in serum lipids, potentially impacted by diet, this trial will be conducted in an inpatient setting (confined Phase 1 unit), allowing for consistency in diet and timing of sample collections. To remove potential confounding effects of comorbid disease and/or therapies in patients with metabolic disease, healthy subjects will be recruited for this trial. Of note, in multiple-dose cohorts, a subset of subjects with mild to moderately elevated serum TG and/or LDL will be identified, facilitating an early evaluation of potential lipid-lowering effects of TLC-2716 in otherwise healthy individuals.

1.3.1. Rationale for the Study Design

The objective of this FIH study is to evaluate the safety, tolerability, PK, and PD of TLC-2716; to support the characterization of the clinical pharmacology profile of TLC-2716; and to determine optimal doses of TLC-2716 for future clinical studies.

This study will proceed in three parts, with the first part (Part A) evaluating the safety, tolerability, PK, and PD of escalating single oral doses of TLC-2716 or PTM in the fed state over 5 cohorts. Subjects in Part A will be confined for a minimum of 72 hours post single-dose administration (through Day 4) to monitor relevant safety and tolerability parameters prior to discharge, and subsequently evaluated in-clinic 14 days after dosing. To maximize subject safety, in SAD cohorts in Part A, two sentinel subjects (one randomized to TLC-2716 and one to placebo-to-match [PTM]) will be dosed and safety and tolerability data collected through Day 2 will be evaluated, prior to dosing of the remaining subjects in each cohort.

The second part (Part B) of the study is similarly designed to evaluate the safety, tolerability, PK, and PD of escalating multiple doses (14 days) of TLC-2716. The third part (Part C) of the study is designed to be adaptive (based on initial data from Part A and/or Part B, if applicable), to evaluate the safety, tolerability, PK, and PD of TLC-2716, with adaptive dose selection (up to 50 mg total daily dose), administered as a single dose or multiple doses once or twice daily over 14 days, in a fed or fasting state. In all cohorts, throughout the periods of subject confinement, safety, PK, and PD assessments will be conducted at specified intervals, and then repeated at an in-clinic follow-up visit two weeks after the last dose.

Within Part A and SAD cohorts in Part C, initiation of single-dose administration at escalating dose levels will be determined by evaluation of cumulative safety data, and available and relevant PK and PD data up to and including Day 4, following single-dose administration of the previous dosing cohort. Similarly, in MAD cohorts in Parts B and C, initiation of multiple-dose administration at a higher dose level will be determined by evaluation of safety and available PK and PD data from all subjects enrolled through Day 14 of the previous dosing cohort. Additionally, in adaptive cohorts in Part C, sentinel dosing will be utilized in any cohort wherein the total daily dose to be administered is greater than has previously been evaluated. In these multiple-dose cohorts, to support a comprehensive evaluation of the relationship between TLC-2716 PK and PD, all subjects regardless of treatment assignment will be administered placebo capsule(s) on Day -1 to characterize the baseline daily variation of PD markers for subsequent comparison with changes that occur during dosing of TLC-2716 or PTM from Day 1 through 14. Additional details regarding dose escalation criteria are provided in Section 3.2.

1.3.2. Rationale for Dose Selection

In this FIH study, oral doses of TLC-2716 ranging from 0.5 mg to a maximum of 50 mg per day (a 100-fold dose range) will be evaluated in healthy subjects. These doses were selected to provide predominantly liver exposure and minimize the potential for adverse effects mediated by systemic exposure (e.g., impact on reverse cholesterol transport). A starting dose of 0.5 mg and a maximum dose of 50 mg provide 672- and 6.7-fold safety margins based on the NOAEL observed in the 4-week GLP toxicity study in monkeys, the most sensitive preclinical species evaluated (Table 1). Based on pre-clinical pharmacology data, it is expected that doses at the lower end of this range will demonstrate efficacy for relevant clinical parameters based on the preferential liver distribution of TLC-2716. For example, in the DIO mouse and HFD-fed ZDF rat models, a daily TLC-2716 dose of 1 mg/kg administered for 2-3 weeks led to significant reductions in liver TG, plasma TG, and cholesterol concentrations. Based on allometric scaling for mice and rats, this dose is equivalent to a human equivalent dose (HED) of approximately 6 to 11 mg per day in a 70-kg human.

Table 1. Estimated Dose Margins Following Oral Administration of TLC-2716 Based on 4-week, GLP Chronic Toxicology Studies

Species	Duration of Dosing (Days)	NOAEL (mg/kg/day)	AUC_{0-24} (ng.h/mL)	Human Equivalent NOAEL (mg)[a]	Dose Margins[b]		
					0.5 mg	20 mg	50 mg
Mouse	28	80	149.13 (male)<br>162.11 (female)	455	910	22.8	9.1
Monkey	28	15	51.08 (male)<br>74.09 (female)	336	672	16.8	6.7

AUC_{0-24} = area under the plasma concentration-time curve from time 0 to 24 hours concentration; FIH = first-in-human; HED = human equivalent dose; NOAEL = no-observed-adverse-effect-level

a Estimated by body surface area allometric scaling and human body weight of 70 kg. Monkey is considered the most sensitive species.

b Based on dose levels proposed in Phase 1 of 0.5 mg (lowest dose in Parts A and B), 20 mg (highest dose in Parts A and B), or 50 mg (maximum total daily dose in Part C Adaptive cohorts).

1.4. Risk/Benefit Assessment for the Study

Potential risks of study participation include unknown AEs, general risks associated with frequent clinic visits and laboratory blood draws, and the associated pain and discomfort of phlebotomy. Strategies to mitigate these risks include sentinel dosing (as described above) and close monitoring of lab values and AEs. Moreover, parameters for discontinuation of the study drug due to AEs will be well-defined and closely followed.

There is no direct benefit by participation in this study; however, data from this study will support the development of TLC-2716 for the treatment of severe dyslipidemias and NASH. Potential benefits may include the subject's contribution to understanding the safety, tolerability, and pharmacology of single- and multiple- escalating doses of TLC-2716.

Considering the above, the benefit-risk balance for this study is considered positive.

1.5. Compliance

This study will be conducted in compliance with this clinical study protocol, Good Clinical Practice (GCP), and all applicable regulatory requirements.

2. OBJECTIVES

The primary objectives of this study are as follows:

- To evaluate the safety and tolerability of escalating single and multiple doses of TLC-2716
- To characterize the single- and multiple-dose pharmacokinetics (PK) of TLC-2716 and its metabolite(s)

The exploratory objectives of this study are as follows:

- To evaluate the pharmacodynamics (PD) of liver X receptor (LXR) inverse agonism by TLC-2716 as measured by metabolic parameters including fasting lipids and serum biomarkers of *de novo* lipogenesis (DNL)
- To characterize TLC-2716 dose and/or exposure-response relationships for PD markers
- To evaluate exploratory biomarkers

3. STUDY DESIGN

3.1. Study Design Description

This protocol describes a single- and multiple-ascending dose FIH Phase 1 study to evaluate the safety, tolerability, PK, and PD of TLC-2716 in healthy adult subjects.

The study will be conducted at a single Phase 1 center in New Zealand. A total of up to 150 subjects will be enrolled, including 100 subjects in Parts A and B, and up to 50 subjects in adaptive cohorts in Part C. Within each cohort, 10 subjects will be randomized, including 8 subjects randomized to TLC-2716 and 2 subjects to PTM. In single-dose cohorts in Parts A and in any Part C cohort evaluating a higher total daily dose than previously evaluated, safety and tolerability data collected through Day 2 from two sentinel subjects, one randomized to TLC-2716 and one to PTM, will be evaluated prior to dosing of the remaining subjects in each cohort.

An approximately even distribution of healthy male and non-pregnant, non-lactating female subjects 18 to 55 years of age, inclusive, will be enrolled in the study. Subjects may not participate in more than one cohort of the study. Following the completion of Screening and admission assessments, eligible subjects will be enrolled on Day -1.

The duration of dosing will be 1 day for the SAD cohorts in Part A and C, and 15 days for the MAD cohorts in Parts B and C, including PTM dosing on Day -1 for all subjects in MAD cohorts.

The overall study duration, post-screening, will be up to 18 days for Part A, and up to 32 days for Parts B and C.

This study will proceed in 3 parts, with progression within and between parts governed by reviews of safety and any available and relevant PK and/or PD data, and the application of stopping rules. The sponsor, in consultation with the investigator, may choose not to initiate any or all adaptive cohorts in Part C if deemed unnecessary based on observations in Parts A and B. The overall study design and the design of each part are described below, and the study schemas are presented in Figure 1 for SAD cohorts in Parts A and C, and Figure 2 for MAD cohorts in Parts B and C.

3.1.1. Part A: Single-Ascending Dose [SAD] (Cohorts 1-5)

Part A will evaluate the safety, tolerability, PK, and PD of single-ascending doses of oral TLC-2716 or placebo under fed conditions. Part A will proceed in up to 5 dose-escalation cohorts and will be governed by a review of safety, tolerability, and any available and relevant PK and/or PD data, and study-specific stopping criteria. Planned study treatments within each cohort are provided in Table 2.

- Within each cohort, 10 unique subjects will be randomized 4:1 to receive either blinded TLC-2716 (N = 8) or PTM (N = 2). All study drugs in Part A will be administered in the morning following a standard meal.
- At the initiation of each cohort in Part A, prior to randomization of the entire cohort, two sentinel subjects will be randomized, one to TLC-2716 at the dose to be evaluated in the planned cohort, and one to corresponding PTM. Enrollment and randomization of the remaining eight subjects in each cohort will be determined upon evaluation of all safety and tolerability data through Day 2 for these two sentinel subjects.
- For cohorts 2-5, initiation of single-dose administration will be permitted after evaluation of cumulative blinded safety data, and any relevant and available PK and/or PD data up to and including Day 4, following single-dose administration of the previous cohort.

Table 2. Treatments to be Administered in Cohorts 1-5

Cohort	Single Ascending Doses (Day 1)
1	0.5 mg TLC-2716 (1 x 0.5 mg) or PTM capsule SD, fed
2	2 mg TLC-2716[a] (1 x 2 mg) or PTM capsule SD, fed
3	6 mg TLC-2716[a] (3 x 2 mg) or PTM capsules SD, fed
4	12 mg TLC-2716[a] (2 x 5 mg; 1 x 2 mg) or PTM capsules SD, fed
5	20 mg TLC-2716[a] (4 x 5 mg) or PTM capsules SD, fed

PTM = placebo-to-match; SD = single dose
a The planned doses may be modified based on the observed safety, tolerability, and any relevant and available PK and/or PD data. Any modified dose will be within 3-fold of a dose previously tested.

A schematic of the activities within Part A cohorts is shown in Figure 1. Adaptive SAD cohorts in Part C will follow the same study schema.

Figure 1. Study Schema – Part A, SAD Cohorts 1-5

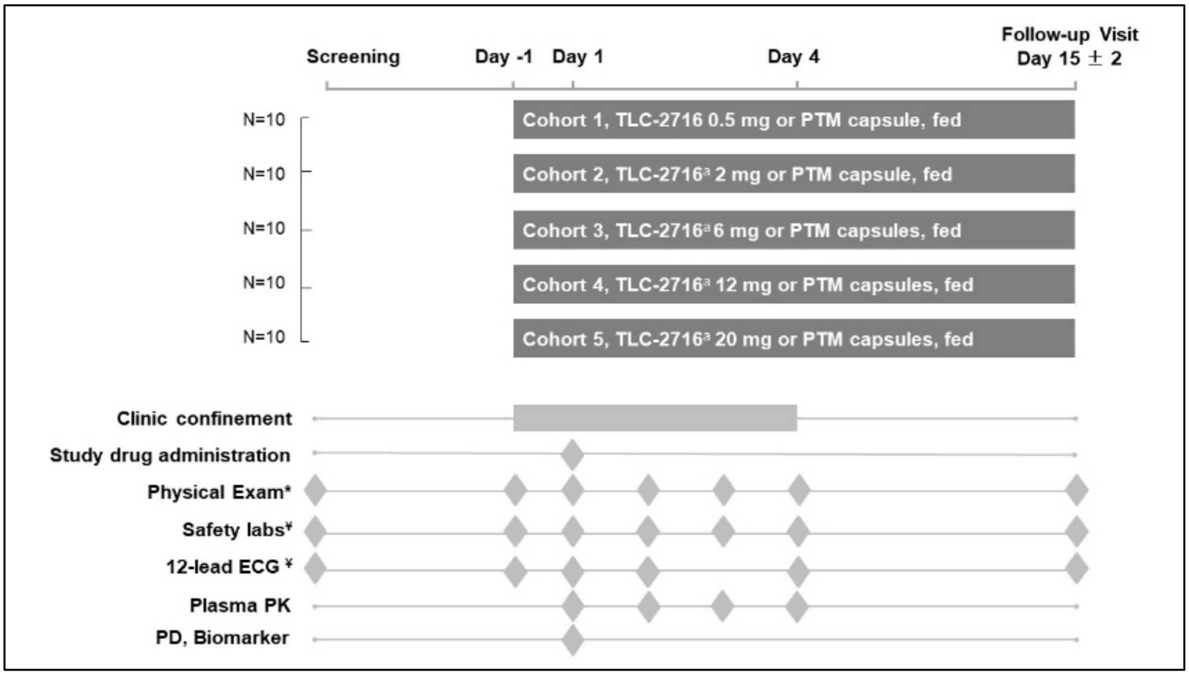

For each cohort, subjects will be randomized to 8 active and 2 placebo-to-match.

Part C (Cohorts 11-15): Adaptive SAD Cohorts will follow the same schema as in Part A and Adaptive MAD Cohorts will follow the same schema as Part B.

a The planned doses may be modified based on the observed safety, tolerability, and any relevant and available PK and/or PD data. Any modified dose will be within 3-fold of a dose previously tested.

* Complete PE at Screening, Day -1, Day 4, and Follow-up visit at Day 15 ± 2; symptom-driven exam performed at other time points indicated.

¥ Safety labs on Day 1 to occur 12 hours after study drug dosing; 12-lead ECG on Day 1 to occur 2 hours after study drug dosing.

3.1.2. Part B: Multiple-Ascending Dose [MAD] (Cohorts 6-10)

Part B will evaluate the safety, tolerability, PK, and PD of multiple-ascending doses of oral TLC-2716 or placebo under fed conditions. Part B will proceed in up to 5 dose-escalation cohorts and will be governed by a review of safety, tolerability, and any available and relevant PK and/or PD data, and study-specific stopping criteria. Planned study treatments within each cohort are provided in Table 3.

- Randomized, blinded (sponsor-unblinded), placebo-controlled, multiple-ascending doses of TLC-2716 in healthy subjects. An attempt will be made to enroll at least 3 subjects in each cohort with Screening TG ≥ 150 mg/dL and/or low-density lipoprotein cholesterol (LDL-C) ≥ 130 mg/dL.
- Within each cohort, 10 unique subjects will be randomized 4:1 to receive either blinded TLC-2716 (N = 8) or PTM (N = 2) QD for 14 days.
- Part B (Cohort 6) may be initiated after the evaluation of cumulative safety, tolerability, and any relevant and available PK and/or PD data from all subjects enrolled in Cohort 3 in Part A (up to and including Day 4).

- For Cohorts 7-10, dosing may be initiated after evaluation of cumulative safety, tolerability, and any relevant and available PK and/or PD data from all subjects enrolled in the previous cohort in Part B through Day 14.
- All study drugs in Part B will be administered in the morning following a standard meal.

Table 3. Treatments to be Administered in Cohorts 6-10

Cohort	Day -1	Multiple Ascending Doses (Days 1-14)
**6**	PTM capsule SD	0.5 mg TLC-2716 (1 x 0.5 mg) or PTM capsule, fed
**7**	PTM capsule SD	2 mg TLC-2716[a] (1 x 2 mg) or PTM capsule, fed
**8**	PTM capsules SD	6 mg TLC-2716[a] (3 x 2 mg) or PTM capsules, fed
**9**	PTM capsules SD	12 mg TLC-2716[a] (2 x 5 mg; 1 x 2 mg) or PTM capsules, fed
**10**	PTM capsules SD	20 mg TLC-2716[a] (4 x 5 mg) or PTM capsules, fed

PTM = placebo-to-match; SD = single dose

a The planned doses may be modified based on the observed safety, tolerability, and any relevant and available PK and/or PD data. Any modified dose will be within 3-fold of a dose previously tested.

3.1.3. Part C: Adaptive SAD and/or MAD (Cohorts 11-15)

Part C includes optional, adaptive cohorts to evaluate the safety, tolerability, PK, and PD of single- and/or multiple-ascending doses of oral TLC-2716 or placebo under fasted or fed conditions. Based on available safety, PK, and/or PD data generated in Part A and Part B, as applicable, doses for Part C (Cohorts 11-15) will be chosen, up to a total daily dose of 50 mg. Planned study treatments within each cohort are provided in Table 4.

Part C will proceed in up to 5 dose-escalation cohorts and will be governed by a review of safety, tolerability, and any available and relevant PK and/or PD data generated from previous Part A, B, and/or C cohorts, as applicable.

- Randomized, blinded (sponsor-unblinded), placebo-controlled, single- and/or multiple-ascending doses of TLC-2716 with adaptive dose selection in healthy subjects. An attempt will be made to enroll at least 3 subjects in each cohort with Screening TG ≥ 150 mg/dL and/or low-density lipoprotein cholesterol (LDL-C) ≥ 130 mg/dL.
- Within each cohort, 10 unique subjects will be randomized 4:1 to receive up to a total daily dose of 50 mg TLC-2716 (N = 8) or PTM (N = 2) for either 1 day (as in Part A) or 14 days (as in Part B).

The frequency of dosing (QD or BID in MAD cohorts) and fasting versus fed status (in SAD or MAD cohorts) may also be modified versus Parts A and B. If dosing is administered in the fed state, standard meal conditions may also be modified at sponsor discretion. QD doses will be administered in the morning following a standard meal, and BID doses will be administered after a standard morning meal and evening snack, with the second dose administered 12 hours

(± 10 minutes) after the morning dose. Once determined, dose level, duration of dosing, frequency of dosing, and meal conditions will remain consistent within a cohort. For any MAD cohort in Part C with BID dosing, both morning and evening doses must be administered on Day 14.

Multiple-dose, adaptive cohorts in Part C may be initiated in parallel with previous cohorts if the total daily dose under evaluation is at or below a dose already evaluated in Part B. If the total daily dose in any cohort is greater than has been previously evaluated, then sentinel dosing including one subject randomized to placebo and one subject to TLC-2716 will be included. Randomization of the remaining eight subjects in the cohort will be based on evaluation of all safety and tolerability data through Day 2 for these two sentinel subjects. If doses chosen in 2 or more adaptive cohorts exceed the dose evaluated in a previous cohort in Part B, these cohorts will be conducted in a staggered manner (lowest dose first), with the same stopping rules applied (Section 3.3).

Table 4. Treatments to be Administered in Cohorts 11-15

Cohort	Day -1[a]	Single Dose (Day 1) or Multiple Doses (Days 1-14)
**11-15**	PTM capsule(s) QD or BID	≤ 50 mg total daily dose TLC-2716 or PTM, administered QD or BID, fasting or fed

BID = twice daily; PTM = placebo-to-match; QD = once daily
a Dosing of PTM on Day -1 will only be performed in multiple-dose adaptive cohorts (as in Part B).

A schematic of the activities within MAD cohorts in Parts B and C is shown in Figure 2.

Figure 2. Study Schema – Part B, MAD Cohorts 6-10

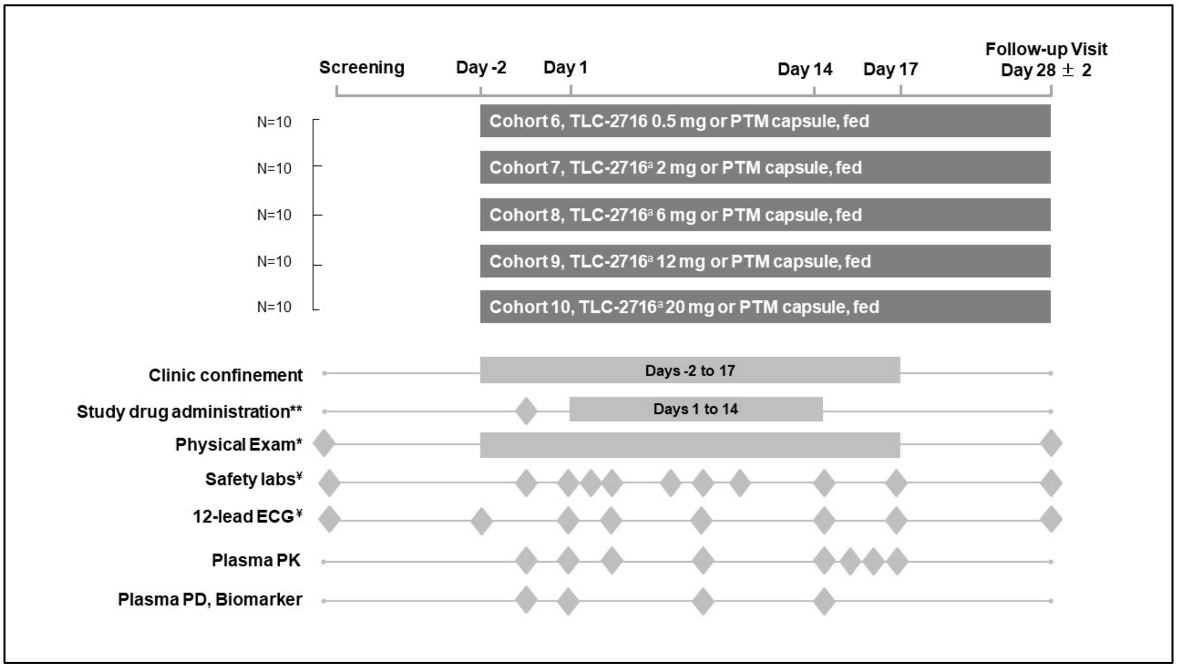

For each cohort, subjects will be randomized to 8 active and 2 placebo-to-match.

Part C (Cohorts 11-15): Adaptive SAD Cohorts will follow the same schema as in Part A and Adaptive MAD Cohorts will follow the same schema as Part B.

a The planned doses may be modified (reduced) based on the observed safety, tolerability, and any relevant and available PK and/or PD data. Any modified dose will be within 3-fold of a dose previously tested.

* Complete PE at Screening, Day -2, Day 17, and Follow-up visit on Day 28 ± 2; symptom-driven PE on all other days of confinement.

** All multiple-dose subjects in Part B and C will be administered PTM on Day -1.

¥ Safety labs on Day 1 to occur 12 hours after study drug dosing; 12-lead ECG on Day 1 to occur 2 hours after study drug dosing.

3.2. Dose Escalation Criteria

SAD Cohorts in Parts A and C

At the initiation of each single-dose cohort in Part A and in any single-dose cohort in Part C evaluating a higher total daily dose of TLC-2716 than previously evaluated, two sentinel subjects will be randomized, one to TLC-2716 at the dose to be evaluated in the planned cohort, and one to corresponding PTM. Enrollment and randomization of the remaining eight subjects in each cohort will be determined upon evaluation of all safety and tolerability data through Day 2 for these two sentinel subjects.

Initiation of single-dose administration at a higher dose will be determined by evaluation of safety and any relevant and available PK and/or PD data through Day 4 for all subjects enrolled in the previous dosing cohort.

MAD Cohorts in Part B

Dosing in Cohort 3 of the SAD portion of the study (Part A) will be completed prior to proceeding with the MAD portion of the study. Initiation of multiple dosing within Cohort 6 will be determined by a review of the cumulative safety, and any relevant and available PK and/or PD data through Day 4 of Cohort 3 in Part A.

Initiation of subsequent multiple-dose cohorts at a higher dose will be determined upon evaluation of all safety data, and any relevant and available PK and/or PD data through Day 14 from all subjects enrolled in the previous multiple-dose cohort.

In both Parts A and B, escalation to a dose higher than previously studied may occur only in the absence of dose-limiting toxicity and/or not meeting any prespecified stopping criteria.

Adaptive MAD Cohorts in Part C

Based on safety and available PK and/or PD data from cohorts in Part A and Part B (if applicable), TLC-2716 doses for Part C may be chosen up to a total daily dose of 50 mg. If the total daily dose in any cohort in Part C is greater than has been evaluated in any prior cohort, then sentinel dosing including one subject randomized to placebo and one subject randomized to TLC-2716 will be completed. Randomization of the remaining eight subjects in the cohort will be determined based on evaluation of all safety and tolerability data through Day 2 for these two sentinel subjects. Additionally, if doses chosen in two or more adaptive cohorts exceed the dose evaluated in a previous cohort, those cohorts will be conducted in a staggered manner (lowest dose first), with the same stopping rules applied. Part C cohorts may be initiated in parallel with cohorts in Part B if the total daily dose under evaluation is at or below a dose already evaluated.

The sponsor, in consultation with the investigator, may choose not to initiate any or all adaptive cohorts if deemed unnecessary.

3.3. Protocol-Specific Stopping Criteria

3.3.1. Subject Study Drug Discontinuation Criteria

The severity of AEs will be graded using the Common Terminology Criteria for Adverse Events version 5.0 (CTCAE v5.0) (Appendix 4).

Study drug must be discontinued in subjects who experience any of the following:

- A confirmed, treatment-emergent, treatment-related, serious AE (SAE) or ≥ Grade 3 AE
- Any ≥ Grade 3 clinically significant laboratory abnormality (confirmed by repeat testing) as defined by the Common Terminology Criteria for Adverse Events (CTCAE) v5.0 for Severity of Adverse Events and Laboratory Abnormalities
- Symptoms of drug-related hepatotoxicity, and/or ALT or AST > 5 x ULN, or ALT > 3 x ULN and total bilirubin > 2 x ULN or INR > 1.5, confirmed by immediate repeat testing

Study drug and/or study procedures may also be discontinued in the following instances:

- Intercurrent illness that would, in the judgment of the investigator, affect assessments of clinical status to a significant degree
- Unacceptable toxicity, as defined in the toxicity management section of the protocol, or toxicity that, in the judgment of the investigator, compromises the ability to continue study-specific procedures or is considered not to be in the subject's best interest
- Subject request to discontinue for any reason
- Subject noncompliance
- Pregnancy during the study (Refer to Appendix 3)
- Investigator discretion
- Discontinuation of the study at the request of TLC, regulatory agency, or an Institutional Review Board (IRB)/Independent Ethics Committee (IEC)

3.3.2. Study-Specific Stopping Criteria

Dose escalation or study drug dosing of a cohort will be suspended and based on a full review of the clinical data by the TLC medical monitor (MM) or designee, and in discussion with the investigator, may be halted when:

- 1 subject dosed with TLC-2716 experiences a treatment-emergent SAE deemed possibly related to study drug in the opinion of the investigator
- 2 subjects dosed with TLC-2716 within 1 cohort experience elevations in liver biochemistry tests of ALT and/or AST > 5 x the ULN, or ALT > 3 x ULN and total bilirubin > 2 x ULN or INR > 1.5, confirmed by immediate repeat testing
- 2 or more subjects dosed with TLC-2716 experience the same ≥ Grade 3 AE deemed possibly related to the study drug in the opinion of the investigator
- 2 or more subjects dosed with TLC-2716 experience a clinically significant ≥ Grade 3 laboratory abnormality of similar nature (confirmed on repeat testing) and possibly related to the study drug in the opinion of the investigator
- The number and/or severity of AEs justify study discontinuation
- The sponsor requests study discontinuation

Decisions to reinitiate the study will be made in consultation with the sponsor and pending a comprehensive safety review.

3.4. Duration of Dosing

The duration of dosing for each part will be as follows:

Part A (SAD): 1 day

Part B (MAD): 15 days (including PTM dosing for all subjects on Day -1)

Part C (Adaptive SAD and/or MAD): 1 or 15 days (including PTM dosing for all multiple-dose subjects on Day -1)

3.5. Clinic Confinement

Following Screening and admission procedures, eligible subjects will be confined to the study center beginning at admission (Day -1 for SAD cohorts; Day -2 for MAD cohorts) until the completion of assessments on Day 4 (SAD cohorts), or Day 17 (MAD cohorts).

Subjects will return 14 ± 2 days after the last dose for an in-clinic follow-up visit (i.e., Day 15 [± 2 days] for SAD cohorts; Day 28 [± 2 days] for MAD cohorts.

3.6. Pharmacokinetic Assessments

PK assessments will occur on assigned study days and time points as outlined in Table 5 and Table 6, and Section 6.5.

Plasma concentrations of TLC-2716 (and its metabolites, as applicable) will be determined and PK evaluated. PK parameters will be estimated, as appropriate. Plasma concentrations of other metabolites may also be determined and PK explored.

3.7. Pharmacodynamic Assessments

Blood samples and stool samples for assessment of PD biomarkers related to TLC-2716 and its mechanism of action will be collected throughout the study as outlined in Table 5 and Table 6, and Section 6.6.

3.8. Biomarker Sample for Optional Genomic Research

In addition to the study-specific informed consent to be signed by each subject participating in the study, subjects will be required to document an agreement to provide additional samples for optional genomic research. From subjects who agree to participate and provide their additional specific consent, a separate blood specimen will be collected to isolate DNA for genomic testing. This specimen will be used to study genetic and genomic mechanisms that may contribute to underlying metabolic diseases and outcomes. Stored specimens may also be used to evaluate the association of genetic and genomic markers with study drug response, including metabolism and/or AEs, and to determine future treatment predictions for TLC-2716 and/or other approved or exploratory medications. This sample should be collected on Day -1 prior to dosing but may be collected at any time during the study, if necessary.

The specimen collected for optional genomic research will be destroyed no later than 15 years after the end of the study or per country requirements.

3.9. Safety Assessments

Safety assessments will be performed throughout the study as outlined in Table 5, Table 6, and Section 6.7.

3.10. End of Study

The end of this study will be the last subject's last observation (or visit).

4. SUBJECT POPULATION

4.1. Number of Subjects and Subject Selection

A total of up to 150 unique subjects will be enrolled in the study, with an approximately even distribution of healthy male and non-pregnant, non-lactating female subjects, 18 to 55 years of age, inclusive, at Screening.

4.1.1. Subject Replacement

If necessary, replacement subjects may be enrolled after discussion and approval from the TLC MM, or designee, if subjects do not complete all PK procedures or the subject is considered non-evaluable. Replacement subjects will not be enrolled for subjects who discontinue the study due to study drug-related AEs.

4.2. Inclusion Criteria

Subjects must meet all of the following inclusion criteria to be eligible for participation in this study.

1. Subject must have the ability to understand and sign a written informed consent form (ICF), which must be obtained prior to initiation of study procedures.

2. Male or female subject between 18 and 55 years of age, inclusive, at Screening.

3. Subject must be a nonsmoker. The use of nicotine or nicotine-containing products must be discontinued 90 days prior to the first dose of the study drug.

4. Subject must have a calculated body mass index (BMI) from 19 to 35 kg/m^2, inclusive, at study Screening.

5. Estimated glomerular filtration rate (eGFR) ≥ 80 mL/min, as calculated by the Modification of Diet in Renal Disease (MDRD) equation as outlined in Section 6.7.6.

6. Subject must have normal liver biochemistry tests: AST, ALT, ALP, and GGT within the normal range. Total bilirubin 1.0 to 1.5 x ULN is permitted in subjects with a medical history of Gilbert's syndrome.

7. Screening laboratory evaluations (hematology, chemistry, and urinalysis) must fall within the normal range of the local laboratory's reference ranges unless the results have been determined by the investigator to have no clinical significance. Note, for Parts B and C, attempts will be made to enroll at least 3 subjects in each cohort with Screening triglycerides ≥ 150 mg/dL and/or low-density lipoprotein cholesterol (LDL-C) ≥ 130 mg/dL.

8. Subject must have either a normal 12-lead electrocardiogram (ECG) or one with abnormalities that are considered clinically insignificant by the investigator.

9. Females of childbearing potential (as defined in Appendix 3) must have a negative pregnancy test at Screening and clinic admission.

10. Male subjects and female subjects of childbearing potential who engage in heterosexual intercourse must agree to use protocol-specified method(s) of contraception (as described in Appendix 3).

11. Male subjects must refrain from sperm donation from clinic admission, throughout the study period, and continuing for at least 90 days following the last dose of the study drug.

12. Female subjects must refrain from egg donation and *in vitro* fertilization from clinic admission, throughout the study period, and continuing for at least 90 days following the last dose of the study drug.

13. Subjects have not donated blood within 56 days of study entry or plasma within 7 days of study entry and must refrain from blood donation from clinic admission, throughout the study period, and continuing for at least 30 days following the last dose of the study drug.

14. Must, in the opinion of the investigator, be in good health based upon medical history and physical examination, including vital signs.

15. Must be willing and able to comply with all study requirements.

4.3. Exclusion Criteria

Subjects who meet *any* of the following exclusion criteria will not be enrolled in this study.

1. Pregnant or lactating subjects.

2. Subjects with triglycerides ≥ 500 mg/dL.

3. Subjects with LDL ≥ 190 mg/dL.

4. Subjects who have any serious or active medical or psychiatric illness (including depression) that, in the opinion of the investigator, would interfere with the subject's treatment, assessment, or compliance with the protocol. This would include renal, cardiac, hematological, hepatic, pulmonary (including chronic asthma), endocrine (including diabetes), central nervous, gastrointestinal (including an ulcer or inflammatory bowel disease), vascular, metabolic (thyroid disorders, adrenal disease), immunodeficiency disorders, active infection, or malignancy that are clinically significant or requiring treatment.

5. Subjects who have received any investigational compound within 30 days or 5 half-lives, whichever is longer, prior to study drug dosing.

6. Men who habitually drink greater than 21 units/week of alcohol or women who habitually drink greater than 14 units/week of alcohol (one unit is equivalent to 12 oz/360 mL of beer, a

4 oz/120 mL glass of wine, or 1 oz/30 mL of hard liquor); or current alcohol abuse that is judged by the investigator to potentially interfere with the subject's compliance or safety.

7. Positive test for drugs of abuse, including alcohol at Screening or on Admission (Day -1 for SAD cohorts in Parts A and C; Day -2 for MAD cohorts in Parts B and C); or current substance abuse that is judged by the investigator to potentially interfere with the subject's compliance or safety.

8. A positive test result for human immunodeficiency virus (HIV-1) antibody, hepatitis B (HBV) surface antigen, or hepatitis C (HCV) antibody.

9. Subjects who have poor venous access that limits phlebotomy.

10. Subjects who have taken any prescription medications or over-the-counter medications, including herbal products, within 28 days prior to start of study drug dosing, with the exception of vitamins, acetaminophen (paracetamol), ibuprofen, and/or hormonal contraceptive medications.

11. Subjects who have been treated with systemic steroids, immunosuppressant therapies, or chemotherapeutic agents within 3 months prior to Screening or expected to receive these agents during the study (e.g., corticosteroids, immunoglobulins, and other immune- or cytokine-based therapies).

12. Medical history of serious skin disease in the opinion of the investigator, such as but not limited to rash, food allergy, eczema, psoriasis, or urticaria.

13. Medical history of drug sensitivity or drug allergy (such as anaphylaxis or hepatoxicity).

14. Known hypersensitivity to the study drugs, their metabolites, or formulation excipients (see Section 5.2.1).

15. Presence or history of cardiovascular disease, including significant cardiovascular disease (including a history of myocardial infarction based on ECG and/or clinical history), history of cardiac conduction abnormalities (including any history of ventricular tachycardia), congestive heart failure, cardiomyopathy with left ventricular ejection fraction < 40%, a family history of Long QT Syndrome, or unexplained death in an otherwise healthy individual between the ages of 1 and 30 years.

16. Syncope, palpitations, or unexplained dizziness.

17. Implanted defibrillator or pacemaker.

18. Medical history of liver disease, including but not limited to alcoholic liver disease, autoimmune disorders (e.g., primary biliary cholangitis, primary sclerosing cholangitis, autoimmune hepatitis), drug-induced hepatotoxicity, Wilson disease, clinically significant iron overload, or alpha-1-antitrypsin deficiency).

19. Severe peptic ulcer disease, gastroesophageal reflux disease, or other gastric acid hypersecretory conditions.

20. History of medical or surgical treatment that permanently alters intestinal absorption (e.g., gastric or intestinal surgery). *History of cholecystectomy is not exclusionary.*

21. Unable to comply with study requirements or are otherwise believed, by the study investigator, to be inappropriate for study participation for any reason.

22. Subjects who have received vaccination for COVID-19 within 14 days of Admission (Day -1 in SAD cohorts, Day -2 in MAD cohorts).

5. STUDY DRUGS

5.1. Randomization, Blinding, and Treatment Codes Access

5.1.1. Randomization

It is the responsibility of the investigator to ensure that the subject is eligible for the study prior to enrollment and all enrollment criteria data are appropriately recorded. Subjects will be assigned a Screening number at the time of signing informed consent. Once a subject number has been assigned to a subject, it will not be reassigned to another subject. If necessary, replacement subjects may be enrolled (to compensate for early dropouts) after discussion and approval from the sponsor. A new unique subject number will be assigned to the replacement subject.

At Screening, study subjects will be assigned to Part A, B, or C, followed by assignment to a cohort within their respective part. Following confirmation of study eligibility on Day -1 (SAD cohorts) or Day -2 (MAD cohorts), eligible subjects will be randomized 4:1 to receive TLC-2716 or PTM and will be assigned a subject number. Note, for SAD cohorts in Part A and for any adaptive cohort in Part C evaluating a total daily dose that is higher than has previously been evaluated, two sentinel subjects, one randomized to TLC-2716 and one to PTM, will be evaluated through Day 2 prior to the randomization of the additional eight subjects in each cohort.

A randomization scheme including subject number and treatment assignments will be provided by the Sponsor to the unblinded study pharmacist for storing in a secure location. Blinded study site investigators and staff will not have access to this list. The study pharmacist, or designee, will remain unblinded throughout the study to allow for preparation of study drug.

5.1.2. Blinding

During the randomized phase, TLC study team and site pharmacist(s) will be unblinded, while the investigational sites, including investigators and the subjects participating in the study will remain blinded. To mitigate the risks of inadvertently releasing the treatment information to the site or subjects, TLC staff will only be provided with the unblinded information when there is a need to access such information for data analysis to support internal decision making. Should TLC staff receive unblinded information unnecessarily, they will keep it confidential and will not communicate the information to the blinded site staff or subjects.

5.1.3. Procedures for Breaking Treatment Codes

In the event of a medical emergency in which the treatment code must be revealed to provide medical care to the subject, the investigator may obtain treatment assignment for that subject. TLC recommends but does not require that the investigator contact the TLC MM before breaking the blind. Treatment assignment should remain blinded unless that knowledge is necessary to determine subject emergency medical care. The rationale for unblinding must be clearly explained in source documentation, along with the date on which the treatment assignment was

obtained. The investigator is requested to contact the TLC MM promptly in case of any treatment unblinding.

Blinding of study treatment is critical to the integrity of this clinical study and therefore, if a subject's treatment assignment is disclosed to the investigator, the subject will have study treatment discontinued. All subjects will be followed until study completion unless consent to do so is specifically withdrawn by the subject.

5.2. Description and Handling of Study Drugs

5.2.1. Formulation

The TLC-2716 capsules are available in strengths of 0.5 mg, 2 mg, and 5 mg (as free form equivalent). The capsules are opaque, hard hypromellose capsule shells, and are available in white (0.5 mg), rich yellow (2 mg), and Swedish orange (5 mg). In addition to the active ingredient, each capsule contains inactive ingredients, including lactose, microcrystalline cellulose, mannitol, and HPMC E5.

The PTM TLC-2716 capsules are identical in weight, shape, color, and appearance to the corresponding strengths of active TLC-2716 capsules.

5.2.2. Packaging and Labeling

TLC-2716 capsules are packed in white, high-density polyethylene bottles with silica gel desiccant and polyester packing material. Each bottle contains 30 capsules and is capped with a child-resistant polypropylene screw cap containing desiccant fitted with an induction-sealed, aluminum-faced liner. Study drugs to be distributed to study centers shall be labeled to meet all applicable requirements of the FDA and/or other local regulations, as applicable.

5.2.3. Storage and Handling

TLC-2716 capsules and PTM TLC-2716 capsules should be stored at refrigerated temperatures (2-8°C). Storage conditions are specified on the label. Until study drug capsules are dispensed to the subjects, all bottles of study drugs should be stored in a securely locked area, accessible only to authorized site personnel. To ensure stability and proper identification, the drug products should not be stored in a container other than the container in which they were supplied. Consideration should be given to handling, preparation, and disposal through measures that minimize drug contact with the body. Appropriate precautions should be followed to avoid direct eye contact or exposure through inhalation when handling TLC-2716 capsules and PTM TLC-2716 capsules.

5.3. Administration of Study Drug

In Parts A and B, all study treatments will be administered orally at the study center in the morning at approximately the same time each day, within 5 minutes of completing a standardized meal, and with water. The standardized meal should be initiated 30 minutes prior to study drug administration. Except for the water provided with the study drug(s) and water or

beverages with the standardized meal, subjects will be restricted from water consumption from 1 hour before through 2 hours after dosing.

In Part C, study drug may be administered in a fed or fasted state, QD or BID (BID only in MAD cohorts). In the case of QD dosing in fed state, study drug should be administered as in Parts A and B described above. In the case of fasted state or BID dosing, please see Section 5.3.1 for instructions.

5.3.1. Fasting and Meals

Fed State Dosing: In Parts A and B, after the collection of fasting safety labs, a standardized meal will be provided and all study treatments will be administered in the fed state. Study drug(s) will be administered at approximately the same time each day and within 5 minutes of completing a standardized meal. The meal should be initiated 30 minutes prior to study drug administration. Following study drug administration, subjects will fast until after collection of the 4-hour PK sample on PK assessment days and 2 hours after study drug administration on all other days. On days of PK assessments, other than the water and beverages provided with study drug(s) and with the standardized meal, water, and other fluids will be withheld for 1 hour before until 2 hours after study drug administration. Water may be consumed following the 2-hour post-dose blood draw for the remainder of the collection period.

In Part C, for potential BID dosing in fed state, breakfast will be initiated 30 minutes prior to study drug administration (first daily dose of BID dosing). The second daily dose of BID dosing will be administered 12 hours (± 10 minutes) after the morning dose, and approximately 30 minutes after starting an evening snack, which should occur at approximately the same time each day.

Fasted State: Prior to collection of fasting safety labs (e.g., fasting serum lipids), as outlined in Table 5 for SAD cohorts and Table 6 for MAD cohorts, subjects are required to conduct an overnight fast (no food or drinks except water) for at least 10 hours prior to sample collection.

Fasted State Dosing: In Part C, if applicable, study drug will be administered at approximately the same time each day with water following an overnight fast (no food or drinks except water) for at least 10 hours. Subjects will continue to fast until after the 4-hour PK sample on days of PK sampling and 2 hours after study drug administration on all other days.

Additionally, on PK sampling days, subjects will be restricted from water consumption 1 hour before until 2 hours after dosing, except for the water given with study treatment. A standardized meal will be provided to subjects after the 4-hour post-dose PK blood draw, or as early as 2 hours post-dose on days without post-dose PK sampling.

5.4. Dispensing, Accountability, and Disposal or Return of Study Drug

The investigator or designee (e.g., study pharmacist) will acknowledge receipt of the study drug (after reviewing the shipment's content and condition) from TLC (or designee). The investigator will maintain an accurate inventory of all study drug(s). Each dose of the study drug(s)

administered at the study center will be administered by qualified study center staff. The dose of study drug(s) administered to subjects in the clinic under the supervision of staff will be accurately recorded on the Study Drug Accountability eCRF (or on equivalent documentation maintained by the study center), which indicates the date and quantity of each dosage formulation dispensed to individual subjects.

TLC recommends that used and unused study drug supplies, including empty containers, be returned to the shipping facility from where they came or TLC for destruction following drug accountability and drug inventory reconciliation.

If returning drug supplies to the shipping facility from where they came or to TLC is not possible, the monitor will evaluate the site's Standard Operating Procedure (SOP) for study drug disposal/destruction in order to ensure that it complies with TLC's requirements. At the end of the study, following the final drug inventory reconciliation by the monitor, the study site will dispose of and/or destroy all unused study drug supplies, including empty containers, according to these procedures.

5.5. Concomitant Medications and Other Protocol Restrictions

5.5.1. Concomitant Medications

The following medications are prohibited while subjects are participating in the study:

- Any prescription medications and over-the-counter medications including herbal products and antacids with the exception of vitamins, ibuprofen, and/or hormonal contraceptive medications. Acetaminophen (paracetamol) is permitted up to a maximum dose of 2 g/day. The short-term use of topical hydrocortisone cream or ointment to treat minor skin irritation (e.g., due to ECG leads) will be allowed. If a subject requires the use of a disallowed medication, a request for such use must be reviewed by the TLC MM or designee, and if approved, subjects may continue to participate in the study.
- COVID-19 vaccination, from 14 days prior to Admission (Day -1 in SAD cohorts, Day -2 in MAD cohorts) through the end of study participation
- Any and all illegal or illicit drugs, including prescription drugs consumed outside the care of the prescribing physician.

5.5.2. Other Protocol Restrictions

- Subjects will be required to refrain from the consumption of food and beverages containing alcohol 72 hours prior to Day -1 (SAD cohorts) or Day -2 (MAD cohorts) and during the study through the follow-up visit.
- Subjects will be required to refrain from the use of nicotine or nicotine-containing products 90 days prior to Day -1 (SAD cohorts) or Day -2 (MAD cohorts), and during the study through the follow-up visit.

- Subjects will be required to refrain from the consumption of grapefruit juice, grapefruits, and Seville orange juice 72 hours prior to Day -1 (SAD cohorts) or Day -2 (MAD cohorts) and during the study through the follow-up visit.
- While confined at the study center, tea, coffee, chocolate, and other foods and beverages containing caffeine and other methylxanthines will be prohibited on each dosing day. At all other times, caffeine-containing beverages and foodstuffs may be served or withheld in accordance with normal study center practice. Caffeine-containing beverages and foodstuffs will not be restricted while subjects are outside of the clinic.
- Subjects will be encouraged to avoid strenuous or prolonged exercise, as well as saunas, steam baths, and sunbathing or other prolonged ultraviolet light exposure (e.g., tanning salon) from the Screening evaluation until completion of the follow-up visit, as these activities are known to affect certain clinical laboratory test parameters (e.g., creatine kinase [CK]) and may provide false indicators of potential treatment-related toxicity.

Upon every admission to the clinic, each subject will be questioned as to their compliance with the above protocol restrictions**.** If a subject is unable to comply with any of the restrictions described above, the subject's continued participation in the study will be reevaluated by the investigator in consultation with the sponsor.

6. STUDY ASSESSMENTS

The study procedures to be conducted for each subject enrolled in the study are detailed below.

Any deviation from protocol procedures should be noted in the subject's clinical chart and appropriate electronic case report form (eCRFs). In addition, the sponsor should be promptly notified of any protocol deviations.

The study center will not initiate dosing until:

- The IRB/IEC/other applicable regulatory agencies have reviewed and approved the study and the informed consent document
- All requested regulatory documents have been submitted to and approved by TLC
- A Master Services Agreement and/or Study Agreement is executed
- The study initiation meeting has been conducted by TLC (or designee). The initiation meeting will include but is not limited to a review of the protocol, the IB, study drugs, and investigator responsibilities

Documentation of the personally signed and dated informed consent of each subject, using the study-specific, IRB/IEC-approved ICF, is required before initiating the Screening process.

6.1. Subject Enrollment and Treatment Assignment

It is the responsibility of the investigator to ensure that subjects are eligible to participate in the study prior to enrollment and continue to remain eligible throughout the study.

Once informed consent has been obtained, all Screening and admission tests and procedures have been assessed, and study eligibility has been confirmed, subjects will be enrolled to receive PTM or study drug starting on Day 1. All subjects in MAD cohorts in Parts B and C will receive PTM on Day -1.

Subjects will receive the study treatments as described in Section 5.3.

Table 5. Schedule of Assessments: SAD Cohorts in Parts A and C

Study Procedure	Screening[a]	Day -1 (Admission)	Day 1	Day 2	Day 3	Day 4[b]	Follow-up[c]: Day 15 (± 2)	ET[d]
Written informed consent	X[r]							
Medical history	X							
Height	X							
Weight & BMI	X	X				X	X	
Vital signs[e]	X	X	X	X	X	X	X	X
Complete physical exam	X	X				X	X	X
Symptom-driven physical exam[f]			X	X	X			
HIV-1, HBV, and HCV serology	X							
Hematology[g]	X	X	X[k]	X	X	X	X	X
Serum chemistry[h]	X	X	X[k]	X	X	X	X	X
eGFR	X							
Coagulation[i]	X	X	X[k]	X	X	X	X	X
Lipids[j]	X	X	X[k]	X	X	X	X	X
Urinalysis	X	X	X[k]	X	X	X	X	X
Serum pregnancy test[l]	X					X	X	X
Urine pregnancy test[l]		X						
FSH[m]	X							
Urine and alcohol drug screen	X	X						
12-Lead ECG	X	X	X[k]	X		X	X	X
Randomization	X	X						
Study drug or PTM administration			X					

Study Procedure	Screening[a]	Day -1 (Admission)	Day 1	Day 2	Day 3	Day 4[b]	Follow-up[c]: Day 15 (± 2)	ET[d]
Plasma PK[n]			X	X	X	X		
Plasma PD[o]			X					
Biomarkers: Exploratory[o]			X					
Optional genomic testing[p]		X						
Clinic confinement[q]		X	X	X	X	X		
Review AEs and concomitant medications[r]	X	X	X	X	X	X	X	X

AE = adverse event; BMI = body mass index; ECG = electrocardiogram; eGFR = estimated glomerular filtration rate; ET = early termination; FSH = follicle-stimulating hormone; HBV = hepatitis B virus; HCV = hepatitis C virus, HIV-1 = human immunodeficiency virus; PD = pharmacodynamic(s); PK = pharmacokinetic(s); PTM = placebo-to-match

a Prospective subjects should be screened no more than 28 days prior to administration of the first dose of study drugs.
b Subjects will be discharged from the center on Day 4, following all morning assessments.
c 14 (± 2) days after the last dose of the study drug, all subjects will return for an in-clinic follow-up visit.
d Assessments will be performed within 72 hours of early termination from the study.
e Vital signs include blood pressure, pulse, respiration rate, and body temperature.
f Symptom driven physical exams will be performed during confinement as needed, based on reported signs and symptoms, on days in which complete physical examination is not required.
g Hematology: CBC with differential. Collected in fasting state prior to morning meal.
h Serum Chemistry: alkaline phosphatase, AST, ALT, GGT, total bilirubin, direct and indirect bilirubin, total protein, albumin, bicarbonate, BUN, calcium, chloride, creatinine, glucose, phosphorous, magnesium, potassium, sodium, uric acid, and amylase (reflex lipase testing is performed in subjects with total amylase > 1.5 X ULN). Collected in fasting state prior to morning meal.
i Coagulation: INR, PTT. Collected in fasting state prior to morning meal.
j Lipids: total cholesterol, HDL, LDL, TG. Collected in fasting state prior to morning meal.
k To be collected in fasting state prior to morning meal and 12 hours post-dose, with exception of 12-lead ECG which is only required 2 hours post-dose.
l Females of child-bearing potential only (see Appendix 3 for definition).
m As necessary only for evaluation of post-menopausal state in females of any age with amenorrhea > 12 months at time of Screening (see Appendix 3).
n Collected pre-dose (≤ 10 minutes prior to dosing), 0.25, 0.5, 1, 1.5, 2, 2.5, 3, 3.5, 4, 6, 12, 24, 48, and 72 hours post-dose.
o Collected pre-dose (≤ 10 minutes prior to dosing), and 4 hours post-dose.
p The optional genomic sample should be collected on Day -1, but may be collected at any time during the study, if necessary.
q Subjects to be educated on study requirements and restrictions.
r From the time of obtaining informed consent through the first administration of the study drug, record all SAEs and any non-serious AEs related to protocol required procedures on the AE eCRF. All other untoward medical occurrences observed during the Screening period, including exacerbation or changes in medical history should be documented on the medical history eCRF. See Section 7 Adverse Events and Toxicity Management for additional details.

Table 6. Schedule of Assessments: MAD Cohorts in Parts B and C

Study Procedure	Screening[a]	Day -2 (Admission)	Day -1	Day 1	Day 2	Day 3	Day 4	Day 5	Day 6	Day 7	Days 8-9	Day 10	Days 11-13	Day 14	Days 15-16	Day 17[b]	Follow-up[c]: Day 28 (± 2)	ET[d]
Written informed consent	X[v]																	
Medical history	X																	
Height	X																	
Weight & BMI	X	X								X						X	X	
Vital signs[e]	X	X	X	X	X	X		X		X		X		X		X	X	X
Complete physical exam	X	X														X	X	X
Symptom-driven physical exam[f]			X	X	X	X	X	X	X	X	X	X	X	X	X			
HIV-1, HBV, & HCV serology	X																	
Hematology[g]	X		X	X[k]	X	X		X		X		X		X		X	X	X
Serum chemistry[h]	X		X	X[k]	X	X		X		X		X		X		X	X	X
eGFR	X																	
Coagulation[i]	X		X	X[k]	X	X		X		X		X		X		X	X	X
Lipids[j]	X		X	X[k]	X	X		X		X		X		X		X	X	X
Urinalysis	X		X	X[k]	X	X		X		X		X		X		X	X	X
Serum pregnancy test[l]	X															X	X	X
Urine pregnancy test[l]		X																
FSH[m]	X																	
Urine & alcohol drug screen	X	X																

**Study Procedure**	**Screening[a]**	**Day -2 (Admission)**	**Day -1**	**Day 1**	**Day 2**	**Day 3**	**Day 4**	**Day 5**	**Day 6**	**Day 7**	**Days 8-9**	**Day 10**	**Days 11-13**	**Day 14**	**Days 15-16**	**Day 17[b]**	**Follow-up[c]: Day 28 (± 2)**	**ET[d]**
12-Lead ECG	X	X		X[k]		X				X				X		X	X	X
Randomization			X															
Study drug or PTM administration[n]			X[n]	X[n]	X	X	X	X	X	X	X	X	X	X				
Plasma PK[o]				X		X				X				X	X	X		
Plasma PD[p]			X	X						X				X				
Biomarker: whole blood collection[q]				X										X				
Plasma NMR LipoProfile[r]				X										X				
Biomarker: Stool collection[s]			X											X				
Biomarkers: Exploratory[p]			X	X						X				X				
Optional genomic testing[t]			X															
Clinic confinement[u]		X	X	X	X	X	X	X	X	X	X	X	X	X	X	X		
Review AEs & concomitant medications[v]	X	X	X	X	X	X	X	X	X	X	X	X	X	X	X	X	X	X

AE = adverse event; BMI = body mass index; ECG = electrocardiogram; eGFR = estimated glomerular filtration rate; ET = early termination; FSH = follicle-stimulating hormone; HBV = hepatitis B virus; HCV = hepatitis C virus; HIV-1 = human immunodeficiency virus; PD = pharmacodynamic(s); PK = pharmacokinetic(s); PTM = placebo-to-match

a Prospective subjects should be screened no more than 28 days prior to administration of the first dose of study drugs.
b Subjects will be discharged from the center on Day 17, following all morning assessments.
c 14 (± 2) days after the last dose of the study drug, all subjects will return for an in-clinic follow-up visit.
d Assessments will be performed within 72 hours of early termination from the study.
e Vital signs include blood pressure, pulse, respiration rate, and body temperature.
f Symptom driven physical exams will be performed during confinement as needed, based on reported signs and symptoms.
g Hematology: CBC with differential. Collected in fasting state prior to morning meal, if applicable.

h Serum Chemistry: alkaline phosphatase, AST, ALT, GGT, total bilirubin, direct and indirect bilirubin, total protein, albumin, bicarbonate, BUN, calcium, chloride, creatinine, glucose, phosphorous, magnesium, potassium, sodium, uric acid, and amylase (reflex lipase testing is performed in subjects with total amylase > 1.5 X ULN). Collected in fasting state prior to morning meal, if applicable.
i Coagulation: INR, PTT. Collected in fasting state prior to morning meal, if applicable.
j Lipids: total cholesterol, HDL, LDL, TG. Collected in fasting state prior to morning meal, if applicable.
k On Day 1, to be collected 12 hours post-dose only, with exception of 12-lead ECG which should be 2 hours post-dose.
l Females of child-bearing potential only (see Appendix 3 for definition).
m As necessary only for evaluation of post-menopausal state in females of any age with amenorrhea > 12 months at time of Screening (see Appendix 3).
n For MAD cohorts, all subjects will receive PTM on Day -1, and study drug starting Day 1.
o PK sampling will occur relative to the morning dosing of TLC-2716 at the following time points for each cohort:
- Day 1: Pre-dose (≤ 10 minutes prior to dosing), 0.25, 0.5, 1, 1.5, 2, 2.5, 3, 3.5, 4, 6, and 12 hours post-dose
- Days 3, and 7: Pre-dose (≤ 10 minutes prior to dosing), 2, and 4 hours post-dose
- Day 14: Pre-dose (≤ 10 minutes prior to dosing), 0.25, 0.5, 1, 1.5, 2, 2.5, 3, 3.5, 4, 6, 12, 24, 48, and 72 hours post-dose

For any cohorts in Part C with BID administration, see Protocol Section 6.5 for details on the timing of PK collections.
p Collected relative to the morning dosing of TLC-2716 or PTM to measure PD biomarkers for TLC-2716 at the following time points:
- Day -1: (all ± 5 minutes of Day 1 time points), 0, 2, 4, 6, and 12 hours
- Day 1, 7, and 14: Pre-dose (≤ 10 minutes prior to dosing), and 2, 4, 6, and 12 hours post-dose

All post-dose timings are relative to morning dose for once-daily dosing or BID dosing.
q Collected pre-dose (≤ 10 minutes prior to dosing), and 4 hours post-dose.
r Collected pre-dose (≤ 10 minutes prior to dosing), 2, 4, 6, and 12 hours post-dose.
s All stools will be collected over approximately 24-hour intervals at the following time points:
- Day -1: Day -2 Admission through Day 1 (0, pre-dose)
- Days 14: 0 (pre-dose) through to 24 hours post-dose

t The optional genomic sample should be collected on Day -1, but may be collected at any time during the study, if necessary.
u Subjects to be educated on study requirements and restrictions.
v From the time of obtaining informed consent through the first administration of the study drug, record all SAEs and any non-serious AEs related to protocol required procedures on the AE eCRF. All other untoward medical occurrences observed during the Screening period, including exacerbation or changes in medical history should be documented on the medical history eCRF. See Section 7 Adverse Events and Toxicity Management for additional details.

6.2. Pretreatment Assessments

6.2.1. Screening Visit

Prospective subjects should be screened no more than 28 days prior to administration of the first dose of the study drug. If the subject does not begin the treatment phase within this 28-day window, all Screening evaluation procedures must be repeated. Screening labs may be repeated once within 28 days prior to administration of the study drug to rule out laboratory error, in the opinion of the investigator.

A sufficient number of subjects will be screened to identify up to 100 subjects for enrollment in Parts A and B and, as necessary, an additional 50 subjects in Part C.

Subjects should be instructed to fast (no food or drink except water, for at least 10 hours), starting from 10 PM (22:00) or earlier, as appropriate, on the evening prior to the Screening visit to ensure an approximate 10-hour fast prior to the fasted blood sample collection the next morning.

Written informed consent must be obtained from each subject before the initiation of any Screening procedure. After a subject has provided informed consent, the investigator and other study personnel will determine if the subject is eligible for participation in the study. This assessment will include a review of the inclusion/exclusion criteria and completion of all Screening procedures as outlined in Table 5 (for SAD cohorts) and Table 6 (for MAD cohorts) and described in the following text.

Eligible subjects meeting all inclusion criteria and none of the exclusion criteria will be instructed on all protocol requirements, including the restrictions on concomitant medication usage and other substances as well as consumption of food or beverages containing alcohol, caffeine, or xanthine. Subjects will be asked to arrive at the study center on Day -1 (for SAD cohorts) and Day -2 (for MAD cohorts) for admission assessments.

From the time of obtaining informed consent through the first administration of the study drug, record all SAEs, as well as any AE related to protocol-mandated procedures on the AE eCRF. All other untoward medical occurrences observed during the Screening period, including exacerbation or changes in medical history, are to be captured on the medical history eCRF. See Section 7 Adverse Events and Toxicity Management for additional details.

6.2.2. Admission Assessments

6.2.2.1. Admission

Subjects meeting all eligibility criteria following the Screening evaluation will return to the clinic for admission assessments on Day -1 (for SAD cohorts) and Day -2 (for MAD cohorts). The admission evaluations and/or procedures are outlined in Table 5 and Table 6.

Prior to randomization on Day -1, the results of Screening clinical and laboratory evaluations (as described in Table 5 and Table 6) must be reviewed by the investigator to confirm the continued

eligibility of each subject to participate in the study. At the time of randomization, subjects will be assigned a sequential subject number as described in Section 5.1. Subjects will remain confined to the study clinic for the duration as described in Section 6.2.2.2 and Table 5 or Table 6, as appropriate.

6.2.2.2. Clinic Confinement

Following Screening and admission procedures, eligible subjects will be confined to the study center beginning at admission (Day -1 for SAD cohorts; Day -2 for MAD cohorts) until the completion of assessments on Day 4 (SAD cohorts) or Day 17 (MAD cohorts).

6.3. Check-In Assessments

Following confirmation of study eligibility at Admission, eligible subjects will be randomized to receive TLC-2716 or PTM starting on Day 1 and assigned a subject number and study drug administration time. Note, for MAD cohorts, regardless of randomization outcome, all subjects will receive PTM corresponding to the dose to be evaluated in the planned cohort on Day -1.

Study procedures and assessments are outlined in Table 5 (for SAD cohorts) and Table 6 (for MAD cohorts).

6.4. Treatment Assessments

Study procedures and assessments are outlined in Table 5 (for SAD cohorts) and Table 6 (for MAD cohorts).

6.5. Pharmacokinetic Assessments

PK assessments following single- or multiple-dose study drug administration, including but not limited to the following plasma PK parameters of TLC-2716 and its metabolites will be calculated as appropriate:

AUC_{last}, AUC_{inf} (single-dose), AUC_{tau} (multiple-dose), $\%AUC_{exp}$ (single-dose), C_{max}, T_{max}, C_{last}, T_{last}, C_{tau} (multiple-dose), λ_z, CL/F, and $t_{1/2.}$

6.5.1. Plasma PK Collection

SAD Cohorts in Parts A and C:

PK sampling will occur relative to the morning dosing of TLC-2716 or PTM at the following time points for each cohort:

Day 1: Pre-dose (≤ 10 minutes prior to dosing), 0.25, 0.5, 1, 1.5, 2, 2.5, 3, 3.5, 4, 6, 12, 24, 48, and 72 hours post-dose

MAD Cohorts in Parts B and C:

Day 1: Pre-dose (≤ 10 minutes prior to dosing), 0.25, 0.5, 1, 1.5, 2, 2.5, 3, 3.5, 4, 6 and 12 hours post-dose

Days 3, and 7: Pre-dose (≤ 10 minutes prior to dosing), 2, and 4 hours post-dose

Day 14: Pre-dose (≤ 10 minutes prior to dosing), 0.25, 0.5, 1, 1.5, 2, 2.5, 3, 3.5, 4, 6, 12, 24, 48, and 72 hours post-dose

If in any MAD cohort in Part C, BID administration is evaluated, PK assessments should be performed at the following time points.

Day 1: Pre-dose morning (≤ 10 minutes prior to dosing), 0.25, 0.5, 1, 1.5, 2, 2.5, 3, 3.5, 4, 6, and 12 hours post morning dose (12-hour time point collected prior to evening dose)

Days 3 and 7: Pre-dose morning (≤ 10 minutes prior to dosing), 2, and 4 hours post morning dose

Day 14: Pre-dose morning (≤ 10 minutes prior to dosing), 0.25, 0.5, 1, 1.5, 2, 2.5, 3, 3.5, 4, 6, and 12 hours post morning dose (12-hour time point collected prior to evening dose), 12.5, 13, 14, 16, 18, 24, 48, and 72 hours post-dose

Every effort should be made to collect PK samples at the scheduled time point.

Plasma concentrations of TLC-2716 (and its metabolites, as applicable) will be determined and other PK parameters evaluated, as appropriate. Sampling time points may be modified by the sponsor based on emerging data and assay feasibility.

For all cohorts, a single PK sample will be collected at the ET visit, if applicable.

6.6. Pharmacodynamic Assessments

Relevant PD biomarkers including measures of DNL, ANGPTL3, a comprehensive serum lipid profile (e.g., LDL-C, HDL-C, TG, ApoB), and other exploratory markers, will be determined and evaluated.

Serum lipids will be evaluated on safety lab collections in the fasting state prior to the standard morning meal and study drug dosing, with the exception of 12 hours post-dose on Day 1 following the first administration of study drug or PTM, as outlined in Table 5 (for SAD cohorts) and Table 6 (for MAD cohorts).

Dose and/or exposure-response relationships for LXR inverse agonism by TLC-2716 will be evaluated.

6.6.1. Plasma and/or Serum Pharmacodynamic Assessments

Blood samples will be collected to measure PD biomarkers for TLC-2716 at the time points described below, where ‘0’ indicates the pre-dose time point in the morning. Blood samples for PD assessments will be collected in the same subject order on each day, including the pre-dose

day, in each cohort. For MAD cohorts, the collection times on the day prior to the dosing day (e.g., Day -1) must match those on the days of subsequent dosing (± 5 minutes).

SAD Cohorts in Parts A and C:

Day 1: Pre-dose (≤ 10 minutes prior to dosing) and 4 hours post-dose

MAD Cohorts in Parts B and C:

Day -1: (all ± 5 minutes of Days 1, 7, and 14 time points), 0, 2, 4, 6, and 12 hours

Days 1, 7, and 14: Pre-dose (≤ 10 minutes prior to dosing), and 2, 4, 6, and 12 hours post-dose

All post-dose timings above are relative to the morning dose for QD or BID dosing. In the case of BID dosing in Part C, the 12 hours post-dose time point must be collected prior to the second daily dose of the study drug.

Sampling time points may be modified by the sponsor based on emerging data and assay feasibility.

6.6.2. Stool Pharmacodynamic Assessments

All stools, if available, will be collected once over approximately 24-hour intervals to measure PD and other exploratory biomarkers for TLC-2716 at the time points described below.

SAD Cohorts in Parts A and C: None

MAD Cohorts in Parts B and C: Single sample collections

Day -1: One specimen will be collected between Day -2 Admission and Day 1 (0, pre-dose)

Day 14: One specimen will be collected between time point 0 (pre-dose) and 24 hours post-dose

6.6.3. Biomarker Assessments

Additional Biomarker Assessments

To support evaluation of the effects of TLC-2716 on cholesterol and lipid homeostasis, a separate serum aliquot will be collected for potential exploratory biomarker assessments, including but not limited to IDOL, apolipoprotein C and E, and NMR-based lipid particle profiles.

Whole blood samples will also be collected to measure ABCA1 and ABCG1 mRNA expression, at the time points described below, where ‘0’ corresponds to the immediately pre-dose time point in the morning on Day 1 or later as applicable. Blood samples for biomarkers will be collected in the same subject order on each day, including the pre-dose day, in each cohort. The collection times on Day -1 must match the time of the collection on the day of dosing time points (± 5 minutes).

NMR LipoProfile

SAD Cohorts in Parts A and C: None

MAD Cohorts in Parts B and C:

Days 1 and 14: Pre-dose (≤ 10 minutes prior to dosing), and 2, 4, 6, and 12 hours post-dose

All post-dose timings above are relative to the morning dose for QD dosing or BID dosing. In the case of BID dosing in Part C, the 12 hours post-dose time point must be collected prior to the second daily dose of the study drug.

Exploratory Biomarkers:

SAD Cohorts in Parts A and C:
Day 1: Pre-dose (≤ 10 minutes prior to dosing) and 4 hours post-dose

MAD Cohorts in Parts B and C:
Day -1: (all ± 5 minutes of Day 1 time points), 0, 2, 4, 6, and 12 hours
Days 1, 7, and 14: Pre-dose (≤ 10 minutes prior to dosing), 2, 4, 6, and 12 hours post-dose

All post-dose timings above are relative to the morning dose for QD dosing or BID dosing. In the case of BID dosing in Part C, the 12 hours post-dose time point must be collected prior to the second daily dose of the study drug.

Whole Blood Collection:

SAD Cohorts in Parts A and C: None

MAD Cohorts in Parts B and C:

Days 1 and 14: Pre-dose (≤ 10 minutes prior to dosing), 4 hours post-dose

Sampling time points may be modified by the sponsor based on emerging data and assay feasibility.

6.7. Safety Assessments

Safety will be evaluated by assessment of clinical laboratory tests, ECGs, complete and symptom-driven physical examinations including vital signs, at various time points throughout the study, and by the documentation of AEs and concomitant medications throughout the study.

Refer to Table 5 (for SAD cohorts) and Table 6 (for MAD cohorts) for a schedule of assessments.

If subjects experience any clinically significant AEs during the in-clinic period, they may remain in-clinic for further observation at the discretion of the investigator. Additional follow-up visits may be undertaken, as needed, for the assessment of persistent AEs and laboratory abnormalities.

Evaluations with abnormal results believed to be possibly or probably related to study treatment should be repeated weekly or as often as deemed appropriate by the investigator until the abnormality resolves, returns to baseline visit levels, or is otherwise explained.

6.7.1. Body Mass Index

Height will be collected at Screening for the calculation of BMI for inclusion criteria.

Weight will be collected and BMI will be calculated at the following time points:

- SAD cohorts: Screening, Day -1, the day of discharge, and at the in-clinic follow-up visit.
- MAD cohorts: Screening, Day -2, Day 7, the day of discharge, and at the in-clinic follow-up visit.

6.7.2. Physical Examination

Complete physical exam: Screening, Day -1 (for SAD cohorts) and Day -2 (for MAD cohorts), the day of discharge, and at the in-clinic follow-up visit or at the ET visit (if applicable).

The complete physical examination conducted at Screening will also include a review of medical history, including any history of allergies, and prior (30 days prior to Screening) and current use of nicotine or nicotine-containing products, alcohol, illegal drugs use, and medications.

Symptom-driven physical exam: As needed on days of confinement based on reported signs and symptoms.

6.7.3. Vital Signs

Vital sign measurements include blood pressure, pulse, respiration rate, and body temperature and should be taken once subjects have been seated or in the supine position. The subject position for measurement should be kept consistent throughout the study.

Refer to Table 5 (for SAD cohorts) and Table 6 (for MAD cohorts) for time points for collection of vital signs:

- SAD cohorts: Screening, Days -1, 1, 2, 3, the day of discharge, and at the in-clinic follow-up visit or at the ET visit (if applicable)
- MAD cohorts: Screening, Days -2, -1, 1, 2, 3, 5, 7, 10, 14, the day of discharge, and at the in-clinic follow-up visit or at the ET visit (if applicable)

6.7.4. Electrocardiogram Assessment

Subjects should rest quietly in the supine position for a minimum of 10 minutes prior to each scheduled ECG acquisition and should remain in that position until the recording is complete. There should be no environmental distractions (e.g., TV, radio, conversation, etc.) while the subjects are resting prior to and during the recordings. ECGs will be recorded using the site's standard ECG equipment. All ECGs will be obtained using instruments that analyze data using

the same algorithms and produce the same data for interpretation. Electrode placement will be performed according to the method of Wilson, Goldberger, and Einthoven with a check to confirm that the aVR lead is not inverted.

The investigator or other qualified individuals at the study center will review ECGs to assess for changes in ECG intervals and morphology as compared to pretreatment ECGs. Outputs of ECG interval measurements by the machine will be used for bedside safety monitoring.

Collection of additional ECGs for routine safety monitoring at additional time points or days is at the discretion of the investigator based on GCP.

Time points for 12-lead ECG:

- SAD cohorts: Screening, Days -1, 1 (2 hours post-dose), 2, the day of discharge, and at the in-clinic follow-up visit or at the ET visit (if applicable)
- MAD cohorts: Screening, Days -2, 1 (2 hours post-dose), 3, 7, 14, the day of discharge, and at the in-clinic follow-up visit or at the ET visit (if applicable)

6.7.5. Clinical Laboratory Tests/Assessments

Blood and urine samples for safety evaluations will be collected throughout the study as outlined in Table 5 (for SAD cohorts) and Table 6 (for MAD cohorts).

6.7.5.1. Blood Sampling

Blood samples will be collected for the following laboratory analyses:

- HIV, HBV, and HCV testing (Screening only)
- Clinical laboratory tests (hematology, serum chemistry, coagulation, lipids, and urinalysis):

  SAD cohorts: Screening, Days -1, 1 (prior to morning meal and 12 hours post-dose), 2, 3, the day of discharge, and at the in-clinic follow-up visit or at the ET visit (if applicable)

  MAD cohorts: Screening, Days -1, 1 (prior to morning meal and 12 hours post-dose), 2, 3, 5, 7, 10, 14, the day of discharge, and at the in-clinic follow-up visit or at the ET visit (if applicable)
- Hematology: CBC with differential
- Serum chemistry (fasting): alkaline phosphatase, AST, ALT, GGT, total bilirubin, direct and indirect bilirubin, total protein, albumin, bicarbonate, BUN, calcium, chloride, creatinine, glucose, phosphorus, magnesium, potassium, sodium, uric acid, and amylase (reflex lipase testing will be performed in subjects with total amylase > 1.5 × ULN).
- Coagulation: INR, PTT
- Lipids (fasting): total cholesterol, HDL-C, LDL-C, TG
- Urinalysis

- Serum pregnancy test (females of childbearing potential only): Screening, the day of discharge, and at the in-clinic follow-up visit or at the ET visit (if applicable).

6.7.5.2. Urine Samples

Urine samples will be collected for urinalysis, as described above, and urine alcohol and drug Screening assessments, the latter performed at Screening and Day -1 (SAD cohorts) or Day -2 (MAD cohorts). A urine pregnancy test will be performed on Day -1 (SAD cohorts) or Day -2 (MAD cohorts).

6.7.6. Estimated Glomerular Filtration Rate Using MDRD

Estimated glomerular filtration rate will be calculated by the laboratory. If the central laboratory is unable to perform the eGFR calculation, eGFR may also be calculated by the site.

Estimated glomerular filtration rate is calculated by the MDRD equation (conventional units):

- eGFR (mL/min/1.73 m²) = $175 \times (Scr)^{-1.154} \times (Age)^{-0.203} \times (0.742 \text{ if female}) \times (1.212 \text{ if Black})$

Scr = serum creatinine (mg/dL)

6.7.7. Adverse Events/Concomitant Medications/Protocol Restrictions

Evaluation for AEs, review of concomitant medications, and review of protocol restrictions will occur at the times shown in Table 5 and Table 6. See Section 7 for additional information regarding AEs and Section 4.3 and Section 5.5.1 for more information regarding concomitant medications.

6.8. Posttreatment Assessments – Follow-up Visit

Subjects will return 14 ± 2 days after the last dose for an in-clinic follow-up visit (Day 15 [± 2 days] for SAD cohorts; Day 28 [± 2 days] for MAD cohorts).

The assessments to be completed at the Follow-Up visit are outlined in Table 5 (for SAD cohorts) and Table 6 (for MAD cohorts).

6.9. Management of Subjects Following Premature Discontinuation of Study Drug or Study

If a subject discontinues study treatment dosing (see Section 3.3.1), for example, as a result of an AE meeting study drug discontinuation criteria, every attempt should be made to retain the subject in the study and perform the required study-related assessments, including in the absence of study drug dosing. If this is not possible or acceptable to the subject or investigator, the subject may be withdrawn from the study. Evaluations with abnormal results believed to be possibly or probably related to study treatment at the ET visit should be repeated weekly or as often as deemed appropriate by the investigator until the abnormality resolves, returns to baseline visit levels, or is otherwise explained.

If the subject discontinues prematurely from the study, the ET evaluations and/or procedures outlined in Table 5 (for SAD cohorts) and Table 6 (for MAD cohorts) should be performed within 72 hours of permanently discontinuing the study drug.

6.10. Optional Future Research

In addition to the study-specific informed consent to be signed by each subject participating in the main study, a separate, additional signature will be required to document a subject's agreement to allow the use of the remainder of their PK, PD, and biomarker specimens, collected as a component of the main study, for optional future research.

The specimens for optional future research will be used to increase our knowledge and understanding of the safety and efficacy of TLC-2716, including the evaluation of biomarkers with treatment outcomes, including efficacy, AEs, and the processes of drug absorption and disposition. These specimens may also be used to develop biomarker or diagnostic assays and establish the performance characteristics of these assays. The collection and analysis of optional future research specimens may also facilitate the rational design of new pharmaceutical agents and the development of diagnostic tests, which may allow for individualized drug therapy for patients in the future. Specimens may be stored by TLC for a period of 15 years after the end of study.

7. ADVERSE EVENTS AND TOXICITY MANAGEMENT

7.1. Definitions of Adverse Events, Adverse Reactions, and Serious Adverse Events

7.1.1. Adverse Events

An **AE** is any untoward medical occurrence in a clinical study subject administered a medicinal product, which does not necessarily have a causal relationship with the treatment. An AE can therefore be any unfavorable and/or unintended sign, symptom, or disease temporarily associated with the use of a medicinal product, whether or not considered related to the medicinal product. AEs may also include pre- or post-treatment complications that occur as a result of protocol-specified procedures, overdose, drug abuse/misuse reports, or occupational exposure. Preexisting events that increase in severity or change in nature during or as a consequence of participation in the clinical study will also be considered AEs.

An AE does not include the following:

- Medical or surgical procedures such as surgery, endoscopy, tooth extraction, and transfusion. The condition that led to the procedure may be an AE and must be reported.
- Preexisting diseases, conditions, or laboratory abnormalities present or detected before the Screening visit that do not worsen.
- Situations where an untoward medical occurrence has not occurred (e.g., hospitalization for elective surgery, social and/or convenience admissions).
- Overdose without clinical sequelae.
- Any medical condition or clinically significant laboratory abnormality with an onset date before the consent form is signed and not related to a protocol-associated procedure is not an AE, but rather, is considered to be preexisting and should be documented on the medical history CRF.

7.1.2. Clinical Laboratory Abnormalities and Other Abnormal Assessments as Adverse Events or Serious Adverse Events

Laboratory abnormalities without clinical significance are not recorded as AEs or SAEs. However, laboratory abnormalities (e.g., clinical chemistry, hematology, and urinalysis) that require medical or surgical intervention or lead to study drug interruption, modification, or discontinuation must be recorded as an AE, as well as an SAE, if applicable. In addition, laboratory or other abnormal assessments (e.g., ECG, X-rays, vital signs) that are associated with signs and/or symptoms must be recorded as an AE or SAE if they meet the definition of an AE or SAE as described in Sections 7.1.1 and 7.1.2. If the laboratory abnormality is part of a syndrome, record the syndrome or diagnosis (e.g., anemia), not the laboratory result (i.e., decreased hemoglobin).

7.1.3. Serious Adverse Events

An **SAE** is defined as an event that, at any dose, results in the following:

- Death
- Life-threatening (Note: The term "life-threatening" in the definition of "serious" refers to an event in which the subject was at risk of death at the time of the event; it does not refer to an event that hypothetically might have caused death if it were more severe)
- In-patient hospitalization or prolongation of existing hospitalization
- Persistent or significant disability or incapacity
- A congenital anomaly/birth defect
- A medically important event or reaction: such events may not be immediately life-threatening or result in death or hospitalization but may jeopardize the subject or may require intervention to prevent one of the other outcomes constituting SAEs. Medical and scientific judgment must be exercised to determine whether such an event is reportable under expedited reporting rules. Examples of medically important events include intensive treatment in an emergency room or at home (e.g., for allergic bronchospasm, blood dyscrasias, or convulsions that do not result in hospitalization), and the development of drug dependency or drug abuse. For the avoidance of doubt, infections resulting from a contaminated medicinal product will be considered a medically important event and subject to expedited reporting requirements.

7.2. Assessment of Adverse Events and Serious Adverse Events

The investigator or qualified sub-investigator is responsible for assessing AEs and SAEs for causality and severity, and for final review and confirmation of the accuracy of event information and assessments.

7.2.1. Assessment of Causality for Study Drugs and Procedures

The investigator or qualified sub-investigator is responsible for assessing the relationship to the study drug using clinical judgment and the following considerations:

No: Evidence exists that the AE has an etiology other than the study drug. For SAEs, an alternative causality must be provided (e.g., preexisting condition, underlying disease, intercurrent illness, or concomitant medication).

Yes: There is a reasonable possibility that the event may have been caused by the study drug.

It should be emphasized that ineffective treatment should not be considered as causally related in the context of AE reporting.

The relationship to study procedures (e.g., invasive procedures such as venipuncture or biopsy) should be assessed using the following considerations:

No: Evidence exists that the AE has an etiology other than the study procedure.
Yes: The AE occurred as a result of protocol-mandated procedures (e.g., venipuncture).

7.2.2. Assessment of Severity

AE severity should be recorded and graded using the Common Terminology Criteria for Adverse Events version 5.0 (Appendix 4). For AEs associated with laboratory abnormalities, the event should be graded on the basis of the clinical severity in the context of the underlying conditions; this may or may not be in agreement with the grading of the laboratory abnormality.

The distinction between the seriousness and the severity of an adverse event should be noted.

Severe is a measure of intensity; thus, a severe reaction is not necessarily a serious reaction. For example, a headache may be severe in intensity, but would not be classified as serious unless it met one of the criteria for serious events.

7.3. Investigator Requirements and Instructions for Reporting Adverse Events and Serious Adverse Events

7.3.1. Requirements for Collection Prior to Study Drug Initiation

After informed consent, but prior to initiation of study medication, the following types of events should be reported on the case report form (CRF/eCRF): all SAEs and AE related to protocol-mandated procedures.

7.3.1.1. Adverse Events

Following initiation of study medication, collect all AEs, regardless of cause or relationship, until 14 days after last administration of study drug and report to the CRF/eCRF database as instructed.

All AEs should be followed up until resolution or until the AE is stable, if possible.

TLC may request that certain AEs be followed beyond the protocol-defined follow-up period.

7.3.1.2. Serious Adverse Events

All SAEs, regardless of cause or relationship, that occur after the subject first consents to participate in the study (i.e., signing the informed consent) and throughout the duration of the study, including the protocol-required post-treatment follow-up period, must be reported to the CRF/eCRF database and TLC as instructed. This also includes any SAEs resulting from protocol-associated procedures performed after informed consent is signed.

Any SAEs and deaths that occur after the post-treatment follow-up visit but within 30 days of the last dose of study drug, regardless of causality, should also be reported.

Investigators are not obligated to actively seek SAEs after the protocol-defined follow-up period, however, if the investigator learns of any SAEs that occur after study participation has concluded and the event is deemed relevant to the use of the study drug, he/she should promptly document and report the event to TLC.

All AEs and SAEs will be recorded in the CRF/eCRF database within the timelines outlined in the CRF/eCRF completion guideline.

7.3.1.3. Serious Adverse Event Paper Reporting Process

Within 24 hours of the investigator's knowledge of the event, site personnel must record all SAE data in the eCRF database, from which SAE information will be transmitted to TLC. Detailed instructions can be found in the eCRF completion guidelines.

In addition to eCRF reporting, all SAEs should be recorded on the SAE paper reporting form and reported to TLC within 24 hours of the investigator's knowledge of the event to the email address below:

**TLC Safety:**	E-mail:	safety@tlc-tx.com

For fatal or life-threatening events, copies of hospital case reports, autopsy reports, and other documents are also to be submitted by e-mail when requested and applicable. Transmission of such documents should occur without personal subject identification, maintaining the traceability of a document only to the subject identifiers.

Additional information may be requested to ensure the timely completion of accurate safety reports.

Any medications necessary for the treatment of the SAE must be recorded on the concomitant medication section of the subject's CRF/eCRF and the event description section of the SAE form.

7.4. TLC Reporting Requirements

Depending on relevant local legislation or regulations, including the applicable US FDA Code of Federal Regulations, the European Union Clinical Trials Regulation (536/2014) and relevant updates, and other country-specific legislation or regulations, TLC may be required to expedite to worldwide regulatory agencies report of SAEs, serious adverse drug reactions (SADRs), or Suspected Unexpected Serious Adverse Reaction (SUSARs).

Assessment of expectedness for SAEs will be determined by TLC using reference safety information specified in the IB or relevant local label, as applicable.

All investigators will receive a safety letter notifying them of relevant SUSAR reports associated with any study drug. The investigator should notify the IRB/IEC of SUSAR reports as soon as is practical, where this is required by local regulatory agencies and in accordance with the local institutional policy.

7.5. Toxicity Management

Treatment-emergent toxicities will be noted by the investigator and brought to the attention of the TLC MM, who will decide the appropriate course of action based on a discussion with the investigator. Whether or not considered treatment-related, all subjects experiencing AEs must be monitored periodically until symptoms subside, any abnormal laboratory values have resolved or returned to baseline levels or they are considered irreversible, or until there is a satisfactory explanation for the changes observed (see Appendix 2).

Any questions regarding toxicity management should be directed to the TLC MM.

7.6. Special Situations Reports

7.6.1. Definitions of Special Situations

Special situation reports include all reports of a medication error, abuse, misuse, overdose, reports of AEs associated with product complaints, and pregnancy reports regardless of an associated AE.

Medication error is any unintentional error in the prescribing, dispensing, or administration of a medicinal product while in the control of the health care provider, subject, or consumer.

Abuse is defined as persistent or sporadic intentional excessive use of a medicinal product by a subject.

Misuse is defined as any intentional and inappropriate use of a medicinal product that is not in accordance with the protocol instructions or the local prescribing information.

An overdose is defined as an accidental or intentional administration of a quantity of a medicinal product given per administration or cumulatively which is above the maximum recommended dose as per protocol or in the product labeling (as it applies to the daily dose of the subject in question). In cases of a discrepancy in drug accountability, overdose will be established only when it is clear that the subject has taken the excess dose(s). Overdose cannot be established when the subject cannot account for the discrepancy except in cases in which the investigator has reason to suspect that the subject has taken the additional dose(s).

Product complaint is defined as complaints arising from potential deviations in the manufacture, packaging, or distribution of the medicinal product.

7.6.2. Instructions for Reporting Special Situations

7.6.2.1. Instructions for Reporting Pregnancies

The investigator should report pregnancies in female study subjects that are identified after initiation of study medication and throughout the study, including the post study drug follow-up period. The pregnancy should be recorded on the pregnancy report form and reported to TLC within 24 hours of the investigator becoming aware of the pregnancy to the TLC Safety email address.

Refer to Section 7.3 and the CRF/eCRF completion guidelines for full instructions on the mechanism of pregnancy reporting.

The pregnancy itself is not considered an AE nor is an induced elective abortion to terminate a pregnancy without medical reasons.

Any premature termination of pregnancy (e.g., a spontaneous abortion, an induced therapeutic abortion due to complications or other medical reasons) must be reported within 24 hours as an SAE. The underlying medical reason for this procedure should be recorded as the AE term.

A spontaneous abortion is always considered to be an SAE and will be reported as described in Section 7.3. Furthermore, any SAE occurring as an adverse pregnancy outcome post-study must be reported to TLC.

The subject should receive appropriate monitoring and care until the conclusion of the pregnancy. The outcome should be reported to TLC using the pregnancy outcome report form. If the end of the pregnancy occurs after the study has been completed, the outcome should be reported directly to TLC by emailing the TLC Safety email address.

Refer to Appendix 3 for Pregnancy Precautions, Definition for Female of Childbearing Potential, and Contraceptive Requirements.

7.6.2.2. Reporting Other Special Situations

All other special situation reports must be reported on the special situations report form and forwarded to TLC within 24 hours of the investigator becoming aware of the situation to the TLC Safety email address. These reports must consist of situations that involve study drug.

Special situations involving concomitant medications do not need to be reported on the special situations report form; however, for special situations that result in AEs due to concomitant medication, the AE should be reported on the AE form.

Any inappropriate use of concomitant medications prohibited by this protocol should not be reported as “misuse” but may be more appropriately documented as a protocol deviation.

Refer to Section 7.3 and the CRF/eCRF completion guidelines for full instructions on the mechanism of special situations reporting.

All clinical sequelae in relation to these special situation reports will be reported as AEs or SAEs at the same time using the AE CRF/eCRF and/or the SAE report form. Details of the symptoms and signs, clinical management, and outcome will be reported, when available.

8. STATISTICAL CONSIDERATIONS

8.1. Analysis Objectives and Endpoints

8.1.1. Analysis Objectives

The primary objectives of this study are as follows:

- To evaluate the safety and tolerability of escalating single and multiple oral doses of TLC-2716
- To characterize the single- and multiple-dose pharmacokinetics (PK) of TLC-2716 and its metabolite(s)

The exploratory objectives of this study are as follows:

- To evaluate the pharmacodynamics (PD) of liver X receptor (LXR) inverse agonism by TLC-2716 as measured by metabolic parameters including fasting lipids and serum biomarkers of *de novo* lipogenesis (DNL)
- To characterize TLC-2716 dose and/or exposure-response relationships for PD markers
- To evaluate exploratory biomarkers

8.1.2. Primary Endpoint(s)

The primary endpoints of this study are as follows:

- PK parameters including AUC_{last}, AUC_{inf} [single-dose], $\%AUC_{exp}$ [single dose], CL/F [single dose], AUC_{tau} [multiple-dose], C_{tau} [multiple-dose], CL_{ss}/F [multiple dose], and V_z/F, C_{max}, T_{max}, C_{last}, T_{last}, λ_z, CL/F, and $t_{1/2}$ of TLC-2716 and its metabolite(s), as applicable
- Incidence of AEs
- Incidence of laboratory abnormalities
- 12-lead ECG measurements and abnormalities
- Vital sign measurements
- Physical examinations

8.1.3. Exploratory Endpoint(s)

The exploratory endpoints of this study include:

- PD biomarkers of TLC-2716 and its metabolites, including fasting lipids, serum ANGPTL3, ApoC3, whole blood gene expression of ABCA1 and ABCG1, biomarkers of DNL, and other exploratory biomarkers, as applicable
- Dose and/or exposure-response relationships between PK parameters and PD biomarkers of TLC-2716 and its metabolites, as applicable

8.2. Planned Analyses

8.2.1. Dose Escalation Analysis

For the purpose of making the decision to escalate to the next dose level/cohort, review of all available safety, tolerability, and relevant PK and/or PD data will be conducted after all subjects in each cohort have completed dosing and the necessary follow-up period has been completed, as outlined in Section 3.2. For investigators, safety assessments and relevant PD data will be displayed by cohort (blinded to treatment) to facilitate the decision to dose escalate while preserving the blind at the subject level. The sponsor may separately evaluate unblinded safety, PK, and/or PD data at the cohort and subject level as necessary to support decisions regarding dose escalation and/or design and initiation of adaptive cohorts in Part C.

8.2.2. Final Analysis

The final analysis will be performed after all subjects have completed the study, outstanding data queries have been resolved or adjudicated as unresolvable, and the data have been cleaned and finalized.

8.3. Analysis Conventions

8.3.1. Analysis Sets

8.3.1.1. Safety

The Safety Analysis Set will include all randomized subjects who received at least 1 dose of study drug. Subjects who received treatment other than that to which they were assigned will be analyzed according to the treatment received.

8.3.1.2. Pharmacokinetics

The PK Analysis Set will include all randomized subjects who received at least 1 dose of TLC-2716 and had at least 1 non-missing PK concentration value reported by the PK laboratory for each respective analyte.

8.3.1.3. Pharmacodynamics

Each PD Analysis Set will include all randomized subjects who received at least 1 dose of TLC-2716 and had the necessary baseline and on-study measurements to provide interpretable results for each respective PD parameter.

8.4. Data Handling Conventions

For summary statistics, PK concentration values below the limit of quantitation (BLQ) will be imputed as zero at pre-dose and one-half of the lower limit of quantitation (LLOQ) for post-dose time points.

Laboratory data that are continuous in nature but are less than the LLOQ or above the upper limit of quantitation (ULOQ) will be imputed to the value of the lower or upper limit minus or plus one significant digit, respectively (e.g., if the result of a continuous laboratory test is < 20, a value of 19 will be assigned; if the result of a continuous laboratory test is < 20.0, a value of 19.9 will be assigned).

Missing data can have an impact upon the interpretation of the trial data. As this study is of short duration, it is anticipated that missing data will be minimal. In general, values for missing data will not be imputed. However, a missing pretreatment laboratory result would be treated as normal (i.e., no toxicity grade) for the laboratory abnormality summary.

8.5. Demographic Data and Baseline Characteristics

Demographic and baseline measurements will be summarized, and descriptive statistics will be provided.

8.6. Safety Analysis

All safety data collected on or after the date that study drug was first administered up to the date of last dose of study drug plus 14 days will be summarized by treatment group (according to the study drug received) or treatment sequence using the Safety Analysis Set. As appropriate, placebo-treated subjects will be pooled across Parts A, B, and C of the study.

8.6.1. Extent of Exposure

A subject’s extent of exposure to study drug data will be generated from the study drug administration page in the eCRF. Exposure data will be listed.

8.6.2. Adverse Events

Clinical and laboratory AEs will be coded using the Medical Dictionary for Regulatory Activities (MedDRA). System Organ Class (SOC), High-Level Group Term, High-Level Term, Preferred Term (PT), and Lower-Level Term will be attached to the clinical database.

Adverse event data will be listed by subject. Treatment-emergent AEs (TEAE), serious TEAE, and TEAE leading to discontinuation of treatment (as applicable) will be summarized by treatment, relationship to study drug, severity, as well as SOC, and PT using the current version of the MedDRA.

8.6.3. Laboratory Evaluations

Listings of individual subject laboratory results will be provided. Selected laboratory data will be summarized by cohort at scheduled visits and for the corresponding change from Baseline (Day 1 pre-dose for SAD cohorts; Day -1 for MAD cohorts). The incidence of treatment-emergent, graded laboratory abnormalities will be summarized by cohort and treatment.

8.6.4. Other Safety Evaluations

Vital signs and ECG data will be summarized by treatment sequence.

8.7. Pharmacokinetic Analysis

Plasma concentrations and PK parameters (e.g., AUC_{last}, AUC_{inf} [single-dose], $\%AUC_{exp}$ [single dose], CL/F [single dose], AUC_{tau} [multiple-dose], C_{tau} [multiple-dose], CL_{ss}/F [multiple dose], and V_z/F, C_{max}, T_{max}, C_{last}, T_{last}, λ_z, CL/F, and $t_{1/2,}$ if applicable) will be listed and summarized for TLC-2716 (and its metabolites, as applicable) using descriptive statistics by treatment for each cohort.

Dose proportionality will be assessed by comparing PK parameters of TLC-2716 across evaluated dose levels. A power model will be fitted using all doses across cohorts to evaluate the dose proportionality based on AUC_{last}, AUC_{inf} (single-dose), AUC_{tau} (multiple-dose), C_{tau} (multiple-dose), and C_{max}. The population mean slope will be estimated with a 90% confidence interval. An alternate evaluation of dose proportionality may be conducted using analysis of variance (ANOVA) on dose normalized, natural log-transformed AUC, C_{tau} (multiple-dose), and C_{max}. Dose proportionality analyses will be conducted separately for the single- and multiple-dose cohorts.

Additional analyses such as accumulation ratio and time to steady state may be conducted as appropriate. If fasted cohorts are conducted in Part C, an evaluation of the effect of fasting status on TLC-2716 PK may be conducted.

8.8. Pharmacodynamics Analysis

PD biomarker data, including but not limited to serum ANGPTL3, ApoC3, and markers of DNL (e.g., malonate carnitine index), if applicable, will be listed and summarized using descriptive statistics by cohort and treatment groups at individual sampling time points. Summary statistics may also be provided to characterize the effect of concomitant food intake on the PD biomarkers. Exploratory analyses may be performed to evaluate associations amongst the biomarkers and between the biomarkers and laboratory parameters.

8.9. Pharmacokinetics and Pharmacodynamics Analysis

Dose and/or exposure-response relationships for TLC-2716 with PD biomarkers across all doses will be evaluated, as appropriate.

8.10. Sample Size

Due to its exploratory nature and no reliable variability estimation, no formal power or sample size calculations were used to determine the sample size for this study. Empirically, a sample size of 100-150 subjects (10 subjects per cohort, including 8 active and 2 placebo) was selected to provide an adequate characterization of safety, PK, and PD of TLC-2716.

9. RESPONSIBILITIES

9.1. Investigator Responsibilities

9.1.1. Good Clinical Practice

The investigator will ensure that this study is conducted in accordance with International Council for Harmonization E6(R2) addendum to its guideline for Good Clinical Practice and applicable laws and regulations.

9.1.2. Financial Disclosure

The investigator and sub-investigators will provide prompt and accurate documentation of their financial interest or arrangements with the sponsor or proprietary interests in the study drug. This documentation must be provided before the investigator's (and any sub-investigator's) participation in the study. The investigator and sub-investigator agree to notify TLC of any change in reportable interests during the study and for 1 year following completion of the study. Study completion is defined as the date when the last [subject/patient] completes the protocol-defined activities.

9.1.3. Institutional Review Board/Independent Ethics Committee Review and Approval

The investigator (or TLC as appropriate according to local regulations) will submit this protocol, ICF, and any accompanying material to be provided to the subject (such as advertisements, subject information sheets, or descriptions of the study used to obtain informed consent) to an IRB/IEC. The investigator will not begin any study subject activities until approval from the IRB/IEC has been documented and provided as a letter to the investigator.

Before implementation, the investigator will submit to and receive documented approval from the IRB/IEC for any modifications made to the protocol or any accompanying material to be provided to the subject after initial IRB/IEC approval, with the exception of those necessary to reduce immediate risk to study subject.

9.1.4. Informed Consent

The investigator is responsible for obtaining written informed consent from each individual participating in this study after an adequate explanation of the aims, methods, objectives, and potential hazards of the study and before undertaking any study-related procedures. The investigator must use the most current IRB/IEC-approved ICF for documenting written informed consent. Each ICF will be appropriately signed and dated by the subject or the subject's legally authorized representative and the person conducting the consent discussion, and also by an impartial witness if required by IRB/IEC requirements.

The ICF will inform subjects about genomic testing and/or planned sample retention. In addition to the study-specific ICF to be signed by each subject participating in the study, subjects will be

required to document additional consent to provide additional samples and/or to allow the use of the remainder of their already-collected specimens for optional future research, in accordance with applicable regulations. In addition to the study-specific informed consent form to be signed by each subject participating in the study, subjects will be required to document additional consent to provide additional samples for optional genomic research. The results of the tests performed on the samples will not be given to the subject or the investigator. The stored biological samples will be destroyed no later than 15 years after the end of the study or per country requirements, but subjects may at any time request that their stored samples be destroyed.

9.1.5. Confidentiality

The investigator must assure that subjects' anonymity will be strictly maintained and that their identities are protected from unauthorized parties. Only subject initials, date of birth, another unique identifier (as allowed by local law), and an identification code will be recorded on any form or biological sample submitted to the sponsor, IRB/IEC, or laboratory. Laboratory specimens must be labeled in such a way as to protect subject identity while allowing the results to be recorded to the proper subject. Refer to specific laboratory instructions or in accordance with local regulations.

NOTE: The investigator must keep a screening log showing codes, names, and addresses for all subjects screened and for all subjects enrolled in the trial. Subject data will be processed in accordance with all applicable regulations.

The investigator agrees that all information received from TLC, including but not limited to the IB, this protocol, CRF/eCRF, the study drug, and any other study information, remain the sole and exclusive property of TLC during the conduct of the study and thereafter. This information is not to be disclosed to any third party (except employees or agents directly involved in the conduct of the study or as required by law) without prior written consent from TLC. The investigator further agrees to take all reasonable precautions to prevent the disclosure by any employee or agent of the study site to any third party or otherwise into the public domain.

9.1.6. Study Files and Retention of Records

The investigator must maintain adequate and accurate records to enable the conduct of the study to be fully documented and the study data to be subsequently verified. These documents should be classified into at least the following two categories: (1) investigator's study file, and (2) subject clinical source documents.

The investigator's study file will contain the protocol/amendments, CRF and query forms, IRB/IEC, and governmental approval with correspondence, informed consent, drug records, staff curriculum vitae, authorization forms, and other appropriate documents and correspondence.

The required source data should include sequential notes containing at least the following information for each subject:

- Subject identification (name, date of birth, gender)

- Documentation that the subject meets eligibility criteria (i.e., history, physical examination, and confirmation of diagnosis) to support inclusion and exclusion criteria
- Documentation of the reason(s) a consented subject is not enrolled
- Participation in the study (including study number)
- The study discussed and date of informed consent
- Dates of all visits
- Documentation that protocol-specific procedures were performed
- Results of efficacy parameters, as required by the protocol
- Start and end date (including dose regimen) of study drug, including dates of dispensing and return
- Record of all the AEs and other safety parameters (start and end date, including causality and severity)
- Concomitant medication (including start and end date, dose if relevant, dose changes)
- Date of study completion and reason for early discontinuation, if it occurs.

All clinical study documents must be retained by the investigator until at least 2 years or according to local laws, whichever is longer, after the last approval of a marketing application in an ICH region (i.e., United States, Europe, or Japan) and until there are no pending or planned marketing applications in an ICH region; or, if no application is filed or if the application is not approved for such indication until 2 years after the investigation is discontinued and regulatory authorities have been notified. Investigators may be required to retain documents longer if specified by regulatory requirements, by local regulations, or by an agreement with TLC. The investigator must notify TLC before destroying any clinical study records.

Should the investigator wish to assign the study records to another party or move them to another location, TLC must be notified in advance.

If the investigator cannot provide for this archiving requirement at the study site for any or all of the documents, special arrangements must be made between the investigator and TLC to store these records securely away from the site so that they can be returned sealed to the investigator in case of an inspection. When source documents are required for the continued care of the subject, appropriate copies should be made for storage away from the site.

9.1.7. Case Report Forms

For each subject enrolled, an eCRF will be completed by an authorized study staff member whose training for this function is documented according to study procedures. eCRF should be completed on the day of the subject visit to enable the sponsor to perform central monitoring of safety data. The Eligibility Criteria eCRF should be completed only after all data related to eligibility have been received and the subject has been enrolled. Subsequent to data entry, a study monitor will perform source data verification within the electronic data capture system.

Original entries as well as any changes to data fields will be stored in the audit trail of the system. Prior to database lock (or any interim time points as described in the clinical data management plan), the investigator will use his/her log-in credentials to confirm that the forms have been reviewed and that the entries accurately reflect the information in the source documents. The eCRF captures the data required per the protocol schedule of events and procedures. System-generated or manual queries will be issued to the investigative site staff as data discrepancies are identified by the monitor or internal TLC staff, who routinely review the data for completeness, correctness, and consistency. The site coordinator is responsible for responding to the queries in a timely manner, within the system, either by confirming the data as correct or updating the original entry and providing the reason for the update (e.g., data entry error). At the conclusion of the trial, TLC will provide the site with a read-only archive copy of the data entered by that site. This archive must be stored in accordance with the records retention requirements outlined in Section 9.1.6.

9.1.8. Study Drug Accountability and Return

TLC recommends that used and unused study drug supplies be returned to the shipping facility from which it came. The study monitor will provide instructions for return. If return is not possible, the study monitor will evaluate each study center’s study drug disposal procedures and provide appropriate instruction for the return and possible destruction of unused study drug supplies. If the site has an appropriate SOP for drug destruction as determined by TLC, the site may destroy used (empty or partially empty) and any unused study drug supplies in accordance with that site’s approved SOP. A copy of the site’s approved SOP will be obtained for central files.

If the study drug is destroyed on-site, the investigator must maintain accurate records for all the study drug destroyed. Records must show the identification and quantity of each unit destroyed, the method of destruction, and the person who disposed of the study drug. Upon study completion, copies of the study drug accountability records must be filed at the site. Another copy will be returned to TLC.

The study monitor will review study drug supplies and associated records at periodic intervals.

9.1.9. Inspections

The investigator will make available all source documents and other records for this trial to TLC’s appointed study monitors, to IRB/IEC, or to a regulatory authority or health authority inspectors.

9.1.10. Protocol Compliance

The investigator is responsible for ensuring the study is conducted in accordance with the procedures and evaluations described in this protocol.

9.2. Sponsor Responsibilities

9.2.1. Protocol Modifications

Protocol modifications, except those intended to reduce immediate risk to study subjects, may be made only by TLC. The investigator must submit all protocol modifications to the IRB/IEC in accordance with local requirements and receive documented IRB/IEC approval before modifications can be implemented.

9.2.2. Study Report

A clinical study report will be prepared and provided to the regulatory agency(ies). TLC will ensure that the report meets the standards set out in the ICH Guideline for Structure and Content of Clinical Study Reports (ICH E3). Note that an abbreviated report may be prepared in certain cases.

9.3. Joint Investigator/Sponsor Responsibilities

9.3.1. Payment Reporting

Investigators and their study staff may be asked to provide services performed under this protocol (e.g., attendance at Investigator's Meetings). If required under the applicable statutory and regulatory requirements, TLC will capture and disclose to federal and state agencies any expenses paid or reimbursed for such services, including any clinical trial payments, meals, travel expenses or reimbursements, consulting fees, and any other transfer of value.

9.3.2. Access to Information for Monitoring

In accordance with regulations and guidelines, the study monitor must have direct access to the investigator’s source documentation in order to verify the accuracy of the data recorded in the CRF/eCRF.

The monitor is responsible for routine review of the CRF/eCRF at regular intervals throughout the study to verify adherence to the protocol and the completeness, consistency, and accuracy of the data being entered into them. The monitor should have access to any subject records needed to verify the entries on the CRF/eCRF. The investigator agrees to cooperate with the monitor to ensure that any problems detected through any type of monitoring (central, on-site) are resolved.

9.3.3. Access to Information for Auditing or Inspections

Representatives of regulatory authorities or TLC may conduct inspections or audits of the clinical study. If the investigator is notified of an inspection by a regulatory authority the investigator agrees to notify the TLC MM immediately. The investigator agrees to provide representatives of a regulatory agency or TLC access to records, facilities, and personnel for the effective conduct of any inspection or audit.

9.3.4. Study Discontinuation

Both the sponsor and the investigator reserve the right to terminate the study at any time. Should this be necessary, both parties will arrange discontinuation procedures and notify the appropriate regulatory authority(ies), IRBs, and IECs. In terminating the study, TLC and the investigator will assure that adequate consideration is given to the protection of the subjects' interests.

10. REFERENCES

Ahmad, Z., R. Pordy, D. J. Rader, D. Gaudet, S. Ali, C. Gonzaga-Jauregui, M. P. Ponda, B. Shumel, P. Banerjee and R. L. Dunbar (2021). "Inhibition of Angiopoietin-Like Protein 3 With Evinacumab in Subjects With High and Severe Hypertriglyceridemia." J Am Coll Cardiol **78**(2): 193-195.

Beheshti, S. O., C. M. Madsen, A. Varbo and B. G. Nordestgaard (2020). "Worldwide Prevalence of Familial Hypercholesterolemia: Meta-Analyses of 11 Million Subjects." J Am Coll Cardiol **75**(20): 2553-2566.

Bouhairie, V. E. and A. C. Goldberg (2015). "Familial hypercholesterolemia." Cardiol Clin 33(2): 169-179.

Cuchel, M., E. A. Meagher, H. du Toit Theron, D. J. Blom, A. D. Marais, R. A. Hegele, M. R. Averna, C. R. Sirtori, P. K. Shah, D. Gaudet, C. Stefanutti, G. B. Vigna, A. M. Du Plessis, K. J. Propert, W. J. Sasiela, L. T. Bloedon, D. J. Rader and F. H. L. S. i. Phase 3 Ho (2013). "Efficacy and safety of a microsomal triglyceride transfer protein inhibitor in patients with homozygous familial hypercholesterolaemia: a single-arm, open-label, phase 3 study." Lancet 381(9860): 40-46.

Defesche, J. C., S. S. Gidding, M. Harada-Shiba, R. A. Hegele, R. D. Santos and A. S. Wierzbicki (2017). "Familial hypercholesterolaemia." Nat Rev Dis Primers 3: 17093.

Griffett, K., L. A. Solt, D. El-Gendy Bel, T. M. Kamenecka and T. P. Burris (2013). "A liver-selective LXR inverse agonist that suppresses hepatic steatosis." ACS Chem Biol 8(3): 559-567.

Griffett, K., R. D. Welch, C. A. Flaveny, G. R. Kolar, B. A. Neuschwander-Tetri and T. P. Burris (2015). "The LXR inverse agonist SR9238 suppresses fibrosis in a model of non-alcoholic steatohepatitis." Mol Metab 4(4): 353-357.

Hambruch, E., U. Deuschle, C. Gege, O. Kinzel, M. Albers, D. H. Krol, M. Birkel and C. Kremoser (2017). LXR inverse agonists demonstrate liver lipid lowering effects through multiple mechanisms in rodent models of NASH and in human hepatocytes. The International Liver Congress. Amsterdam, The Netherlands.

Janowski, B. A., M. J. Grogan, S. A. Jones, G. B. Wisely, S. A. Kliewer, E. J. Corey and D. J. Mangelsdorf (1999). "Structural requirements of ligands for the oxysterol liver X receptors LXRalpha and LXRbeta." Proc Natl Acad Sci U S A 96(1): 266-271.

Janowski, B. A., P. J. Willy, T. R. Devi, J. R. Falck and D. J. Mangelsdorf (1996). "An oxysterol signalling pathway mediated by the nuclear receptor LXR alpha." Nature 383(6602): 728-731.

Lambert, J. E., M. A. Ramos-Roman, J. D. Browning and E. J. Parks (2014). "Increased de novo lipogenesis is a distinct characteristic of individuals with nonalcoholic fatty liver disease." Gastroenterology 146(3): 726-735.

Lang, W. and W. H. Frishman (2019). "Angiopoietin-Like 3 Protein Inhibition: A New Frontier in Lipid-Lowering Treatment." Cardiol Rev 27(4): 211-217.

Lawitz, E. J., A. Coste, F. Poordad, N. Alkhouri, N. Loo, B. J. McColgan, J. M. Tarrant, T. Nguyen, L. Han, C. Chung, A. S. Ray, J. G. McHutchison, G. M. Subramanian, R. P. Myers, M. S. Middleton, C. Sirlin, R. Loomba, E. Nyangau, M. Fitch, K. Li and M. Hellerstein (2018). "Acetyl-CoA Carboxylase Inhibitor GS-0976 for 12 Weeks Reduces Hepatic De Novo Lipogenesis and Steatosis in Patients With Nonalcoholic Steatohepatitis." Clin Gastroenterol Hepatol 16(12): 1983-1991.e1983.

Luirink, I. K., J. Determeijer, B. A. Hutten, A. Wiegman, E. Bruckert, C. P. Schmitt and J. W. Groothoff (2019). "Efficacy and safety of lipoprotein apheresis in children with homozygous familial hypercholesterolemia: A systematic review." J Clin Lipidol 13(1): 31-39.

McGowan, M. P., S. H. Hosseini Dehkordi, P. M. Moriarty and P. B. Duell (2019). "Diagnosis and Treatment of Heterozygous Familial Hypercholesterolemia." J Am Heart Assoc 8(24): e013225.

Neuschwander-Tetri, B. A. (2010). "Hepatic lipotoxicity and the pathogenesis of nonalcoholic steatohepatitis: the central role of nontriglyceride fatty acid metabolites." Hepatology 52(2): 774-788.

Ong, J. P. and Z. M. Younossi (2007). "Epidemiology and natural history of NAFLD and NASH." Clin Liver Dis 11(1): 1-16, vii.

Raal, F. J., G. K. Hovingh and A. L. Catapano (2018). "Familial hypercholesterolemia treatments: Guidelines and new therapies." Atherosclerosis 277: 483-492.

Raal, F. J., R. S. Rosenson, L. F. Reeskamp, G. K. Hovingh, J. J. P. Kastelein, P. Rubba, S. Ali, P. Banerjee, K. C. Chan, D. A. Gipe, N. Khilla, R. Pordy, D. M. Weinreich, G. D. Yancopoulos, Y. Zhang and D. Gaudet (2020). "Evinacumab for Homozygous Familial Hypercholesterolemia." N Engl J Med 383(8): 711-720.

Santos, R. D., S. S. Gidding, R. A. Hegele, M. A. Cuchel, P. J. Barter, G. F. Watts, S. J. Baum, A. L. Catapano, M. J. Chapman, J. C. Defesche, E. Folco, T. Freiberger, J. Genest, G. K. Hovingh, M. Harada-Shiba, S. E. Humphries, A. S. Jackson, P. Mata, P. M. Moriarty, F. J. Raal, K. Al-Rasadi, K. K. Ray, Z. Reiner, E. J. Sijbrands, S. Yamashita and P. International Atherosclerosis Society Severe Familial Hypercholesterolemia (2016). "Defining severe familial hypercholesterolaemia and the implications for clinical management: a consensus statement from the International Atherosclerosis Society Severe Familial Hypercholesterolemia Panel." Lancet Diabetes Endocrinol 4(10): 850-861.

Scherer, J., V. P. Singh, C. S. Pitchumoni and D. Yadav (2014). "Issues in hypertriglyceridemic pancreatitis: an update." J Clin Gastroenterol 48(3): 195-203.

Tarugi, P., S. Bertolini and S. Calandra (2019). "Angiopoietin-like protein 3 (ANGPTL3) deficiency and familial combined hypolipidemia." J Biomed Res 33(2): 73-81.

Underberg, J. A., C. P. Cannon, D. Larrey, L. Makris, D. Blom and H. Phillips (2020). "Long-term safety and efficacy of lomitapide in patients with homozygous familial hypercholesterolemia: Five-year data from the Lomitapide Observational Worldwide Evaluation Registry (LOWER)." J Clin Lipidol 14(6): 807-817.

Vernon, G., A. Baranova and Z. M. Younossi (2011). "Systematic review: the epidemiology and natural history of non-alcoholic fatty liver disease and non-alcoholic steatohepatitis in adults." Aliment Pharmacol Ther 34(3): 274-285.

11. APPENDICES

Appendix 1. Investigator Signature Page

Appendix 2. Management of Clinical and Laboratory Adverse Events

Appendix 3. Pregnancy Precautions, Definition for Female of Childbearing Potential, and Contraceptive Requirements

Appendix 4. Common Terminology Criteria for Adverse Events (CTCAE) Grading Scale v5.0

Appendix 1. Investigator Signature Page

THE LIVER COMPANY, INC.
2671 Marshall Drive
Palo Alto, CA, 94303, USA

STUDY ACKNOWLEDGEMENT

A Phase 1 Study to Evaluate the Safety, Tolerability, Pharmacokinetics, and Pharmacodynamics of Single and Multiple Ascending Doses of TLC-2716 in Healthy Subjects

2716-CL-101, Protocol Amendment 1: 04 August 2022

This protocol has been approved by The Liver Company, Inc. The following signature documents this approval.

Ryan S. Huss, MD (Printed)<br>Vice President, Clinical Development & Operations	Signature

Date

INVESTIGATOR STATEMENT

I have read the protocol, including all appendices, and I agree that it contains all the necessary details for me and my staff to conduct this study as described. I will conduct this study as outlined herein and will make a reasonable effort to complete the study within the time designated.

I will provide all study personnel under my supervision copies of the protocol and access to all information provided by The Liver Company, Inc. I will discuss this material with them to ensure that they are fully informed about the drugs and the study.

Principal Investigator Name (Printed)	Signature

Date

Appendix 2. Management of Clinical and Laboratory Adverse Events

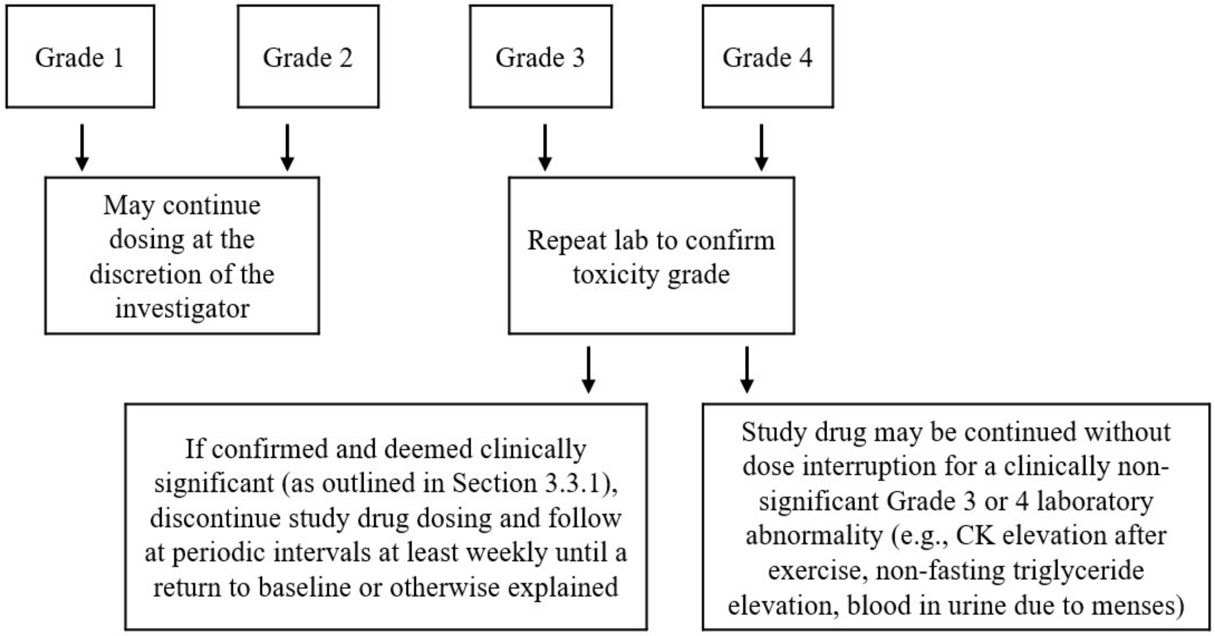

Appendix 3. Pregnancy Precautions, Definition for Female of Childbearing Potential, and Contraceptive Requirements

1) Study Drug Effects on Pregnancy and Hormonal Contraception

The risks of treatment with TLC-2716 during pregnancy have not been evaluated and reproductive and developmental toxicity studies have not been conducted. Clinical data regarding potential drug-drug interactions (DDI) between TLC-2716 and hormones used for contraception are not available. However, *in vitro* data suggest that TLC-2716 is an inducer of CYP3A4, which may affect the efficacy or hormonal contraceptives. Therefore, hormone-containing contraceptives should not be considered an effective form of contraception while used concurrently with TLC-2716. Pregnancy must be excluded before the start of treatment with the study drug and prevented thereafter by reliable, highly effective contraceptive methods. Pregnancy tests will be performed regularly throughout this study. Please refer to the latest version of the IB for additional information.

2) Definition of Female of Childbearing Potential

For the purposes of this study, a female-born subject is considered of childbearing potential following the initiation of puberty (Tanner stage 2) until becoming post-menopausal, unless permanently sterile or with medically documented ovarian failure.

Females are considered to be in a postmenopausal state when they are ≥ 54 years of age with cessation of previously occurring menses for ≥ 12 months without an alternative cause.

In addition, females of any age with amenorrhea of > 12 months may also be considered postmenopausal if their follicle-stimulating hormone (FSH) level is in the postmenopausal range and they are not using hormonal contraception or hormonal replacement therapy.

Permanent sterilization includes hysterectomy, bilateral oophorectomy, or bilateral salpingectomy in a female subject of any age.

3) Definition of Male Fertility

For the purposes of this study, a male-born subject is considered fertile after the initiation of puberty unless permanently sterile by bilateral orchidectomy or medical documentation.

4) Contraception Requirements for Female Subjects of Childbearing Potential

The inclusion of female subjects of childbearing potential requires the use of highly effective contraceptive measures. Given potential drug-drug interactions involving TLC-2716, including with hormone-containing contraceptives, have not been evaluated, subjects must not rely solely on hormone-containing contraceptives as a form of birth control during the study.

Subjects must have a negative serum pregnancy test at Screening and a negative pregnancy test on Day -1 prior to randomization. In the event of a delayed menstrual period (over one month between menstruations), a pregnancy test must be performed to rule out pregnancy. This is also true for women of childbearing potential with infrequent or irregular periods. Female subjects must agree to one of the following methods of contraception from Screening until 30 days after the last dose of study drug:

- Complete abstinence from intercourse of reproductive potential. Abstinence is an acceptable method of contraception only when it is in line with the subject's preferred and usual lifestyle.

Or

Consistent and correct use of 1 of the following methods of birth control listed below:

- Intrauterine device (IUD) with a failure rate of < 1% per year
- Implant contraceptive
- Tubal sterilization (e.g., bilateral tubal ligation)
- Essure micro-insert system (provided confirmation of success 3 months after procedure)
- Vasectomy in the male partner (provided that the partner is the sole sexual partner and had confirmation of surgical success 3 months after procedure)

Or

Consistent and correct use of 1 of the following methods, in addition to use of a barrier method (male condoms, female condoms, or female diaphragm), as appropriate:

- Oral contraceptive
- Injectable contraceptive (e.g., Depo-Provera)
- Vaginal contraceptive ring

Note, barrier methods alone are not an acceptable form of contraception.
Female subjects must also refrain from egg donation and in vitro fertilization during treatment and until at least 90 days after the last dose of study drug.

5) Contraception Requirements for Male Subjects

It is unknown if a relevant systemic concentration of the study drug could be achieved in a female partner from exposure to the male subject's seminal fluid. Therefore, male subjects with female partners of childbearing potential must use condoms during treatment and until 90 days after the last dose of study drug. Additional contraception recommendations should also be considered if the female partner is not pregnant.

Male subjects must also refrain from sperm donation during treatment and until at least 90 days after the last dose of study drug.

6) Unacceptable Birth Control Methods

Birth control methods that are unacceptable include hormone-containing contraceptives, periodic abstinence (e.g., calendar, ovulation, symptothermal, post-ovulation methods), withdrawal (coitus interruptus), spermicides only, and lactational amenorrhea method (LAM). A female condom and a male condom should not be used together.

7) Procedures to be Followed in the Event of Pregnancy

Subjects will be instructed to notify the investigator if they become pregnant at any time during the study, or if they become pregnant within 30 days (for female subjects) or 90 days (for female partners of male subjects) of the last study drug dose. Subjects who become pregnant or who suspect that they are pregnant during the study must report the information to the investigator and discontinue the study drug immediately. Subjects whose partner has become pregnant or suspects she is pregnant during the study must report the information to the investigator. Instructions for reporting pregnancy, partner pregnancy, and pregnancy outcome are outlined in Section 7.6.2.1.

Appendix 4. Common Terminology Criteria for Adverse Events (CTCAE) Grading Scale v5.0

https://ctep.cancer.gov/protocoldevelopment/electronic_applications/docs/CTCAE_v5_Quick_Reference_8.5x11.pdf

STATISTICAL ANALYSIS PLAN

**Study Title:**	A Phase 1 Study to Evaluate the Safety, Tolerability, Pharmacokinetics, and Pharmacodynamics of Single and Multiple Ascending Doses of TLC-2716 in Healthy Subjects
**Investigational Medicinal Product(s):**	TLC-2716
**Study Number:**	2716-CL-101
**Protocol Version:**	Amendment 1, 04 August 2022
**Analysis Type:**	Final Analysis for Parts A, B, and C
**Analysis Plan Version:**	1.0, 19 April 2023
**Analysis Plan Author(s):**	[redacted]

CONFIDENTIAL AND PROPRIETARY INFORMATION

This document is the property of OrsoBio, Inc. It is intended for restricted use only and may not - in full or part - be passed on, reproduced, published or used without express permission of OrsoBio, Inc.

REVISION HISTORY

SAP Version	Date of SAP Version	Summary of Revision	Reason for Revision
1.0	19 April 2023	N/A	N/A – Original SAP

TABLE OF CONTENTS

SIGNATURE PAGE 2
REVISION HISTORY 3
TABLE OF CONTENTS 4
LIST OF ABBREVIATIONS 6
1. INTRODUCTION 8
1.1. Study Objectives 8
1.2. Study Design 8
1.2.1. Part A: Single-Ascending Dose [SAD] (Cohorts 1-5) 8
1.2.2. Part B: Multiple-Ascending Dose [MAD] (Cohorts 6-10) 9
1.2.3. Part C: Adaptive SAD and/or MAD (Cohorts 11-15) 10
1.3. Study Blinding 10
1.4. Randomization 11
1.4.1. Part A (SAD), Cohorts 1-5 11
1.4.2. Part B (MAD), Cohorts 6-10 11
1.4.3. Part C (Adaptive SAD and/or MAD), Cohorts 11-15 11
1.5. Sample Size 11
1.5.1. Part A (SAD), Cohort 1-5 11
1.5.2. Part B (MAD), Cohort 6-10 12
1.5.3. Part C (Adaptive SAD and/or MAD), Cohorts 11-15 12
1.6. Study Intervention 12
1.6.1. Part A (SAD), Cohorts 1-5 12
1.6.2. Part B (MAD), Cohorts 6-10 12
1.6.3. Part C (Adaptive SAD and/or MAD), Cohorts 11-15 13
2. ANALYSIS POPULATIONS 14
2.1. All Randomized Analysis Set 14
2.2. Safety Analysis Set 14
2.3. Pharmacokinetic Analysis Set 14
3. STATISTICAL CONSIDERATIONS FOR DATA ANALYSES AND HANDLINGS 15
3.1. Statistical Reporting 15
3.2. Confidence Interval 15
3.3. Analysis Visit Windows 15
3.3.1. Data Handling Conventions and Transformations 15
3.4. Visit Windows 16
3.4.1. Definition of Predose and Postdose 16
3.4.2. Analysis Visit Windows 17
3.4.3. Selection of Data in the Event of Multiple Records on the Same Day 17
3.5. Missing Data 18
3.6. Outliers 18
3.7. Definitions 18
3.7.1. Study Day 18
3.7.2. Baseline 18
4. DEMOGRAPHIC AND OTHER BASELINE CHARACTERISTICS 19
4.1. Subject Disposition 19
4.2. Demographic and Baseline Characteristics 19
4.3. Medical History 19
5. STUDY CONDUCT 20

5.1. Prior and Concomitant Medications/Therapies 20
5.2. Treatment Exposure and Compliance 20
6. EFFICACY ANALYSES 21
7. SAFETY 22
7.1. Adverse Events and Deaths 22
7.1.1. Adverse Event Dictionary 22
7.1.2. Adverse Event Severity 22
7.1.3. Relationship of Adverse Events to Study Drug 22
7.1.4. Relationship of Adverse Events to Study Procedure 22
7.1.5. Serious Adverse Events 22
7.1.6. Treatment-Emergent Adverse Events 22
7.1.6.1. Definition of Treatment Emergent 22
7.1.6.2. Incomplete Dates 23
7.1.6.3. Summaries of Adverse Events and Deaths 23
7.2. Laboratory Evaluations 24
7.2.1. Summaries of Numeric Laboratory Results 25
7.2.2. Graded Laboratory Values 25
7.2.2.1. Summaries of Laboratory Abnormalities 25
7.2.3. Vital Signs 26
7.2.4. 12-lead Electrocardiograms 26
8. PHARMACOKINETIC ANALYSIS 27
8.1.1. Data Handling 27
8.1.2. Plasma Pharmacokinetic Concentrations 27
8.1.3. Plasma Pharmacokinetic Parameters 28
8.1.4. Pharmacokinetic Statistical Analyses 29
8.1.4.1. Dose Proportionality 29
8.1.5. Evaluation of Food Effect 29
8.1.6. Evaluation of Accumulation 29
9. PHARMACODYNAMIC ANALYSIS 30
10. INTERIM ANALYSES 31
11. PROGRAMMING CONVENTIONS 32
11.1. Formatting and Programming Rule 32
12. REFERENCES 33
13. APPENDICES 34

LIST OF ABBREVIATIONS

AE	adverse event
ALT	alanine aminotransferase
AST	aspartate aminotransferase
AUC_{inf}	area under the concentration versus time curve extrapolated to infinite time
AUC_{last}	area under the concentration versus time curve from time zero to the last quantifiable concentration
AUC_{tau}	area under the concentration versus time curve over the dosing interval
BLQ	below the limit of quantitation
BMI	body mass index
CI	confidence interval
C_{last}	last observed quantifiable concentration of the drug
CL/F	apparent oral clearance after administration of the drug: $CL/F = Dose/AUC_{inf}$, where "Dose" is the dose of the drug
C_{max}	maximum observed concentration of drug
CRF	case report form
CSR	clinical study report
C_{tau}	observed drug concentration at the end of the dosing interval
CTCAE	Common Terminology Criteria for Adverse Events
DNL	*de novo* lipogenesis
ECG	electrocardiogram
ET	early termination
HDL	high-density lipoprotein
HLGT	high-level group term
HLT	high-level term
LDL	low-density lipoprotein
LLT	lower-level term
LOQ	limit of quantitation
LXR	liver X receptor
MedDRA	Medical Dictionary for Regulatory Activities
PD	pharmacodynamic(s)
PK	pharmacokinetic(s)
PT	preferred term
PTM	placebo-to-match
Q1, Q3	first quartile, third quartile
QRS	electrocardiographic deflection between the beginning of the Q wave and termination of the S wave representing time for ventricular depolarization
QT	electrocardiographic interval between the beginning of the Q wave and termination of the T wave representing the time for both ventricular depolarization and repolarization to occur
QTc	QT interval corrected for heart rate

RR	electrocardiographic interval representing the time measurement between the R wave of one heartbeat and the R wave of the preceding heartbeat
SAE	serious adverse event
SAP	statistical analysis plan
SD	standard deviation
SI (units)	international system of units
SOC	system organ class
$t_{1/2}$	estimate of the terminal elimination half-life of the drug, calculated by dividing the natural log of 2 by the terminal elimination rate constant ($\square_z$)
TE	treatment-emergent
TEAE	treatment-emergent adverse event
TFLs	tables, figures, and listings
T_{last}	time (observed time point) of C_{last}
T_{max}	time (observed time point) of C_{max}
ULN	upper limit of normal
WHO	World Health Organization

1. INTRODUCTION

This statistical analysis plan (SAP) describes the statistical methods and data handling to be employed for the analysis of Parts A, B, and C for study 2716-CL-101. This SAP is based on the study protocol Amendment 1, dated 04 August 2022.

All analyses described in the SAP will be performed by OrsoBio, Inc, or a designee. Any changes made after the finalization of the SAP will be documented in the clinical study report (CSR).

1.1. Study Objectives

The primary objectives of this study are as follows:

- To evaluate the safety and tolerability of escalating single and multiple doses of TLC-2716
- To characterize the single- and multiple-dose pharmacokinetics (PK) of TLC-2716 and its metabolite(s)

The exploratory objectives of this study are as follows:

- To evaluate the pharmacodynamics (PD) of liver X receptor (LXR) inverse agonism by TLC-2716 as measured by metabolic parameters including fasting lipids and serum biomarkers of *de novo* lipogenesis (DNL)
- To characterize TLC-2716 dose and/or exposure-response relationships for PD markers
- To evaluate exploratory biomarkers

1.2. Study Design

This is a Phase 1, randomized, double-blind, placebo-controlled, first-in-human study where Parts A, B, and C will evaluate the safety, tolerability, PK, and PD of single- and multiple-ascending doses of TLC-2716 in healthy subjects. Adult male and non-pregnant, non-lactating female subjects 18 to 55 years of age, inclusive, will be enrolled in the study (refer to the protocol for complete inclusion and exclusion criteria). The study employs a design where study drug administration will start at lower doses and proceed to higher doses, after confirming the safety and tolerability at the completion of each cohort.

1.2.1. Part A: Single-Ascending Dose [SAD] (Cohorts 1-5)

Part A will evaluate the safety, tolerability, PK, and PD of single-ascending doses of oral TLC-2716 or placebo-to-match (PTM) under fed conditions. Part A will proceed in up to 5 dose-escalation cohorts and will be governed by a review of safety and tolerability, any relevant and available PK and/or PD data, and study-specific stopping criteria.

Within each cohort, 10 unique subjects will be randomized 4:1 to receive either blinded TLC-2716 (N = 8) or PTM (N = 2). All study drugs in Part A will be administered in the morning following a standard meal.

At the initiation of each cohort in Part A, prior to randomization of the entire cohort, two sentinel subjects will be randomized, one to TLC-2716 at the dose to be evaluated in the planned cohort, and one to corresponding PTM. Enrollment and randomization of the remaining eight subjects in each cohort will be determined upon evaluation of all safety and tolerability data through Day 2 for these two sentinel subjects.

For Cohorts 2-5, initiation of single-dose administration will be permitted after evaluation of cumulative blinded safety data, and any relevant and available PK and/or PD data up to and including Day 4, following single-dose administration of the previous cohort.

The cohorts and study treatments for Part A are as follows:

Cohort	Day 1
1	0.5 mg TLC-2716 (1 x 0.5 mg) or PTM capsule SD, fed
2	2 mg TLC-2716[a] (1 x 2 mg) or PTM capsule SD, fed
3	6 mg TLC-2716[a] (3 x 2 mg) or PTM capsules SD, fed
4	12 mg TLC-2716[a] (2 x 5 mg; 1 x 2 mg) or PTM capsules SD, fed
5	20 mg TLC-2716[a] (4 x 5 mg) or PTM capsules SD, fed

PTM = placebo-to-match; SD = single dose.
[a] The planned doses may be modified based on the observed safety, tolerability, and any relevant and available PK and/or PD data. Any modified dose will be within 3-fold of a dose previously tested.

1.2.2. Part B: Multiple-Ascending Dose [MAD] (Cohorts 6-10)

Part B will proceed in up to 5 dose-escalation cohorts and will be governed by reviews of safety and tolerability, and any relevant and available PK and/or PD data, and study-specific stopping criteria. Within each cohort, 10 unique subjects will be randomized 4:1 to receive either blinded TLC-2716 (N = 8) or PTM (N = 2) QD for 14 days.

All study drugs in Part B will be administered in the morning following a standard meal.

Part B (Cohort 6) may be initiated after the evaluation of cumulative safety, tolerability, and any relevant and available PK and/or PD data from all subjects enrolled in Cohort 3 in Part A (up to and including Day 4).

For Cohorts 7-10, dosing may be initiated after evaluation of cumulative safety, tolerability, and any relevant and available PK and/or PD data from all subjects enrolled in the previous cohort in Part B through Day 14.

In both Parts A and B, escalation to a dose higher than previously studied may occur only in the absence of dose-limiting toxicity and/or not meeting any prespecified stopping criteria.

The cohorts and study treatments for Part B are as follows:

Cohort	Day -1	Days 1-14
6	PTM capsule SD	0.5 mg TLC-2716 (1 x 0.5 mg) or PTM capsule, fed
7	PTM capsule SD	2 mg TLC-2716[a] (1 x 2 mg) or PTM capsule, fed

Cohort	Day -1	Days 1-14
**8**	PTM capsules SD	6 mg TLC-2716 [a] (3 x 2 mg) or PTM capsules, fed
**9**	PTM capsules SD	12 mg TLC-2716 [a] (2 x 5 mg; 1 x 2 mg) or PTM capsules, fed
**10**	PTM capsules SD	20 mg TLC-2716 [a] (4 x 5 mg) or PTM capsules, fed

PTM = placebo-to-match; SD = single dose.
[a] The planned doses may be modified based on the observed safety, tolerability, and any relevant and available PK and/or PD data. Any modified dose will be within 3-fold of a dose previously tested.

1.2.3. Part C: Adaptive SAD and/or MAD (Cohorts 11-15)

Based on available safety, PK, and/or PD data from Part A and Part B (if applicable), doses for Part C (Cohorts 11-15) will be chosen up to a total daily dose of 50 mg. The frequency of dosing (QD or twice daily [BID]), duration of dosing (single on one day or multiple over 14 days), and fasting versus fed status may also be modified versus Parts A and B. If dosing is administered in the fed state, standard meal conditions may also be modified at sponsor discretion. QD doses will be administered in the morning following a standard meal, and BID doses will be administered after a standard morning meal and evening snack, with the second dose administered 12 hours (± 10 minutes) after the morning dose. Once determined, dose level, duration of dosing, frequency of dosing, and meal conditions will remain consistent within a cohort. For any multiple-dose cohort in Part C with BID dosing, both morning and evening doses must be administered on Day 14.

Within each cohort, 10 unique subjects will be randomized 4:1 to receive up to a total daily dose of 50 mg TLC-2716 (N = 8) or PTM (N = 2) for either 1 day (as in Part A) or 14 days (as in Part B).

Adaptive, multiple-dose cohorts in Part C may be initiated in parallel with previous cohorts if the total daily dose under evaluation is at or below a dose already evaluated in Part B. If doses chosen in 2 or more adaptive cohorts exceed the dose evaluated in a previous cohort in Part B, these cohorts will be conducted in a staggered manner (lowest dose first), with the same stopping criteria as detailed in Section 3.3 of the protocol.

The sponsor, in consultation with the investigator, may choose not to initiate any or all adaptive cohorts if deemed unnecessary.

The cohorts and study treatments for Part C are as follows:

Cohort	Day -1*	Single Dose (Day 1) or Multiple Doses (Days 1-14)
**11-15**	PTM capsule(s) QD or BID	≤ 50 mg total daily dose TLC-2716 or PTM, administered QD or BID, fasting or fed

BID = twice daily; PTM = placebo-to-match; QD = once daily.
* Dosing of PTM on Day -1 will only be performed in multiple-dose adaptive cohorts (as in Part B).

1.3. Study Blinding

This study will be conducted as a double-blind study using placebo capsules that are indistinguishable from TLC-2716 capsules in appearance and labeling. During the randomized

phase, the OrsoBio study team and site pharmacist(s) will be unblinded, while the investigational sites, including investigators and the subjects participating in the study will remain blinded. To mitigate the risks of inadvertently releasing the treatment information to the site or subjects, OrsoBio staff will only be provided with the unblinded information when there is a need to access such information for data analysis to support internal decision making. Should OrsoBio staff receive unblinded information unnecessarily, they will keep it confidential and will not communicate the information to the blinded site staff or subjects.

1.4. Randomization

1.4.1. Part A (SAD), Cohorts 1-5

- Subjects with confirmation of eligibility will be randomized at each cohort by the site pharmacist.
- Part A consists of 5 cohorts, each with 10 subjects, randomized 4:1 to TLC-2716 or matching placebo.

1.4.2. Part B (MAD), Cohorts 6-10

- Subjects with confirmation of eligibility will be randomized at each cohort by the site pharmacist.
- Part B consists of 5 cohorts, each with 10 subjects, randomized 4:1 to TLC-2716 or matching placebo.

1.4.3. Part C (Adaptive SAD and/or MAD), Cohorts 11-15

- Subjects with confirmation of eligibility will be randomized at each cohort by the site pharmacist.
- Part C consists of up to 5 cohorts, each with 10 subjects, randomized 4:1 to TLC-2716 or matching placebo.

1.5. Sample Size

The target sample size in each part is not based on statistical evidence, however, the following sample size is common considering similar Phase 1 studies and is appropriate for evaluating the safety and PK of TLC-2716. If screening is performed for confirmation of eligibility and the subject does not take part in this study, the subject will not be treated as a randomized subject unless otherwise specified in the protocol.

1.5.1. Part A (SAD), Cohort 1-5

Randomly assigned subjects will be secured to achieve 50 subjects in total, including 10 evaluable subjects per cohort.

1.5.2. Part B (MAD), Cohort 6-10

Randomly assigned subjects will be secured to achieve 50 subjects in total, including 10 evaluable subjects per cohort.

1.5.3. Part C (Adaptive SAD and/or MAD), Cohorts 11-15

Randomly assigned subjects will be secured to achieve up to 50 subjects in total, including 10 evaluable subjects per cohort.

1.6. Study Intervention

1.6.1. Part A (SAD), Cohorts 1-5

In Part A, the following cohorts will be performed:

Cohort	Day 1
1	0.5 mg TLC-2716 (1 x 0.5 mg) or PTM capsule SD, fed
2	2 mg TLC-2716[a] (1 x 2 mg) or PTM capsule SD, fed
3	6 mg TLC-2716[a] (3 x 2 mg) or PTM capsules SD, fed
4	12 mg TLC-2716[a] (2 x 5 mg; 1 x 2 mg) or PTM capsules SD, fed
5	20 mg TLC-2716[a] (4 x 5 mg) or PTM capsules SD, fed

PTM = placebo-to-match; SD = single dose.
[a] The planned doses may be modified based on the observed safety, tolerability, and any relevant and available PK and/or PD data. Any modified dose will be within 3-fold of a dose previously tested.

1.6.2. Part B (MAD), Cohorts 6-10

In Part B, up to 5 dose-escalation cohorts may be performed as outlined below. For Cohorts 7-10, dosing may be initiated after evaluation of cumulative safety, tolerability, and any relevant and available PK and/or PD data from all subjects enrolled in the previous cohort in Part B through Day 14.

Cohort	Day -1	Days 1-14
6	PTM capsule SD	0.5 mg TLC-2716 (1 x 0.5 mg) or PTM capsule, fed
7	PTM capsule SD	2 mg TLC-2716 [a] (1 x 2 mg) or PTM capsule, fed
8	PTM capsules SD	6 mg TLC-2716 [a] (3 x 2 mg) or PTM capsules, fed
9	PTM capsules SD	12 mg TLC-2716 [a] (2 x 5 mg; 1 x 2 mg) or PTM capsules, fed
10	PTM capsules SD	20 mg TLC-2716 [a] (4 x 5 mg) or PTM capsules, fed

PTM = placebo-to-match; SD = single dose.
[a] The planned doses may be modified based on the observed safety, tolerability, and any relevant and available PK and/or PD data. Any modified dose will be within 3-fold of a dose previously tested.

1.6.3. Part C (Adaptive SAD and/or MAD), Cohorts 11-15

In Part C, the following optional cohorts may be performed:

Cohort	Day -1*	Single Dose (Day 1) or Multiple Doses (Days 1-14)
**11-15**	PTM capsule(s) QD or BID	≤ 50 mg total daily dose TLC-2716 or PTM, administered QD or BID, fasting or fed

BID = twice daily; PTM = placebo-to-match; QD = once daily.
* Dosing of PTM on Day -1 will only be performed in multiple-dose adaptive cohorts (as in Part B).

2. ANALYSIS POPULATIONS

The analysis populations in this study are defined as follows.

2.1. All Randomized Analysis Set

All subjects randomly assigned to study intervention.

2.2. Safety Analysis Set

All subjects randomly assigned to study intervention and who took at least 1 dose of study intervention. Subjects will be analyzed according to the initial intervention they actually received.

2.3. Pharmacokinetic Analysis Set

All subjects randomly assigned to study intervention who took at least 1 dose of study drug and have sufficient plasma concentration data to reliably calculate PK parameters.

3. STATISTICAL CONSIDERATIONS FOR DATA ANALYSES AND HANDLINGS

3.1. Statistical Reporting

In principle, analyses for safety will be summarized by the actual treatment group and analyses will be performed by subjects dosed with placebo and subjects dosed with TLC-2716 by cohorts for Part A and Part B. Subjects dosed with placebo will be analyzed as 1 group in each part. To assess the effect of food, analyses may be performed by fasting state and after a meal, as applicable.

Unless otherwise stated, continuous variables will be summarized by the number of non-missing observations, arithmetic mean, standard deviation (SD), median, Q1, Q3, minimum, and maximum values as summary statistics; categorical variables will be summarized by the frequency count and the percentage of subjects in each category.

All analyses will be performed using SAS Version 9.4 (SAS Institute, Cary, NC, USA).

3.2. Confidence Interval

The Clopper-Pearson method will be used to calculate the confidence interval of the proportion, unless otherwise stated.

3.3. Analysis Visit Windows

Measurements will be allocated to an analysis visit date on case report forms (CRFs) collected based on the Schedule of Assessments in the protocol. The data reported at discontinuation will be treated as data at discharge.

3.3.1. Data Handling Conventions and Transformations

In general, age (in years) on the date of the first dose of study drug will be used for analyses and presentation in listings. If an enrolled subject was not dosed with any study drug, the randomization date will be used instead of the first dosing date of study drug. If only the birth year is collected on the CRF, "01 July" will be used for the unknown day of birth and month for the purpose of age calculation. If only birth year and month are collected, "01" will be used for the unknown day of birth.

Non-PK data that are continuous in nature but are less than the lower limit of quantitation (LOQ) or above the upper LOQ will be imputed as follows:

- A value that is 1 unit less than the lower LOQ will be used to calculate descriptive statistics if the datum is reported in the form of "< x" (where x is considered the lower LOQ). For example, if the values are reported as < 50 and < 5.0, values of 49 and 4.9, respectively, will be used to calculate summary statistics. An exception to this rule is any

value reported as < 1 or <0.1, etc. For values reported as < 1 or < 0.1, a value of 0.9 or 0.09, respectively, will be used to calculate summary statistics.

- A value that is 1 unit above the upper LOQ will be used to calculate descriptive statistics if the datum is reported in the form of "> x" (where x is considered the upper LOQ). Values with decimal points will follow the same logic as the bullet point above.
- The LOQ will be used to calculate descriptive statistics if the datum is reported in the form of "≤ x" or "≥ x" (where x is considered the lower or upper LOQ respectively).

If methods based on the assumption that the data are normally distributed are not adequate, analyses may be performed on transformed data or nonparametric analysis methods may be used, as appropriate.

Natural logarithmic transformation will be used for analyzing concentrations and PK parameters. Concentration values that are below the limit of quantitation (BLQ) will be presented as "BLQ" in the concentration data listing. Values that are BLQ will be treated as 0 at predose time points, and one-half the value of the LOQ at postdose time points for summary purposes.

The following conventions will be used for the presentation of summary and order statistics:

- If at least 1 subject has a concentration value of BLQ for the time point, the minimum value will be displayed as "BLQ."
- If more than 25% of the subjects have a concentration data value of BLQ for a given time point, the minimum and Q1 values will be displayed as "BLQ."
- If more than 50% of the subjects have a concentration data value of BLQ for a given time point, the minimum, Q1, and median values will be displayed as "BLQ."
- If more than 75% of the subjects have a concentration data value of BLQ for a given time point, the minimum, Q1, median, and Q3 values will be displayed as "BLQ."
- If all subjects have concentration data values of BLQ for a given time point, all order statistics (minimum, Q1, median, Q3, and maximum) and summary statistics will be displayed as "BLQ."

PK parameters that are BLQ will be imputed as one-half LOQ before log transformation or statistical model fitting.

3.4. Visit Windows

3.4.1. Definition of Predose and Postdose

The predose value associated with a treatment is defined as the last available value collected prior to the time of the first dose of the treatment (TLC-2716 or placebo) for the planned cohort

on Day -1, which may occur after placebo treatment of all subjects in MAD cohorts in Part B and C on Day -2.

Postdose value associated with a treatment is defined as any value collected after the first dose of that treatment and on or before the date of the last dose of that treatment plus 30 days, or before the time of the first dose of the following treatment, whichever comes earlier.

3.4.2. Analysis Visit Windows

The nominal visit as recorded on the CRF will be used when data are summarized by visit. Any data relating to unscheduled visits will not be assigned to a particular visit or time point and in general will not be included in summaries. However, the following exceptions will be made:

- An unscheduled visit prior to the first dose of study drug may be included in the calculation of predose value, if applicable.
- For subjects who discontinue from the study, early termination (ET) data will be summarized as a separate visit, labeled as “Early Termination”.
- Data collected on a follow-up visit will be summarized as a separate visit and labeled “Follow-up”.

3.4.3. Selection of Data in the Event of Multiple Records on the Same Day

If multiple valid, nonmissing numeric observations exist on a day, records will be chosen based on the following rules if a single value is needed:

- For predose, the last available record on or prior to the date and time of the first dose of study drug will be selected. If there are multiple records with the same time or no time recorded on the same day, average (arithmetic or geometric mean, as appropriate) will be used for the predose value. Of note, predose collections which occur in MAD cohorts on dosing days after Day 1 will not be included in the predose value averaging.
- For postdose values:
    - The record closest to the nominal day for that visit will be selected.
    - If there are 2 records that are equidistant from the nominal day, the later record will be selected.
    - If there is more than 1 record on the selected day, the average will be taken, unless otherwise specified.

If multiple, valid, nonmissing categorical observations exist on a day, records will be chosen based on the following rules if a single value is needed:

- For predose, the last available record on or prior to the date and time of the first dose of study drug will be selected. If there are multiple records with the same time or no time recorded on the same day, the value with the lowest severity will be selected (e.g., normal will be selected over abnormal for safety ECG findings).
- For postdose values, follow the same rules described above for postbaseline numeric observations, except that if there are multiple records on the same day, the most conservative value will be selected (e.g., abnormal will be selected over normal for safety ECG findings).

3.5. Missing Data

Missing data can have an impact on interpretation of trial data. As this study is of short duration, it is anticipated that missing data will be minimal. In general, values for missing data will not be imputed. However, a missing pre-treatment laboratory result would be treated as normal (i.e., no toxicity grade) for the laboratory abnormality summary. All statistical analyses will be based on observed case unless otherwise stated.

3.6. Outliers

Outliers of non-PK data will be identified during the data management and data analysis process, but no sensitivity analyses will be conducted. All data will be included in the data analysis.

3.7. Definitions

3.7.1. Study Day

Study Day 1 refers to the date of initial administration of the study intervention (TLC-2716 or placebo). Other study days are defined relative to Study Day 1, and previous days to Study Day 1 are expressed as Day -1 or Day -2 (there is no Study Day 0). Note, in the event an entire cohort is administered placebo on a day prior to Day 1 (e.g., Day -1) for the purposes of supporting additional exploratory biomarker or PD assessments, Day 1 will remain the day upon which randomized subjects are considered to have received TLC-2716 or placebo.

3.7.2. Baseline

Baseline is defined as the last value obtained before the administration of study intervention (TLC-2716 or placebo), unless otherwise stated. For laboratory data, baseline will be Day -1 for all cohorts in Parts A, B, and C. For ECG parameters, baseline will be Day -1 for SAD Cohorts in Parts A and C, and Day -2 for MAD cohorts in Parts B and C.

4. DEMOGRAPHIC AND OTHER BASELINE CHARACTERISTICS

4.1. Subject Disposition

The following analyses will be performed for all randomized subjects.

The number and proportion of subjects who completed the study, and the number of subjects who discontinued the study will be determined. In addition, the reason for discontinuation from the study will be summarized by cohorts and treatment interventions.

The number and proportion of subjects in each analysis population will be summarized as well as the reasons for exclusion from each analysis population.

The number and proportion of subjects who met important protocol deviation categories will be summarized with the reason for deviation by cohorts and treatment interventions.

4.2. Demographic and Baseline Characteristics

Subject demographic variables (i.e., age, sex, race, and ethnicity) and baseline characteristics (body weight [in kg], height [in cm], body mass index [BMI; in kg/m^2]) will be summarized for each cohort, including pooled placebo subjects, in Parts A, B, and C. The summary of demographic data will be provided for the Safety Analysis Set.

A by-subject demographic listing, including the informed consent date, will be provided by subject ID number in ascending order.

4.3. Medical History

Medical history data will be collected at Screening and listed only. A by-subject listing of general medical history will be provided by subject ID number and onset date in chronological order. The listing will include relevant medical condition or procedure reported term, onset date, ongoing status, and resolution date, if applicable.

5. STUDY CONDUCT

5.1. Prior and Concomitant Medications/Therapies

Prior medications/therapies are defined as medications/therapies which have been taken prior to administration of study intervention. Concomitant medications/therapies are defined as medications/therapies taken at or after administration of study intervention. Prior and concomitant medications will be coded using the latest version of the WHO Drug Global Dictionary.

For the Safety Analysis Set, a by-subject listing of prior and concomitant medications/therapies will be provided by subject ID number and administration date in chronological order.

5.2. Treatment Exposure and Compliance

A subject's extent of exposure to study drug data will be generated from the study drug administration page in the eCRF. Exposure data will be listed.

Total days of administration is defined as total number of days of study drug administration (TLC-2716 or placebo).

Treatment compliance rate will be summarized descriptively by cohorts and treatment interventions for the Safety Population. Treatment compliance rate is defined as: [100% * [(total days of administration) * (total days of planned administration)]].

6. EFFICACY ANALYSES

All efficacy analyses are exploratory. In MAD cohorts in Part B and C, exploratory analyses will be performed to describe absolute and relative (%) changes from baseline in lipid parameters, including fasting triglycerides, total cholesterol, low-density lipoprotein cholesterol (LDL-C), and high-density lipoprotein cholesterol (HDL-C). Exploratory subgroup analyses may also be conducted to describe changes in fasting triglycerides in subjects with baseline triglycerides ≥ 100 mg/dL versus < 100 mg/dL. Similarly, exploratory subgroup analyses may be performed to describe changes from baseline in fasting LDL-C in subjects with baseline LDL-C ≥ 100 mg/dL versus < 100 mg/dL.

7. SAFETY

7.1. Adverse Events and Deaths

7.1.1. Adverse Event Dictionary

Clinical and laboratory adverse events (AEs) will be coded using the current version of the Medical Dictionary for Regulatory Activities (MedDRA). System organ class (SOC), high-level group term (HLGT), high-level term (HLT), preferred term (PT), and lower-level term (LLT) will be provided in the AE dataset.

7.1.2. Adverse Event Severity

Adverse events are graded by the investigator as Grade 1, 2, 3, or 4 according to toxicity criteria specified in the protocol. The severity grade of events for which the investigator did not record severity will be categorized as “missing” for tabular summaries and data listings. The missing category will be presented last in the summary presentation.

7.1.3. Relationship of Adverse Events to Study Drug

Study drug related AEs are those for which the investigator selected “Related” on the AE case report form (CRF) in response to the question regarding relatedness of AE to study drug. Relatedness will always default to the investigator’s choice, not that of the medical monitor, or designee. Events for which the investigator did not record relationship to study drug will be considered related to study drug for summary purposes. However, by-subject data listings will show the relationship as missing as documented on the CRF.

7.1.4. Relationship of Adverse Events to Study Procedure

Study procedure related AEs are those for which the investigator selected “Yes” on the AE CRF to the question regarding relatedness to protocol mandated procedures. Relatedness will always default to the investigator’s choice, not that of the medical monitor. Events for which the investigator did not record relationships to study procedure will be considered related to study procedure for summary purposes. However, by-subject data listings will show the relationship as missing from that captured on the CRF.

7.1.5. Serious Adverse Events

Serious adverse events (SAEs) will be identified and captured as SAEs if the AEs met the definition of SAEs that were specified in the study protocol.

7.1.6. Treatment-Emergent Adverse Events

7.1.6.1. Definition of Treatment Emergent

Treatment-emergent adverse events (TEAEs) are defined as 1 or both of the following:

- Any AEs with an onset date on or after the study drug start date and no later than 30 days after permanent discontinuation of study drug. If the AE onset date is the same as the date of study drug start date then the AE onset time must be on or after the study drug start time. If the AE onset time is missing when the start dates are the same the AE will be considered treatment emergent.
- Any AEs leading to premature discontinuation of study drug.

7.1.6.2. Incomplete Dates

If the onset date of the AE is incomplete and the AE stop date is not prior to the first dosing date of study drug, then the month and year (or year alone if month is not recorded) of onset determine whether an AE is treatment emergent.

An AE with completely missing onset and stop dates, or with the onset date missing and a stop date later than the date of the first dose of study drug, will be considered to be treatment emergent. In addition, an AE with the onset date missing and incomplete stop date with the same or later month and year (or year alone if month is not recorded) as the first dosing date of study drug will be considered treatment emergent.

7.1.6.3. Summaries of Adverse Events and Deaths

Treatment-emergent AEs will be summarized based on the Safety Analysis Set.

The number and percentage of subjects who experienced at least 1 TEAE will be provided and summarized by SOC, PT, and treatment group as follows:

- All TEAEs
- TEAEs of Grade 3 or higher
- TEAEs of Grade 2 or higher
- All TEAEs by severity grade
- All TE treatment-related AEs
- TE treatment-related AEs of Grade 3 or higher (by maximum severity)
- TE treatment-related AEs of Grade 2 or higher
- All TE treatment-related AEs by severity grade
- All TEAEs related to study procedures
- All TESAEs

- All TE treatment-related SAEs
- All TEAEs leading to premature discontinuation of study drug
- All TEAEs leading to premature discontinuation of study

A brief, high-level summary of AEs described above will be provided by treatment group and by the number and percentage of subjects who experienced the above AEs. All deaths observed in the study will also be included in this summary.

Multiple events will be counted only once per subject per treatment in each summary. Adverse events will be summarized and listed first in alphabetic order of SOC and then by PT in descending order of total frequency within each SOC. For summaries by severity grade, the most severe grade will be used for those AEs that occurred more than once in an individual subject per treatment during the study.

In addition, data listings will be provided for the following:

- All AEs, indicating whether the event is treatment emergent
- All AEs of Grade 3 or higher
- All AEs of Grade 2 or higher
- SAEs
- Deaths
- AEs leading to premature discontinuation of study drug
- AEs leading to discontinuation of study

7.2. Laboratory Evaluations

Laboratory data collected during the study will be analyzed and summarized using both quantitative and qualitative methods. Summaries of laboratory data will be provided for the Safety Analysis Set and will include data collected up to the last dose of study drug plus 30 days for subjects who have permanently discontinued study drug. The analysis will be based on values reported in conventional units. When values are BLQ, they will be listed as such, and the imputed value will be used for the purpose of calculating summary statistics. Hemolyzed test results will not be included in the analysis, but they will be listed in by-subject laboratory listings.

A by-subject listing for laboratory test results will be provided by subject ID number and visit in chronological order for hematology, serum chemistry, lipids, coagulation, and urinalysis

separately. As appropriate, abnormal values will be flagged in data listings per Common Terminology Criteria for Adverse Events (CTCAE) v5.0 criteria.

No formal statistical testing is planned.

7.2.1. Summaries of Numeric Laboratory Results

Descriptive statistics will be provided by treatment group for each laboratory test specified in the study protocol as follows:

- Predose values
- Values at each postdose visit
- Change from predose at each postdose visit

Predose and postdose values will be defined as described in Section 3.7.1. Change from predose to a postdose visit will be defined as the visit value minus the predose value. Laboratory test results collected at unscheduled visits will be included for the predose and postdose maximum and minimum value selection. The mean, median, Q1, Q3, minimum, and maximum values will be displayed to the reported number of digits; SD values will be displayed to the reported number of digits plus 1.

In the case of multiple values in an analysis window, data will be selected for analysis as described in Section 3.4.

7.2.2. Graded Laboratory Values

As appropriate, CTCAE v5.0 will be used to assign toxicity grades to laboratory results for analysis. Treatment-Emergent Laboratory Abnormalities

Treatment-emergent laboratory abnormalities are defined as values that increase at least 1 toxicity grade from predose assessment associated with a treatment group and occurring after the first dose of that treatment group and on or before the date of the last dose of that treatment group plus 30 days or before the first dose of the following treatment group (if applicable), whichever occurs earlier. If the relevant predose laboratory value is missing, any abnormality of at least Grade 1 observed within the time frame specified above will be considered treatment emergent.

7.2.2.1. Summaries of Laboratory Abnormalities

Laboratory data that are categorical will be summarized using the number and percentage of subjects in the study with the given response at predose and each scheduled postdose visit.

The following summaries (number and percentage of subjects) for treatment-emergent laboratory abnormalities will be provided by lab test and treatment group; subjects will be categorized

according to the most severe postdose abnormality grade for a given lab test within a treatment group:

- Graded laboratory abnormalities
- Grade 3 or 4 laboratory abnormalities

For all summaries of laboratory abnormalities, the denominator is the number of subjects with nonmissing postdose values up to 30 days after last dosing date.

A by-subject listing of treatment-emergent Grade 3 or 4 laboratory abnormalities will be provided by subject ID number and visit in chronological order. This listing will include all test results that were collected throughout the study for the lab test of interest, with all applicable severity grades displayed.

7.2.3. Vital Signs

Descriptive statistics will be provided by treatment group, including predose assessment on Day -1 (Baseline) and change from baseline at each postdose visit. Change from predose to postdose visit will be defined as the postdose value minus the predose value. Vital signs to be described include respiratory rate, heart rate, systolic blood pressure, diastolic blood pressure, and body temperature. In event of multiple values in an analysis window, data will be selected for analysis as described in Section 3.4. No formal statistical testing is planned. A by-subject listing of vital signs will be provided by subject ID number and visit in chronological order.

7.2.4. 12-lead Electrocardiograms

ECG findings (Normal, Abnormal-not clinically significant, Abnormal-clinically significant) will be summarized by cohorts and study interventions. ECG parameters include ventricular rate, PR interval, QRS interval, QT interval, RR interval, and Summary statistics of measurement values and each change from baseline in ECG parameters will be calculated by cohorts and study interventions for each time point. The time points are shown in the tables under Section 13.

8. PHARMACOKINETIC ANALYSIS

8.1.1. Data Handling

All PK analyses will use the PK Analysis Set. Plasma concentrations and plasma PK parameters will be listed and summarized for TLC-2716 and its metabolites (TLC-6665, TLC-7255), as applicable using descriptive statistics by treatment and PK Day (as applicable) for each cohort. Plasma concentration versus time curves will also be presented.

Plasma concentrations that are below the limit of quantification (BLQ) will be treated as zero for calculation of descriptive statistics. When all concentrations are BLQ for a timepoint, the mean BLQ concentrations will be presented as BLQ, and the SD and CV will be reported as not applicable.

For PK parameter calculation, BLQ values prior to a quantifiable concentration will be treated as zero. BLQ values between 2 quantifiable concentrations, or in the terminal phase will be set as missing. If consecutive BLQ concentrations in the terminal phase are followed by quantifiable concentrations, those concentrations after consecutive BLQ concentrations will be treated as missing.

No missing data imputation will be performed.

Missing concentrations will be treated as missing from the descriptive statistics and PK parameter calculations.

8.1.2. Plasma Pharmacokinetic Concentrations

Part A – SAD:

Day 1: Blood samples for PK analysis of concentrations of TLC-2716 and its metabolites in plasma will be collected at the following timepoints: predose (≤ 10 minutes prior to dosing) and 0.25, 0.5, 1, 1.5, 2, 2.5, 3, 3.5, 4, 6, 12, 24, 48, and 72 hours postdose.

Part B – MAD and Part C – Adaptive SAD and/or MAD:

Day 1: Blood samples for PK analysis of concentrations of TLC-2716 and its metabolites in plasma will be collected at the following timepoints: predose (≤ 10 minutes prior to dosing) and 0.25, 0.5, 1, 1.5, 2, 2.5, 3, 3.5, 4, 6, 12, and 24 hours postdose.

Days 3 and 7: Predose (≤ 10 minutes prior to dosing) and 2 and 4 hours postdose.

Day 14: Blood samples for PK analysis of concentrations of TLC-2716 and its metabolites in plasma will be collected at the following timepoints: predose (≤ 10 minutes prior to dosing) and 0.25, 0.5, 1, 1.5, 2, 2.5, 3, 3.5, 4, 6, 12, 24, 48, and 72 hours postdose.

Individual plasma concentrations of TLC-2716 and its metabolites will be presented in data listings and summarized separately using descriptive statistics (N [number of subjects], n [non-missing values within the population], arithmetic mean, SD, CV%, median, minimum, and maximum) by study part, treatment, cohort, day, as applicable, and nominal time point. Mean (± SD) plasma concentrations of TLC-2716 and its metabolites will be plotted by study part, dose, cohort, day, as applicable, and nominal time on both linear and semi-logarithmic scales.

8.1.3. Plasma Pharmacokinetic Parameters

The plasma concentration-time data for TLC-2716 and its metabolites will be analyzed by noncompartmental analysis using Phoenix® WinNonLin® Version 8.3.4 or higher (Certara USA, Inc., Princeton, NJ). The following single- and multiple-dose PK parameters will be calculated for TLC-2716 and its metabolites as applicable based on actual times, where data permit:

PK Parameter	Definition
C_{max}	Maximum observed plasma concentration
t_{max}	Time of maximum observed concentration
t_{last}	Time of last measurable concentration
AUC_{last}	AUC from time 0 to the last quantifiable concentration, calculated using the linear trapezoidal rule
AUC_{inf}	AUC from time 0 extrapolated to infinity, calculated using the linear trapezoidal method
AUC_{tau}	Area under the concentration-time curve for one dosing interval (tau). (Day 1 and Day 14 for MAD cohorts)
$t_{1/2}$	Apparent terminal elimination half-life, calculated as: ln (2) / K_{el}
CL/F	Apparent total body clearance, calculated as: Dose / AUC_{inf}
V_z/F	Volume of distribution during the terminal phase, calculated as: Dose / [K_{el} * AUC_{inf}]
AR_{Cmax}	Accumulation ratio based on C_{max}, calculated as: C_{max} (Day 14) / C_{max} (Day 1)
AR_{AUC}	Accumulation ratio based on AUC_{tau}, calculated as: AUC_{tau} (Day 14) / AUC_{tau} (Day 1)

In addition to the above PK parameters, which will be listed and summarized, the following parameters will also be listed to document the selection of data points used to estimate $t_{1/2}$ using non-compartmental procedures:

PK Parameter	Definition
K_{el}	Elimination rate constant
Number points	Number of data points used to estimate K_{el}; a minimum of 3 data points must be used, and C_{max} must not be included.
K_{el} lower	Lower bound used for the estimation of K_{el}
K_{el} upper	Upper bound used for the estimation of K_{el}
Span	Number of elapsed half-lives over which K_{el} is estimated, calculated as (K_{el} upper – K_{el} lower) / $t_{½}$
Rsq	r^2, the coefficient of determination (goodness of fit statistic)
$\%AUC_{extrap}$	Percentage of AUC_{inf} due to extrapolation

Plasma PK parameters for TLC-2716 and its metabolites will be presented in data listings and summarized separately using descriptive statistics (N, n, arithmetic mean, SD, CV%, geometric mean, geometric CV%, median, minimum, and maximum) by study part, treatment, cohort, and

day, as applicable. t_{max} , $t_{1/2}$, and t_{last} will be summarized using the descriptive statistics median, minimum, and maximum only.

8.1.4. Pharmacokinetic Statistical Analyses

8.1.4.1. Dose Proportionality

Dose-proportionality of plasma TLC-2716 and its metabolites, PK parameters, C_{max}, AUC_t, AUC_{tau}, and AUC_{inf}, as applicable for Part A, Part B, and Part C (as applicable) over the dose range tested will be investigated. A power regression model will be fitted to describe the relationship between Y (C_{max}, AUC_{tau}, AUC_{last}, and AUC_{inf}) and X (dose) under fasting conditions. The model is defined as:

$$ln(Y) = \beta_0 + \beta_1 ln(X),\text{ where Y is PK parameter and X is dose}$$

The null hypothesis that will be tested is that the AUCs and C_{max} values are dose-proportional, or slope β_1=1 is within 90% CI of the slope.

An alternative evaluation of dose proportionality may be conducted using analysis of variance (ANOVA) on dose normalized, natural log-transformed AUC and C_{max} of TLC-2716 and its metabolites. The model will include dose as a fixed effect. The two-sided 90% CI for the ratio of dose comparisons will be constructed.

8.1.5. Evaluation of Food Effect

For statistical evaluation of food-effect, PK parameters (AUC_{last}, AUC_{inf}, and C_{max}) of TLC-2716 under fasted conditions versus fed conditions for selected cohort(s) will be compared by ANOVA using a linear mixed effect model with fed/fasted status as a fixed effect and participant as a random effect, unless otherwise stated. The ratio of geometric means and associated 90% CI will also be calculated for AUC_{last}, AUC_{inf} and C_{max} between the fed and fasted conditions.

8.1.6. Evaluation of Accumulation

Comparisons between Day 1 (AUC_{tau}, and C_{max}) and Day 14 PK parameters (AUC_{tau}, and C_{max}) will be performed by ANOVA using the MIXED Procedure for each treatment cohort, with the natural log-transformed values of PK parameters as the dependent variable, study day as the fixed effect, and subject as a random effect, unless otherwise stated. The ratio (Day 14/Day 1) of the geometric least square mean and its corresponding 90% CI will be calculated.

9. PHARMACODYNAMIC ANALYSIS

Exploratory descriptive analysis of PD markers collected in this study, including absolute and relative (percent) changes from baseline, may be evaluated.

10. INTERIM ANALYSES

No interim analysis is planned.

11. PROGRAMMING CONVENTIONS

11.1. Formatting and Programming Rule

Unless otherwise stated, the following conventions should be applied in constructing the analysis tables, figures and listings:

- Every summary table and figure should clearly specify the analysis population being summarized.
- Rounding for all variables will occur only at the last step, immediately prior to presentation in tables, figures and listings. No intermediate rounding will be performed on derived variables. The standard practice of rounding numbers ending in 0-4 down and numbers ending in 5-9 up will be employed.
- The presentation of numerical values will adhere to the following guidelines.
    - Raw measurements will be reported to the number of significant digits as captured electronically or on the CRFs.
    - Means, standard deviations, medians, minimums, maximums, Q1, and Q3 values will be reported to one decimal place beyond the number of decimal places with which the original endpoint is presented.
    - Calculated percentages will be reported to one decimal place. All means presented are arithmetic unless otherwise stated.
- All means presented are arithmetic unless otherwise stated.

12. REFERENCES

Not applicable.

13. APPENDICES

Appendix 13-1. Study 2716-CL-101 Schedule of Assessments

Appendix 13-1. Study 2716-CL-101 Schedule of Assessments

Table 1. Schedule of Assessments: SAD Cohorts in Parts A and C

Study Procedure	Screening[a]	Day -1 (Admission)	Day 1	Day 2	Day 3	Day 4[b]	Follow-up[c]: Day 15 (± 2)	ET[d]
Written informed consent	X[r]							
Medical history	X							
Height	X							
Weight & BMI	X	X				X	X	
Vital signs[e]	X	X	X	X	X	X	X	X
Complete physical exam	X	X				X	X	X
Symptom-driven physical exam[f]			X	X	X			
HIV-1, HBV, and HCV serology	X							
Hematology[g]	X	X	X[k]	X	X	X	X	X
Serum chemistry[h]	X	X	X[k]	X	X	X	X	X
eGFR	X							
Coagulation[i]	X	X	X[k]	X	X	X	X	X
Lipids[j]	X	X	X[k]	X	X	X	X	X
Urinalysis	X	X	X[k]	X	X	X	X	X
Serum pregnancy test[l]	X					X	X	X
Urine pregnancy test[l]		X						
FSH[m]	X							
Urine and alcohol drug screen	X	X						
12-Lead ECG	X	X	X[k]	X		X	X	X
Randomization	X	X						

Study Procedure	Screening[a]	Day -1 (Admission)	Day 1	Day 2	Day 3	Day 4[b]	Follow-up[c]: Day 15 (± 2)	ET[d]
Study drug or PTM administration			X					
Plasma PK[n]			X	X	X	X		
Plasma PD[o]			X					
Biomarkers: Exploratory[o]			X					
Optional genomic testing[p]		X						
Clinic confinement[q]		X	X	X	X	X		
Review AEs and concomitant medications[r]	X	X	X	X	X	X	X	X

eGFR = estimated glomerular filtration rate; ET = early termination

a Prospective subjects should be screened no more than 28 days prior to administration of the first dose of study drugs.
b Subjects will be discharged from the center on Day 4, following all morning assessments.
c 14 (± 2) days after the last dose of the study drug, all subjects will return for an in-clinic follow-up visit.
d Assessments will be performed within 72 hours of early termination from the study.
e Vital signs include blood pressure, pulse, respiration rate, and body temperature.
f Symptom driven physical exams will be performed during confinement as needed, based on reported signs and symptoms, on days in which complete physical examination is not required.
g Hematology: CBC with differential. Collected in fasting state prior to morning meal.
h Serum Chemistry: alkaline phosphatase, AST, ALT, GGT, total bilirubin, direct and indirect bilirubin, total protein, albumin, bicarbonate, BUN, calcium, chloride, creatinine, glucose, phosphorous, magnesium, potassium, sodium, uric acid, and amylase (reflex lipase testing is performed in subjects with total amylase > 1.5 X ULN). Collected in fasting state prior to morning meal.
i Coagulation: INR, PTT. Collected in fasting state prior to morning meal.
j Lipids: total cholesterol, HDL, LDL, TG. Collected in fasting state prior to morning meal.
k To be collected in fasting state prior to morning meal and 12 hours post-dose, with exception of 12-lead ECG which is only required 2 hours post-dose.
l Females of child-bearing potential only (see protocol Appendix 3 for definition).
m As necessary only for evaluation of post-menopausal state in females of any age with amenorrhea > 12 months at time of Screening (see protocol Appendix 3).
n Collected pre-dose (≤ 10 minutes prior to dosing), 0.25, 0.5, 1, 1.5, 2, 2.5, 3, 3.5, 4, 6, 12, 24, 48, and 72 hours post-dose.
o Collected pre-dose (≤ 10 minutes prior to dosing), and 4 hours post-dose.
p The optional genomic sample should be collected on Day -1, but may be collected at any time during the study, if necessary.
q Subjects to be educated on study requirements and restrictions.
r From the time of obtaining informed consent through the first administration of the study drug, record all SAEs and any non-serious AEs related to protocol required procedures on the AE eCRF. All other untoward medical occurrences observed during the Screening period, including exacerbation or changes in medical history should be documented on the medical history eCRF. See protocol Section 7 Adverse Events and Toxicity Management for additional details.

Table 2. Schedule of Assessments: MAD Cohorts in Parts B and C

Study Procedure	Screening[a]	Day -2 (Admi-ssion)	Day -1	Day 1	Day 2	Day 3	Day 4	Day 5	Day 6	Day 7	Days 8-9	Day 10	Days 11-13	Day 14	Days 15-16	Day 17[b]	Follow-up[c]: Day 28 (± 2)	ET[d]
Written informed consent	X[v]																	
Medical history	X																	
Height	X																	
Weight & BMI	X	X								X						X	X	
Vital signs[e]	X	X	X	X	X	X		X		X		X		X		X	X	X
Complete physical exam	X	X														X	X	X
Symptom-driven physical exam[f]			X	X	X	X	X	X	X	X	X	X	X	X	X			
HIV-1, HBV, & HCV serology	X																	
Hematology[g]	X		X	X[k]	X	X		X		X		X		X		X	X	X
Serum chemistry[h]	X		X	X[k]	X	X		X		X		X		X		X	X	X
eGFR	X																	
Coagulation[i]	X		X	X[k]	X	X		X		X		X		X		X	X	X
Lipids[j]	X		X	X[k]	X	X		X		X		X		X		X	X	X
Urinalysis	X		X	X[k]	X	X		X		X		X		X		X	X	X
Serum pregnancy test[l]	X															X	X	X
Urine pregnancy test[l]		X																
FSH[m]	X																	
Urine & alcohol drug screen	X	X																

Study Procedure	Screening[a]	Day -2 (Admission)	Day -1	Day 1	Day 2	Day 3	Day 4	Day 5	Day 6	Day 7	Days 8-9	Day 10	Days 11-13	Day 14	Days 15-16	Day 17[b]	Follow-up[c]: Day 28 (± 2)	ET[d]
12-Lead ECG	X	X		X[k]		X				X				X		X	X	X
Randomization			X															
Study drug or PTM administration[n]			X[n]	X[n]	X	X	X	X	X	X	X	X	X	X				
Plasma PK[o]				X		X				X				X	X	X		
Plasma PD[p]			X	X						X				X				
Biomarker: whole blood collection[q]				X										X				
Plasma NMR LipoProfile[r]				X										X				
Biomarker: Stool collection[s]			X											X				
Biomarkers: Exploratory[p]			X	X						X				X				
Optional genomic testing[t]			X															
Clinic confinement[u]		X	X	X	X	X	X	X	X	X	X	X	X	X	X	X		
Review AEs & concomitant medications[v]	X	X	X	X	X	X	X	X	X	X	X	X	X	X	X	X	X	X

eGFR = estimated glomerular filtration rate; ET = early termination
a Prospective subjects should be screened no more than 28 days prior to administration of the first dose of study drugs.
b Subjects will be discharged from the center on Day 17, following all morning assessments.
c 14 (± 2) days after the last dose of the study drug, all subjects will return for an in-clinic follow-up visit.
d Assessments will be performed within 72 hours of early termination from the study.
e Vital signs include blood pressure, pulse, respiration rate, and body temperature.
f Symptom driven physical exams will be performed during confinement as needed, based on reported signs and symptoms.
g Hematology: CBC with differential. Collected in fasting state prior to morning meal, if applicable.

h Serum Chemistry: alkaline phosphatase, AST, ALT, GGT, total bilirubin, direct and indirect bilirubin, total protein, albumin, bicarbonate, BUN, calcium, chloride, creatinine, glucose, phosphorous, magnesium, potassium, sodium, uric acid, and amylase (reflex lipase testing is performed in subjects with total amylase > 1.5 X ULN). Collected in fasting state prior to morning meal, if applicable.

i Coagulation: INR, PTT. Collected in fasting state prior to morning meal, if applicable.

j Lipids: total cholesterol, HDL, LDL, TG. Collected in fasting state prior to morning meal, if applicable.

k On Day 1, to be collected 12 hours post-dose only, with exception of 12-lead ECG which should be 2 hours post-dose.

l Females of child-bearing potential only (see protocol Appendix 3 for definition).

m As necessary only for evaluation of post-menopausal state in females of any age with amenorrhea > 12 months at time of Screening (see protocol Appendix 3).

n For MAD cohorts, all subjects will receive PTM on Day -1, and study drug starting Day 1.

o PK sampling will occur relative to the morning dosing of TLC-2716 at the following time points for each cohort:

- Day 1: Pre-dose (≤ 10 minutes prior to dosing), 0.25, 0.5, 1, 1.5, 2, 2.5, 3, 3.5, 4, 6, and 12 hours post-dose
- Days 3, and 7: Pre-dose (≤ 10 minutes prior to dosing), 2, and 4 hours post-dose
- Day 14: Pre-dose (≤ 10 minutes prior to dosing), 0.25, 0.5, 1, 1.5, 2, 2.5, 3, 3.5, 4, 6, 12, 24, 48, and 72 hours post-dose

For any cohorts in Part C with BID administration, see Protocol Section 6.5 for details on the timing of PK collections.

p Collected relative to the morning dosing of TLC-2716 or PTM to measure PD biomarkers for TLC-2716 at the following time points:

- Day -1: (all ± 5 minutes of Day 1 time points), 0, 2, 4, 6, and 12 hours
- Day 1, 7, and 14: Pre-dose (≤ 10 minutes prior to dosing), and 2, 4, 6, and 12 hours post-dose

All post-dose timings are relative to morning dose for once-daily dosing or BID dosing.

q Collected pre-dose (≤ 10 minutes prior to dosing), and 4 hours post-dose.

r Collected pre-dose (≤ 10 minutes prior to dosing), 2, 4, 6, and 12 hours post-dose.

s All stools will be collected over approximately 24-hour intervals at the following time points:

- Day -1: Day -2 Admission through Day 1 (0, pre-dose)
- Days 14: 0 (pre-dose) through to 24 hours post-dose

t The optional genomic sample should be collected on Day -1, but may be collected at any time during the study, if necessary.

u Subjects to be educated on study requirements and restrictions.

v From the time of obtaining informed consent through the first administration of the study drug, record all SAEs and any non-serious AEs related to protocol required procedures on the AE eCRF. All other untoward medical occurrences observed during the Screening period, including exacerbation or changes in medical history should be documented on the medical history eCRF. See protocol Section 7 Adverse Events and Toxicity Management for additional details

Section/topic	No	CONSORT 2025 checklist item description	Reported on page no.
**Title and abstract**			
Title and structured abstract	1a	Identification as a randomised trial	2, 7-9
	1b	Structured summary of the trial design, methods, results, and conclusions	7-9, 30-32
**Open science**			
Trial registration	2	Name of trial registry, identifying number (with URL) and date of registration	7-9
Protocol and statistical analysis plan	3	Where the trial protocol and statistical analysis plan can be accessed	32
Data sharing	4	Where and how the individual de-identified participant data (including data dictionary), statistical code and any other materials can be accessed	32
Funding and conflicts of interest	5a	Sources of funding and other support (eg, supply of drugs), and role of funders in the design, conduct, analysis and reporting of the trial	13
	5b	Financial and other conflicts of interest of the manuscript authors	13
**Introduction**			
Background and rationale	6	Scientific background and rationale	3-7
Objectives	7	Specific objectives related to benefits and harms	7-9
**Methods**			
Patient and public involvement	8	Details of patient or public involvement in the design, conduct and reporting of the trial	30-32
Trial design	9	Description of trial design including type of trial (eg, parallel group, crossover), allocation ratio, and framework (eg, superiority, equivalence, non-inferiority, exploratory)	30-32
Changes to trial protocol	10	Important changes to the trial after it commenced including any outcomes or analyses that were not prespecified, with reason	30-32
Trial setting	11	Settings (eg, community, hospital) and locations (eg, countries, sites) where the trial was conducted	30-32
Eligibility criteria	12a	Eligibility criteria for participants	30-32
	12b	If applicable, eligibility criteria for sites and for individuals delivering the interventions (eg, surgeons, physiotherapists)	
Intervention and comparator	13	Intervention and comparator with sufficient details to allow replication. If relevant, where additional materials describing the intervention and comparator (eg, intervention manual) can be accessed	30-32
Outcomes	14	Prespecified primary and secondary outcomes, including the specific measurement variable (eg, systolic blood pressure), analysis metric (eg, change from baseline, final value, time to event), method of aggregation (eg, median, proportion), and time point for each outcome	30-32
Harms	15	How harms were defined and assessed (eg, systematically, non-systematically)	30-32
Sample size	16a	How sample size was determined, including all assumptions supporting the sample size calculation	30-32
	16b	Explanation of any interim analyses and stopping guidelines	30-32
Randomisation:			
Sequence generation	17a	Who generated the random allocation sequence and the method used	30-32
	17b	Type of randomisation and details of any restriction (eg, stratification, blocking and block size)	30-32

			Reported on page no.
Allocation concealment mechanism	18	Mechanism used to implement the random allocation sequence (eg, central computer/telephone; sequentially numbered, opaque, sealed containers), describing any steps to conceal the sequence until interventions were assigned	30-32
Implementation	19	Whether the personnel who enrolled and those who assigned participants to the interventions had access to the random allocation sequence	30-32
Blinding	20a	Who was blinded after assignment to interventions (eg, participants, care providers, outcome assessors, data analysts)	30-32
	20b	If blinded, how blinding was achieved and description of the similarity of interventions	30-32
Statistical methods	21a	Statistical methods used to compare groups for primary and secondary outcomes, including harms	30-32
	21b	Definition of who is included in each analysis (eg, all randomised participants), and in which group	30-32
	21c	How missing data were handled in the analysis	30-32
	21d	Methods for any additional analyses (eg, subgroup and sensitivity analyses), distinguishing prespecified from post hoc	30-32
**Results**			
Participant flow, including flow diagram	22a	For each group, the numbers of participants who were randomly assigned, received intended intervention, and were analysed for the primary outcome	7-9
	22b	For each group, losses and exclusions after randomisation, together with reasons	30-32
Recruitment	23a	Dates defining the periods of recruitment and follow-up for outcomes of benefits and harms	7-9
	23b	If relevant, why the trial ended or was stopped	
Intervention and comparator delivery	24a	Intervention and comparator as they were actually administered (eg, where appropriate, who delivered the intervention/comparator, how participants adhered, whether they were delivered as intended (fidelity))	30-32
	24b	Concomitant care received during the trial for each group	30-32
Baseline data	25	A table showing baseline demographic and clinical characteristics for each group	15, 19
Numbers analysed, outcomes and estimation	26	For each primary and secondary outcome, by group:<br>● the number of participants included in the analysis<br>● the number of participants with available data at the outcome time point<br>● result for each group, and the estimated effect size and its precision (such as 95% confidence interval)<br>● for binary outcomes, presentation of both absolute and relative effect size	7-9, 19
Harms	27	All harms or unintended events in each group	7-9
Ancillary analyses	28	Any other analyses performed, including subgroup and sensitivity analyses, distinguishing pre-specified from post hoc	7-9
**Discussion**			
Interpretation	29	Interpretation consistent with results, balancing benefits and harms, and considering other relevant evidence	7-9, 10-11
Limitations	30	Trial limitations, addressing sources of potential bias, imprecision, generalisability, and, if relevant, multiplicity of analyses	10-11

Citation: Hopewell S, Chan AW, Collins GS, Hróbjartsson A, Moher D, Schulz KF, et al. CONSORT 2025 Statement: updated guideline for reporting randomised trials. BMJ. 2025; 388:e081123. https://dx.doi.org/10.1136/bmj-2024-081123
© 2025 Hopewell et al. This is an Open Access article distributed under the terms of the Creative Commons Attribution License (https://creativecommons.org/licenses/by/4.0/), which permits unrestricted use, distribution, and reproduction in any medium, provided the original work is properly cited.

*We strongly recommend reading this statement in conjunction with the CONSORT 2025 Explanation and Elaboration and/or the CONSORT 2025 Expanded Checklist for important clarifications on all the items. We also recommend reading relevant CONSORT extensions. See www.consort-spirit.org.
