# Supplementary material for: An oral, liver-restricted LXR inverse agonist for dyslipidemia: preclinical development and phase 1 trial
Source: Nat Med. 2026 Jan 16;32(3):883–93. doi: 10.1038/s41591-025-04169-6 (PMC13004691; doi:10.1038/s41591-025-04169-6)
Supplement: Supplementary file 2 — Reporting Summary [file 41591_2025_4169_MOESM2_ESM.pdf]

nature portfolio

Corresponding author(s): Mani Subramanian&Johan Auwerx

Last updated by author(s): Oct 18, 2025

Reporting Summary

Nature Portfolio wishes to improve the reproducibility of the work that we publish. This form provides structure for consistency and transparency in reporting. For further information on Nature Portfolio policies, see our Editorial Policies and the Editorial Policy Checklist.

Statistics

For all statistical analyses, confirm that the following items are present in the figure legend, table legend, main text, or Methods section.

n/a	Confirmed	
☐	☒	The exact sample size (*n*) for each experimental group/condition, given as a discrete number and unit of measurement
☐	☒	A statement on whether measurements were taken from distinct samples or whether the same sample was measured repeatedly
☐	☒	The statistical test(s) used AND whether they are one- or two-sided<br>*Only common tests should be described solely by name; describe more complex techniques in the Methods section.*
☐	☒	A description of all covariates tested
☐	☒	A description of any assumptions or corrections, such as tests of normality and adjustment for multiple comparisons
☐	☒	A full description of the statistical parameters including central tendency (e.g. means) or other basic estimates (e.g. regression coefficient) AND variation (e.g. standard deviation) or associated estimates of uncertainty (e.g. confidence intervals)
☐	☒	For null hypothesis testing, the test statistic (e.g. *F*, *t*, *r*) with confidence intervals, effect sizes, degrees of freedom and *P* value noted<br>*Give P values as exact values whenever suitable.*
☒	☐	For Bayesian analysis, information on the choice of priors and Markov chain Monte Carlo settings
☐	☒	For hierarchical and complex designs, identification of the appropriate level for tests and full reporting of outcomes
☐	☒	Estimates of effect sizes (e.g. Cohen's *d*, Pearson's *r*), indicating how they were calculated

Our web collection on statistics for biologists contains articles on many of the points above.

Software and code

Policy information about availability of computer code

Data collection	The following software were used for data collection:<br>VictorX4 multiplate reader (PerkinElmer Life Science) ;<br>BMG-luminometer;<br>quantitative real-time PCR (qRT-PCR) ;<br>RNA was sequenced by BGI with the DNBSEQ platform.
Data analysis	The following software were used for data analysis:<br>r studio (v.4.4.2);<br>FastQC (version 0.11.9);<br>STAR (version 2.73a);<br>limma R package (version 3.50.3);<br>clusterProfiler R package (version 4.2.2);<br>TwoSampleMR R package (v0.6.2);<br>REGENIE;<br>Plink (genetics.binaRies R package, version 0.1.1);<br>msigdbr R package (version 7.5.1)

For manuscripts utilizing custom algorithms or software that are central to the research but not yet described in published literature, software must be made available to editors and reviewers. We strongly encourage code deposition in a community repository (e.g. GitHub). See the Nature Portfolio guidelines for submitting code & software for further information.

Data

Policy information about availability of data

All manuscripts must include a data availability statement. This statement should provide the following information, where applicable:

- Accession codes, unique identifiers, or web links for publicly available datasets
- A description of any restrictions on data availability
- For clinical datasets or third party data, please ensure that the statement adheres to our policy

The study protocol and statistical plan are available within the article and supplemental information. The raw data for preclinical experiments and the summary result for the clinical trial phase 1 data is in the source data, while the individual data from the phase 1 clinical trial are available upon reasonable request from academic or qualified clinical researchers affiliated with recognized institutions, strictly for the purpose of conducting non-commercial, ethically approvable research aligned with the original scope of the trial. Applicants are required to submit a detailed research proposal, curriculum vitae and a declaration of non-conflict of interest. Requests must clearly describe the research objectives and methodology and must be reviewed and approved by corresponding authors. All approved requestors will be required to sign a data access agreement that restricts data use solely to the approved research project and prohibits any further distribution. The HLO RNAseq data are available under the GEO numbers GSE299888.

Research involving human participants, their data, or biological material

Policy information about studies with human participants or human data. See also policy information about sex, gender (identity/presentation), and sexual orientation and race, ethnicity and racism.

Reporting on sex and gender	This study only considered sex in the data analyses. For UK biobank data, this information was provided by the UK Biobank. For clinical trial, sex was self-reporting.
Reporting on race, ethnicity, or other socially relevant groupings	For the UK biobank data, only European-ancestry participants were considered.<br>For clinical trial, we did not ask participants questions regarding race, ethnicity or other social relevant groupings
Population characteristics	For the UK biobank data, the population characteristics were provided by the UK Biobank.<br>For 50 healthy subjects in the phase 1 clinical trial, 78% subjects were male. The mean age is 29.4 ± 8.9 years and mean BMI is 24.5 ± 4.1 kg/m2. This information is also included in the Table 1.
Recruitment	For the UK biobank data, Individuals were recruited by the UK Biobank.<br>For clinical trial, individuals were evaluated by the investigator or staff at the study site. Recruitment was limited to locations or regions where the study being conducted. Written informed consent was obtained before patient enrollment. Eligible study subjects were healthy, non-smoking males and females between 18 and 55 years of age, and with BMI from 19 to 35 kg/m2, inclusive at screening. All subjects had estimated glomerular filtration rate ≥ 80 mL/min, normal liver biochemistry (total bilirubin 1.0- to 1.5-fold the upper limit of normal was permitted in subjects with Gilbert's syndrome), and 12-lead electrocardiograms (ECG) and screening laboratory evaluations (e.g., hematology, chemistry, and urinalysis) that were normal or considered to have no clinical significance by the investigator. In the MAD cohorts, an attempt was made to enroll subjects with TG ≥ 150 mg/dL and/or LDL-C ≥ 130 mg/dL to enable preliminary assessment of the lipid-lowering benefits of TLC-2716. Key exclusion criteria included pregnant or lactating females, TG ≥ 500 mg/dL, LDL-C ≥ 190 mg/dL, the presence of serious active medical or psychiatric illness, excessive alcohol consumption (defined as greater than 21 units/week for men and 14 units/week for women), substance abuse, or recent receipt of an investigational compound. Subjects who had taken any prescription or over-the-counter medications, including herbal products, within 28 days prior to the start of study drug dosing, except vitamins, acetaminophen, ibuprofen, and/or hormonal contraceptives, were excluded. A complete list of inclusion and exclusion criteria is available from the authors and study protocol.
Ethics oversight	For the UK biobank data, we have been allowed to use the UK Biobank Resource under Application Number 48020.<br>For the clinical trial, the study was approved by the Northern B Health and Disability Ethics Committee (2022 FULL 12858) and conducted at New Zealand (Auckland Clinical Research) from 2022-07-27 to 2023-06-18

Note that full information on the approval of the study protocol must also be provided in the manuscript.

Field-specific reporting

Please select the one below that is the best fit for your research. If you are not sure, read the appropriate sections before making your selection.

☒ Life sciences ☐ Behavioural & social sciences ☐ Ecological, evolutionary & environmental sciences

For a reference copy of the document with all sections, see nature.com/documents/nr-reporting-summary-flat.pdf

Life sciences study design

All studies must disclose on these points even when the disclosure is negative.

Sample size	No statistical method was used to determine sample size. Sample size was based on our previous publications: JHEP reports : innovation in

Sample size	hepatology, 5(9), 100815.; Journal of hepatology, 82(2), 174–188
Data exclusions	For the plasma biochemistry data, they might have variations and outliers should be excluded. Outliers defined by Quantile-based method were excluded. The exclusion criteria were pre-established.
Replication	For animal studies they were performed once, but included biological replicates.<br>For clinical trial, no replication was conducted.<br>All other experiments were repeated at least two times and similar results are acquired.
Randomization	In animal studies, all animals were randomly allocated into each condition<br>The first-in-human, randomized, placebo-controlled Phase 1 study (NCT05483998), conducted at a single site in New Zealand (Auckland Clinical Research), included single-ascending dose (SAD) and multiple-ascending dose (MAD) cohorts. In the SAD cohorts, healthy subjects were treated with single oral doses of TLC-2716 (0.5, 2, 6, 12, and 20 mg) or placebo, and in the MAD cohorts, subjects received once-daily oral doses of TLC-2716 (0.5, 2, 6, and 12 mg) or placebo for 14 days. For each cohort, 8 subjects were randomized to receive TLC-2716 and 2 subjects to receive placebo.
Blinding	For the animal experiments, the investigators were not blinded to group allocation during data collection and analysis. Because investigators need to provide treatment to animal models, but individuals generated the RNAseq data do not know the group information.<br>For the clinical trial, the outcome assessors and the research assistant handling the data were blinded. Computational analysis was not performed blinded.

Reporting for specific materials, systems and methods

We require information from authors about some types of materials, experimental systems and methods used in many studies. Here, indicate whether each material, system or method listed is relevant to your study. If you are not sure if a list item applies to your research, read the appropriate section before selecting a response.

Materials & experimental systems

n/a	Involved in the study
☒	☐ Antibodies
☒	☐ Eukaryotic cell lines
☒	☐ Palaeontology and archaeology
☐	☒ Animals and other organisms
☐	☒ Clinical data
☒	☐ Dual use research of concern
☒	☐ Plants

Methods

n/a	Involved in the study
☒	☐ ChIP-seq
☒	☐ Flow cytometry
☒	☐ MRI-based neuroimaging

Animals and other research organisms

Policy information about studies involving animals; ARRIVE guidelines recommended for reporting animal research, and Sex and Gender in Research

Laboratory animals	18-week old male C57BL/6 diet-induced obese (DIO) mice (14 weeks on high-fat diet, Research Diets, New Jersey, USA), purchased from Jackson Laboratories (Maine, USA); 6- to 7-week-old male obese (fa/fa) Zucker rats (ZUCKER-Leprfa), 6- to 7-week-old male Sprague Dawley rats, 6- to 8-week-old male Wistar rats bought from Charles River Laboratories; A 26-week Good Laboratory Practice (GLP) toxicology study were performed with CD-1 mice (Charles River Laboratories) and A 4-week Good Laboratory Practice (GLP) toxicology study was conducted in cynomolgus monkeys (Guangzhou Xiangguan Biotech Co., Ltd). Human liver chimeric PXB® mice were purchased from PheonexBio (Japan), and in-life procedures were performed at InterVivo Solution (Ontario, Canada).
Wild animals	Study did not involve wild animals
Reporting on sex	Male animals were used in this study for the functional test experiments to limit the number of animals. For the toxicity study, we used both male and female animals
Field-collected samples	Study did not involve field-collected samples. They were all studied in the lab.
Ethics oversight	the in-vivo studies were performed at Synovo GmbH (Tübingen, Garmany) in accordance with their bioethical guidelines, which are fully compliant to ethical regulations and internationally accepted principles for the care and use of laboratory animals;<br>the study performed at InterVivo Solution (Ontario, Canada) in accordance with their bioethical guidelines, which are fully compliant to ethical regulations and internationally accepted principles for the care and use of laboratory animals. The study performed at Physiogenex S.A.S. (Labège, France) in accordance with the ethical regulations, Guide for the Care and Use of Laboratory Animals (revised 1996 and 2011, 2010/63/EU) and French laws. All procedures involving animals were reviewed and approved by the Institutional Animal Care and Use Committee (IACUC) and conducted in accordance with international guidelines for the care and use of laboratory animals.

Note that full information on the approval of the study protocol must also be provided in the manuscript.

Clinical data

Policy information about clinical studies

All manuscripts should comply with the ICMJE guidelines for publication of clinical research and a completed CONSORT checklist must be included with all submissions.

Clinical trial registration	ClinicalTrials.gov (NCT05483998)
Study protocol	The study protocol are available in the method section and supplemental information
Data collection	This study is registered at ClinicalTrials.gov (registration: NCT05483998) and was conducted at a single site in New Zealand (Auckland Clinical Research) from 2022-07-27 to 2023-06-18, in accordance with relevant local regulatory policies. Objectively measured outcomes were collected at baseline and 14 days, including predose, 0.5, 1, 1.5, 2, 2.5, 3, 3.5, 4, 6, and 12 hours after treatment at the intervention sites by blinded assessors
Outcomes	Primary Outcome Measures:<br>(1) Number of subjects with treatment-emergent adverse events (TEAEs) in single ascending dose (SAD) compared to placebo.<br>(2) Number of subjects with clinically significant change from Baseline in vital signs in SAD.<br>(3) Number of subjects with laboratory abnormalities in SAD.<br>(4) Number of subjects with electrocardiogram (ECG) abnormalities in SAD.<br>(5) Number of subjects with TEAEs in multiple ascending dose (MAD) compared to placebo.<br>(6) Number of subjects with clinically significant change from Baseline in vital signs in MAD.<br>(7) Number of subjects with laboratory abnormalities in MAD.<br>(8) Number of subjects with ECG abnormalities in MAD.<br>Secondary Outcome Measures:<br>(1) Plasma concentration of each dose of study drug to determine AUClast in SAD.<br>(2) Plasma concentration of each dose of study drug to determine AUCinf in SAD.<br>(3) Plasma concentration of each dose of study drug to determine %AUCexp in SAD.<br>(4) Plasma concentration of each dose of study drug to determine CL/F in SAD.<br>(5) Plasma concentration of each dose of study drug to determine Cmax in SAD.<br>(6) Plasma concentration of each dose of study drug to determine Tmax in SAD.<br>(7) Plasma concentration of each dose of study drug to determine Clast in SAD.<br>(8) Plasma concentration of each dose of study drug to determine Tlast in SAD.<br>(9) Plasma concentration of each dose of study drug to determine t1/2 in SAD.<br>(10) Plasma concentration of each dose of study drug to determine λz in SAD.<br>(11) Plasma concentration of each dose of study drug to determine AUClast in MAD.<br>(12) Plasma concentration of each dose of study drug to determine AUCtau in MAD.<br>(13) Plasma concentration of each dose of study drug to determine Ctau in MAD.<br>(14) Plasma concentration of each dose of study drug to determine CLss/F in MAD.<br>(15) Plasma concentration of each dose of study drug to determine Cmax in MAD.<br>(16) Plasma concentration of each dose of study drug to determine Tmax in MAD.<br>(17) Plasma concentration of each dose of study drug to determine Clast in MAD.<br>(18) Plasma concentration of each dose of study drug to determine Tlast in MAD.<br>(19) Plasma concentration of each dose of study drug to determine t1/2 in MAD.<br>(20) Plasma concentration of each dose of study drug to determine λz in MAD.

Plants

Seed stocks	*Report on the source of all seed stocks or other plant material used. If applicable, state the seed stock centre and catalogue number. If plant specimens were collected from the field, describe the collection location, date and sampling procedures.*
Novel plant genotypes	*Describe the methods by which all novel plant genotypes were produced. This includes those generated by transgenic approaches, gene editing, chemical/radiation-based mutagenesis and hybridization. For transgenic lines, describe the transformation method, the number of independent lines analyzed and the generation upon which experiments were performed. For gene-edited lines, describe the editor used, the endogenous sequence targeted for editing, the targeting guide RNA sequence (if applicable) and how the editor was applied.*
Authentication	*Describe any authentication procedures for each seed stock used or novel genotype generated. Describe any experiments used to assess the effect of a mutation and, where applicable, how potential secondary effects (e.g. second site T-DNA insertions, mosiacism, off-target gene editing) were examined.*
